# The social leverage effect: Institutions transform weak reputation effects into strong incentives for cooperation

**DOI:** 10.1073/pnas.2408802121

**Published:** 2024-12-13

**Authors:** Julien Lie-Panis, Léo Fitouchi, Nicolas Baumard, Jean-Baptiste André

**Affiliations:** ^a^Max Planck Research Group Dynamics of Social Behavior, Max Planck Institute for Evolutionary Biology, 24306 Plön, Germany; ^b^Department of Social and Behavioral Sciences, Institute for Advanced Studies in Toulouse, Toulouse School of Economics, University of Toulouse Capitole, 31080 Toulouse, France; ^c^Institut Jean Nicod, Département d’études Cognitives, Ecole Normale Supérieure, Université Paris Sciences & Lettres, Ecole des hautes études en sciences sociales, CNRS, 75005 Paris, France

**Keywords:** cooperation, reputation, institutions, evolution, game theory

## Abstract

Institutions allow cooperation to persist when reciprocity and reputation provide insufficient incentives. Yet how they do so remains unclear, especially given that institutions are themselves a form of cooperation. To solve this puzzle, we develop a mathematical model of reputation-based cooperation in which two social dilemmas are nested within one another. The first dilemma, characterized by high individual costs or insufficient monitoring, cannot be solved by reputation alone. The second dilemma, an institutional collective action, involves individuals contributing to change the parameters of the first dilemma in a way that incentivizes cooperation. Our model demonstrates that this nested architecture creates a leverage effect. While insufficient on its own to incentivize cooperation in the first dilemma, reputation incentivizes contributions to the institutional collective action, which, in turn, strengthen the initially weak incentives for cooperation in the first dilemma. Just as a pulley system transforms minimal muscular strength into significant lifting capability, institutions act as cooperative pulleys, transforming initially weak reputational incentives into powerful drivers of cooperative behavior. Based on these results, we suggest that institutions have developed as social technologies, designed by humans to exploit this social leverage effect, just as material technologies are designed to exploit physical laws.

## Significance

Institutions explain why humans exhibit such high levels of cooperation compared to other species. From small communities to large nation-states, they promote cooperation by rewarding prosocial conduct and punishing acts of selfishness. Yet institutions are themselves cooperative enterprises—their effectiveness depends on people’s willingness to participate in assemblies and resist corruption. How, then, can institutions promote cooperation when they rely on it? We show that institutions can leverage the power of reputation. Reputation encourages individuals to contribute to institutions, which transform contributions into new incentives. If generated efficiently, these institutional incentives unlock cooperation in scenarios where reputation alone would be insufficient. Thus, institutions can transform initially weak cooperative tendencies into strong incentives for cooperation.

Large-scale cooperation is central to the success of the human species (1). Yet its origins remain poorly understood. Canonical explanations, such as kin altruism (2, 3), reciprocity (4–6), and reputation (7–12), seem insufficient to explain the scale and intensity of human cooperation. In large human societies, more often than not, partners are unrelated, interactions are one-shot, and reputational information is narrowly disseminated (13, 14).

The social sciences have long recognized that institutions play a crucial role in surmounting these challenges. Humans have designed social organizations such as clans (15), age sets (16), merchant guilds (17), assemblies (18), governments (19), and justice systems (20–22), that make rules of good behavior explicit, specify role-specific obligations, and organize the monitoring and punishment of free-riders (23, 24). Essentially, these organizations solve the free-rider problem by instituting new incentives for cooperation (25, 26).

Institutions, however, are themselves cooperative enterprises, and as such they face a second-order free-rider problem (27–30). People must devote time and resources to create new rules and pay institutional operatives. These operatives, in turn, must resist corruption; they must, for instance, rebuff bribes (31) and avoid abuses of power (32). In other words, saying that institutions stabilize cooperation seems to only push the problem one step further: what stabilizes institutions?

In this paper, we present a mathematical model of institutions, that sheds light on how they can stabilize cooperation while themselves relying on cooperation. We show that institutions do more than just push the problem one step further; they can solve it. This solution is achieved through a social leverage effect that arises from the nesting of multiple collective actions within one another.

Our premise is that cooperative dilemmas vary in difficulty. Some cooperative dilemmas are hard; because the temptation to cheat is high, because cheaters are unlikely to be observed, or because the dilemma involves many unrelated individuals. Other cooperative dilemmas are easy; because cooperation is cheap, behaviors are observable, and interactions occur within small groups of kith and kin.

**Fig. 1.**
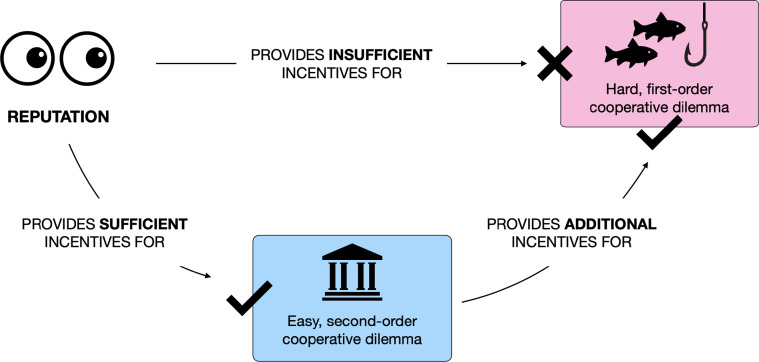
Institutions allow reputation to solve hard cooperation problems indirectly. Reputation can solve hard cooperation problems indirectly, by incentivizing an easier form of second-order cooperation, which in turn increases the incentive to cooperate at the first order. By engineering an institution based on such a form of second-order cooperation, humans engineer a technological solution to a hard cooperation problem, using only the limited reputational incentives at their disposal.

Humans need not tackle hard cooperation problems head on. Instead, they can design another cooperative interaction that is easier to solve (e.g., because behaviors are more observable), and that generates new incentives for cooperation in the hard dilemma (e.g., by organizing the monitoring of free-riding). Institutions, we argue, consist of these easy dilemmas, within which hard cooperation problems are embedded. If the cost of institutional cooperation is low enough to be driven solely by reputational concerns, and the institution generates enough new incentives to solve the initial hard dilemma, cooperation becomes indirectly solvable through reputation (Fig. 1). By creating a nested architecture of dilemmas, institutions create a leverage effect that can amplify the power of reputation, analogous to how levers amplify physical forces.

Take a historical example. In rural Japan, villagers needed to cooperate to preserve communal forests from overuse (28, pp. 65–69) (33). This cooperation problem was hard: it was strongly in each villager’s interest to overuse the communal forest, and it was difficult to check that no one was doing so. To solve this hard problem, villages hired specialized monitors called detectives, whose job was to spot and impose fines on free-riders, thereby generating new incentives for cooperation. This institution was itself a cooperative enterprise: for the whole thing to work, detectives had to do their job faithfully, instead of soliciting bribes or exacting unfair penalties. Yet the underlying cooperation problem was easier: if they abused their power, detectives were likely to be spotted, and, thus, to lose their hard-earned reputation. By hiring detectives, the villagers had found a way to solve their hard problem indirectly, using only the limited reputational incentives at their disposal.

We formalize this idea using the mathematical model below. Our model focuses on individuals called actors who can cooperate in two different ways: sometimes, they can pay to reciprocate the trust of a chooser, and sometimes they can pay to contribute to an institution. In both cases, the only benefit they gain is reputational. Each time actors are observed reciprocating or contributing, they enhance their reputation, and become more likely to be trusted by choosers in the future.

The institution collects individual contributions, and transforms them into incentives for cooperation between actors and choosers. We show that the institution extends the domain of reputation-based cooperation, to include hard cooperation problems. What’s more, we show that the amount of additional cooperation generated by the institution varies with its efficiency—the amount of incentives the institution produces for every resource unit it receives. This underscores the idea that institutions should be viewed as a social technology. Just as a pulley system helps lift heavy loads with minimal effort, institutions maximize the potential of reputational incentives, helping humans address hard cooperation problems that reputation could not solve directly. Institutions appear as social engineering tools that humans have invented and gradually refined to build the most mutually beneficial social organizations that can be sustained by reputation alone.

## Model

### A Model of Reputation-Based First- and Second-Order Cooperation.

We model a repeated game between a large number n≫1 of actors and an infinite pool of choosers. In each round, n choosers are randomly drawn and matched with different actors. After the round’s interactions, the choosers exit the game, while the actors proceed to the next round. Thus, actors are long-lived, participating in every round, while choosers are short-lived, interacting only once (our framework is inspired by ref. 34).

Each actor is defined by a type, specifically a discount factor δ (0<δ<1), which determines how much the actor values future payoffs. This factor remains hidden from other players. Actors discount future rewards according to their δ, with the present value of a payoff unit to be received in t rounds being $\delta^{t}$. A higher δ reflects a more patient actor. Discount factors are drawn at birth from a continuous distribution with full support over the interval (0,1), allowing for a diversity of time preferences among actors.

**Fig. 2.**
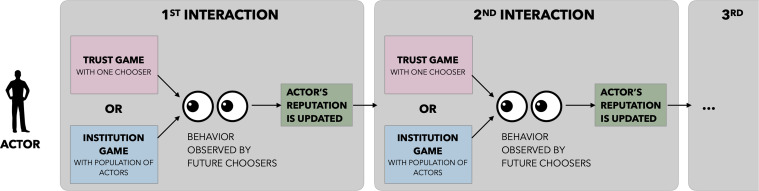
Life of an individual actor. Throughout their life, actors engage in infinitely many interactions. These interactions either involve a chooser in a trust game or involve the population of actors in the institution game (both described below). In both cases, future choosers may observe their behavior, and actors’ reputations are updated accordingly.

Actors engage in two different interactions (Fig. 2). In each round, they either play a trust game with their assigned chooser, with probability q (0<q<1), or participate in the institution game, with probability 1−q. In expectation qn actors play as many trust games, while the remaining (1−q)n actors take part in the institution game.

**Fig. 3.**
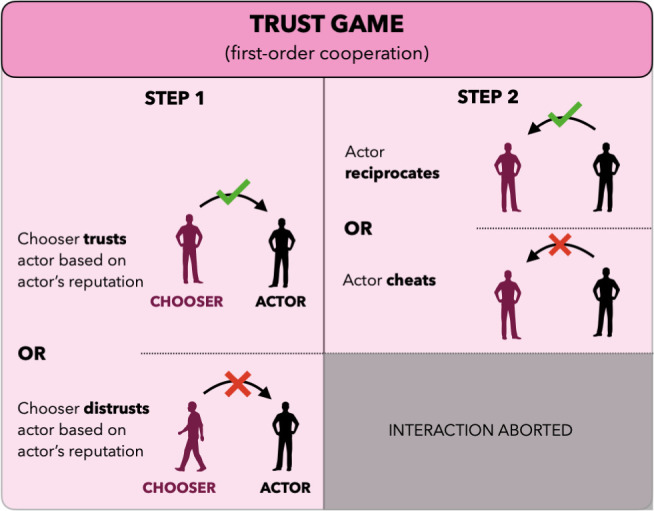
Trust game. In a trust game, an actor interacts with a chooser. The chooser first decides whether to trust the actor, based on their reputation. If the chooser trusts, the actor then decides whether to reciprocate that trust or betray it by cheating.

In each trust game, one actor interacts with one chooser (Fig. 3). The chooser first decides whether to trust or distrust the actor. Trust costs the chooser k>0 and rewards the actor with r>0. If trusted, the actor then chooses whether to reciprocate or cheat the chooser. Reciprocation costs the actor $c_{1}>0$ and provides the chooser with a benefit b>k.

**Fig. 4.**
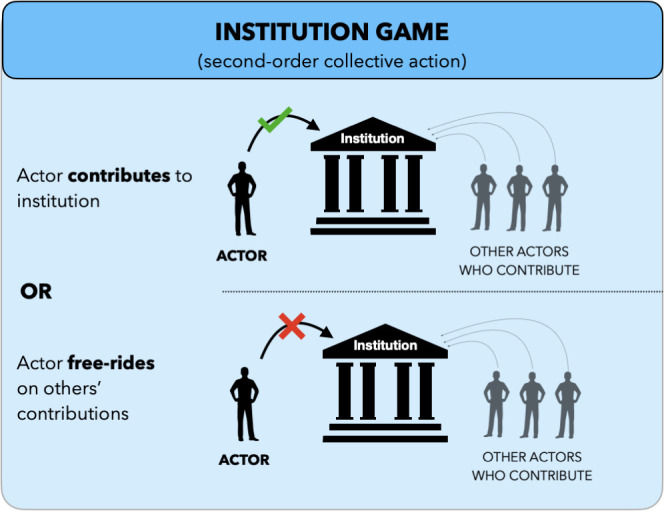
Institution game. In the institution game, actors join a collective action. Each actor chooses to either contribute or free-ride on the contributions of others. Contributions are used to incentivize reciprocation in trust games (Fig. 5).

In the institution game, actors take part in a collective action (Fig. 4). Each of them decides whether to contribute or free-ride on others’ contributions. Contributing costs $c_{2}>0$.

**Fig. 5.**
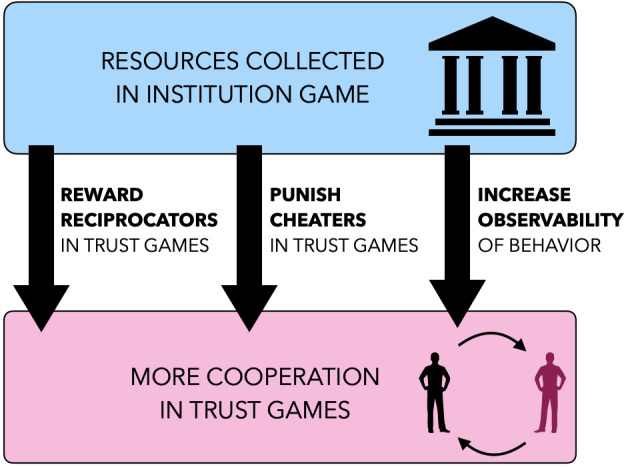
Mechanism of the institution. The institution transforms contributions made in the institution game into incentives for reciprocation in trust games, facilitating cooperation between actors and choosers. A portion of the contributions rewards reciprocators, another punishes cheaters, and the remainder is allocated to monitoring.

Contributing to the institution is a form of second-order cooperation (Fig. 5). As described below, the institution uses contributions to incentivize reciprocation in the trust games occurring in parallel. In our model, the only motivation to contribute is reputational—contributors do not benefit from institutional incentives (in contrast to e.g., ref. 35), as these incentives affect interactions that the contributors themselves are not a part of. Instead, contributions indirectly encourage other actors to reciprocate choosers’ trust and motivate choosers to trust in the first place. Throughout the text, we refer to contribution as second-order cooperation, and to a chooser trusting an actor who then reciprocates as first-order cooperation, or simply cooperation.

Actors’ choices shape their reputation. To simplify, we assume that choosers only observe behavior from the previous round, if any, with baseline probability $p_{1}$ for actors involved in a trust game ($0<p_{1}<1$; this probability may be increased through the institution), and fixed probability $p_{2}$ for actors involved in the institution game ($0<p_{2}<1$). An actor’s reputation is updated each round and can take one of only five values: reciprocator, cheater, contributor or free-rider—if the actor was observed playing the corresponding action—or empty, if the actor was not observed or did not play.

A pure actor strategy specifies whether to reciprocate or cheat in a trust game and whether to contribute or free-ride in the institution game, based on the actor’s reputation and discount factor. As we will see, in equilibrium, more patient actors are more likely to reciprocate their partner’s trust and contribute to the institution, since both involve paying immediate costs to obtain future reputational benefits.

A pure chooser strategy specifies whether to trust or distrust, depending on the actor’s reputation. In our model, reputation informs a risky decision. Choosers use an actor’s reputation to predict whether they will reciprocate their trust. This approach aligns with models in the signaling or reputation-based partner choice tradition, but contrasts with models in the indirect reciprocity tradition (36, 37).

**Fig. 6.**
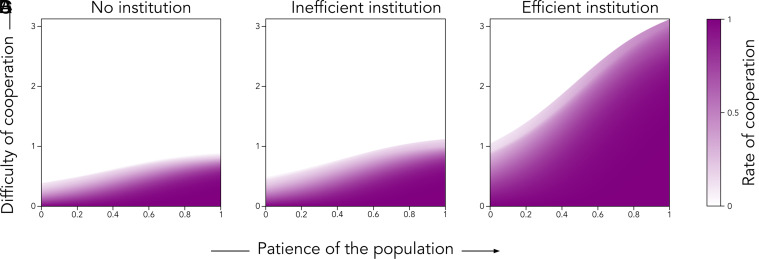
Rate of cooperation. The rate of cooperation measures how frequently actors and choosers successfully cooperate over the long run. Specifically, it is the probability that, after many rounds of the game, a randomly selected chooser trusts a randomly selected actor based on their reputation, and the actor reciprocates. We calculate this rate as a function of the patience of the population (μ, x-axis, ranging from 0 to 1) and the difficulty of cooperation ($\delta^{b}$, y-axis, ranging from 0 to 3.25). The rate is depicted by a gradient from white (0, indicating no cooperation) to purple (1, indicating full cooperation). We explore three scenarios: (*A*) no institution (baseline equilibrium), (*B*) an inefficient institution (institution equilibrium with ρ=1/3), and (*C*) an efficient institution (institution equilibrium with ρ=3). The institution allocates incentives equally between punishing cheaters and monitoring trust games (β=0, $\gamma =\left(1/2\right)\rho c_{2}\left(1-q\right)n_{2}/\left(qn\right)$, $\pi_{1}=\left(1/2\right)\rho \left(c_{2}/c_{1}\right)\left(1-q\right)n_{2}/\left(qn\right)$). Actors’ time preferences follow a truncated normal distribution with mode μ (varying from 0 to 1) and SD σ=0.25. Fixed parameters include the probability of facing a trust game (q=0.5), the benefit of being trusted (r=2), and the benefit from reciprocation (b=1). In trust games, actors are observed with low baseline probability ($p_{1}=0.25$), while in the institution game, they are observed three times more often ($p_{2}=0.75$). As we vary the difficulty of cooperation ($\delta^{b}$), we vary the cost of reciprocation ($c_{1}=\left(p_{1}qr\right)\delta^{b}=\delta^{b}/4$), the cost of trust (set at $k=c_{1}$) and the cost of contribution (set at $c_{2}=c_{1}/3$). Without institutional incentives, actors and choosers face similar costs in trust games ($c_{1}/\left(qr\right)=k/b$). Contributing to the institution is initially three times cheaper than reciprocating a chooser’s trust $\left(c_{2}=c_{1}/3\right)$.

**Fig. 7.**
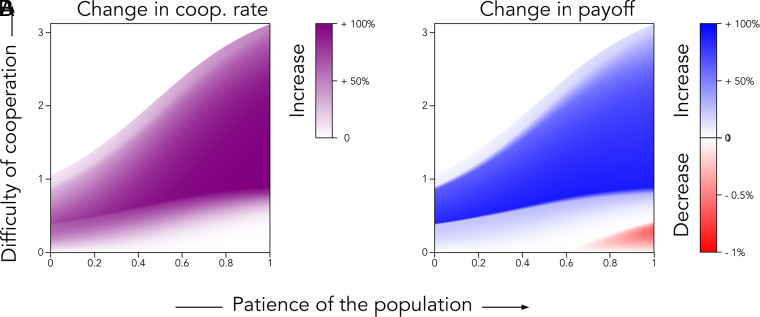
Comparison between an efficient institution and no institution. We compare (*A*) the rate of cooperation and (*B*) the expected payoff between two scenarios: no institution (baseline equilibrium) and an efficient institution (institution equilibrium with ρ=3). The rate of cooperation is defined as in Fig. 6, with increases represented by a gradient from white (0% increase) to purple (100% increase). The expected payoff is the normalized payoff of a randomly selected individual, considering both actors and choosers. For actors, this is their average lifetime payoff; for choosers, the payoff of one interaction is measured after many rounds of the game. We use a gradient from white (0% increase) to blue (100% increase) to show increases in expected payoff, and from white (0% decrease) to red (1%) to indicate decreases. These small decreases (up to 1%) occur because the cost of contributing to the institution is minimal in regions where the institution is unnecessary. Specifically, when cooperation is easy (low $\delta^{b}$), the cost of contribution is very small ($c_{2}=c_{1}/3=\delta^{b}/12$), and is only incurred half the time (with probability 1−q=0.5). As in Fig. 6, the institution allocates incentives equally between punishing cheaters and monitoring trust games. We use the same parameter values and variables.

We restrict our analysis to pure actor strategies, allowing choosers to mix only when deciding whether to trust actors with empty reputation. Choosers trust these actors with probability θ (0≤θ≤1), and otherwise behave deterministically. This approach smooths the depiction of cooperation rates and payoffs in Figs. 6 and 7.

### Mechanism of the Institution.

The institution collects contributions from actors. In a given round, let $n_{2}$ represent the number of potential contributors—those actors who would contribute if given the opportunity. Since each actor faces the institution game with probability 1−q and each contribution is worth $c_{2}$, the institution is expected to receive contributions totaling $\left(1-q\right)n_{2}c_{2}$.

The institution transforms contributions into incentives for reciprocation. Incoming contributions are multiplied by a factor ρ>0, which represents the institution’s efficiency. For every unit of resource it receives, the institution generates ρ units of incentives. On average, the institution produces incentives totaling $\rho \left(1-q\right)n_{2}c_{2}$.

These incentives are distributed evenly across all trust games played that round. A portion rewards reciprocators, another punishes cheaters, and the remainder is allocated to monitoring. Specifically, in each trust game, the payoff for reciprocating increases by β≥0, the payoff for cheating decreases by γ≥0, and the probability of observation rises by $\pi_{1}\geq 0$. Summing these effects gives $\beta +\gamma +c_{1}\pi_{1}$ per trust game, where $c_{1}$ is an arbitrary conversion factor that translates the probability increase $\pi_{1}$ into resource units. On average, with qn trust games being played, the incentives produced by the institution total $qn(\beta +\gamma +c_{1}\pi_{1})$.

By equating both formulas for the incentives generated by the institution and dividing by qn on both sides of the equation, we obtain

$$\begin{array}{c} \beta +\gamma +c_{1}\pi_{1}=\rho c_{2}\frac{\left(1-q\right)n_{2}}{\mathrm{qn}}. \end{array}$$

This general model allows us to explore different types of institutions by adjusting parameter values. For instance, a (purely) punishing institution is created by setting $\beta =\pi_{1}=0$. In this case, every unit of resources collected by the institution is converted into a penalty for every actor who cheats the trust of their assigned chooser, equal to $\gamma =\rho c_{2}\left(1-q\right)n_{2}/\left(qn\right)$. A monitoring institution is formed by setting β=γ=0, in which case, the probability of observation in every trust game increases by $\pi_{1}=\rho \left(c_{2}/c_{1}\right)\left(1-q\right)n_{2}/\left(qn\right)$. Finally, a rewarding institution is obtained by setting $\gamma =\pi_{1}=0$.

Taking the institution’s effect into account, we calculate the net cost of reciprocation by subtracting the total payoff of reciprocating from the total payoff of cheating, which yields: $\left(r-\gamma \right)-\left(r-c_{1}+\beta \right)=c_{1}-\beta -\gamma$. The total probability of observation in trust games is equal to $p_{1}+\pi_{1}$. We assume that even after accounting for the institution, reciprocation remains costlier and less observable than contribution; that is, that: $c_{2}\leq c_{1}-\beta -\gamma$ and $p_{1}+\pi_{1}\leq p_{2}$.

## Results

### Equilibrium Analysis.

We analyze our model by characterizing all possible endpoints of an evolutionary process. To do so, we use the concept of a perfect Bayesian equilibrium, or PBE. The PBE is a refinement of the Nash equilibrium that applies to games with multiple interactions and hidden types, like the one we have presented. It ensures that a strategy profile is sensible (38). If a strategy profile fails to meet the criteria of a PBE, some players could deviate profitably, and their behavior would spread if strategies were evolving.

In our model, actors have hidden types—their discount factor δ—while choosers have partial information about actors’ past actions through their reputation. In the PBEs described below, actors’ behavior is driven by their discount factor, which means that their reputation will convey information about their δ. This allows choosers to make informed trust decisions. These equilibria are sustained as long as nonempty reputations reliably predict whether actors will reciprocate.

#### Baseline equilibrium: Cooperation in the absence of an institution.

To establish a baseline, we remove the institution by assuming choosers do not observe second-order cooperation; that is, by setting $p_{2}=0$. This makes the institution irrelevant. In equilibrium, actors never contribute to the collective action, since doing so is costly and offers no reputational benefits. We show that there exists a unique PBE in which cooperation occurs, which we call the baseline equilibrium.

In the baseline equilibrium, reputation incentivizes reciprocation. Choosers trust actors who are reputed to be reciprocators, while distrusting those labeled as cheaters. Additionally, choosers trust actors with an empty reputation with a certain probability θ, whose value is given in the *Materials and Methods* section at the end of this document.

Patient actors always reciprocate their partners’ trust, while impatient actors always cheat. Regardless of reputation, an actor with discount factor δ reciprocates if $\delta \geq {\hat{\delta }}^{b}\left(\theta \right)$ and cheats if $\delta <{\hat{\delta }}^{b}\left(\theta \right)$. The threshold separating reciprocators from cheaters is given by

[B.1] $$\begin{array}{c} {\hat{\delta }}^{b}\left(\theta \right)=\frac{c_{1}}{p_{1}q\left(r-\theta c_{1}\right)}. \end{array}$$

In the most favorable case, where θ=0, the threshold simplifies to $c_{1}/\left(p_{1}qr\right)$. We refer to this minimum value as the difficulty of cooperation. This value, denoted by $\delta^{b}$ (without a hat), increases as reciprocation becomes more costly or less observable. As $\delta^{b}$ rises, it becomes harder for actors to reciprocate, for choosers to trust, and for cooperation between them to occur.

The baseline equilibrium exists as long as reputations are reliable predictors of actor behavior, guiding chooser trust. Since actors follow stationary strategies, it is enough for reciprocators and cheaters to exist with positive probability—past reciprocation then perfectly predicts future reciprocation. Given that discount factors are continuously distributed over the interval (0,1), the equilibrium holds if $0<{\hat{\delta }}^{b}\left(\theta \right)<1$.

#### Institution equilibrium.

When choosers do observe second-order cooperation ($p_{2}>0$), another PBE becomes possible. We call this equilibrium the institution equilibrium.

In the institution equilibrium, reputation incentivizes both reciprocation and contribution. Choosers trust actors who are reputed to be reciprocators as well as contributors, while distrusting those who are labeled as cheaters or free-riders. As in the baseline equilibrium, they trust actors with an empty reputation with a certain probability θ, whose value is given in the *Materials and Methods*.

Actors reciprocate and contribute based on their discount factor. Regardless of reputation, an actor with discount factor δ reciprocates if $\delta \geq {\hat{\delta }}_{1}\left(\theta \right)$ and cheats if $\delta <{\hat{\delta }}_{1}\left(\theta \right)$. The actor contributes if $\delta \geq {\hat{\delta }}_{2}\left(\theta \right)$ and free-rides if $\delta <{\hat{\delta }}_{2}\left(\theta \right)$. The thresholds separating reciprocators from cheaters and contributors from free-riders are given by

[I.1] $${\hat{\delta }}_{1}(\theta )=\frac{c_{1}-\beta -\gamma }{\left(p_{1}+\pi_{1})q[r-\gamma -\theta (c_{1}-\gamma -\beta )\right]},$$

[I.2] $${\hat{\delta }}_{2}(\theta )=\frac{c_{2}}{q[p_{2}(r-\gamma )-(p_{1}+\pi_{1})\theta c_{2}]}.$$

From our conditions, we deduce ${\hat{\delta }}_{1}\left(\theta \right)<{\hat{\delta }}^{b}\left(\theta \right)$. Any institution lowers the threshold for reciprocation, regardless of how rewards, punishment, and monitoring are balanced (i.e., the values of β, γ, and $\pi_{1}$).

We also deduce ${\hat{\delta }}_{2}\left(\theta \right)\leq {\hat{\delta }}_{1}\left(\theta \right)$. Since contribution is less costly ($c_{2}\leq c_{1}-\left(\beta +\gamma \right)$) and more observable ($p_{2}\geq p_{1}+\pi_{1}$) than reciprocation, it has a lower threshold. Some actors with intermediate patience contribute without reciprocating, but all reciprocators also contribute.

Like the baseline equilibrium, the institution equilibrium exists as long as reputations reliably predict actor behavior. A necessary condition comes from considering a reputed contributor. On average, trusting such an actor yields payoff −k+P(reciprocates∣contributor)×b. Distrusting yields payoff 0. By comparing the two payoffs, we deduce that the predictive value of contribution $P\left(\;\text{reciprocates}|\;\text{contributor}\right)=P(\delta \geq {\hat{\delta }}_{1}\left(\theta \right)|\delta \geq {\hat{\delta }}_{2}\left(\theta \right))$ must be larger than the relative cost of trust (k/b).

### Numerical Solution.

To illustrate our results, we fix both the institution and the distribution of discount factors. We assume a normal distribution of mode μ and SD σ, truncated over the interval (0,1) (0<μ<1, 0<σ<1). When μ is high, most individual actors are patient. We refer to μ as the patience of the population.

We focus on an institution that does not reward reciprocators (β=0) and instead allocates incentives equally between punishing cheaters and monitoring trust games ($\gamma =\left(1/2\right)\rho c_{2}\left(1-q\right)n_{2}/\left(qn\right)$ and $\pi_{1}=\left(1/2\right)\rho \left(c_{2}/c_{1}\right)\left(1-q\right)n_{2}/\left(qn\right)$). In *SI Appendix*, we explore other institutional designs and verify that they lead to similar results.

Using Mathematica, we compute equilibrium outcomes in three cases: a) the baseline equilibrium, where choosers do not observe second-order cooperation by definition ($p_{2}=0$), b) the institution equilibrium with an inefficient institution (ρ=1/3), and c) the institution equilibrium with an efficient institution (ρ=3). Fig. 6 shows the rate of cooperation in each of these three cases, as a function of the patience of the population μ on the x-axis, and the difficulty of cooperation $\delta^{b}$ on the y-axis.

#### Efficient institutions extend the domain of cooperation.

Without institutional support, reputation cannot solve hard cooperation problems. As shown in panel (*A*) of Fig. 6, cooperation rates in the baseline equilibrium quickly drop to zero as the difficulty of cooperation $\delta^{b}$ increases. Cooperation becomes impossible once $\delta^{b}\geq 1$.

Efficient institutions, however, extend the domain of reputation-based cooperation, to include even hard problems. As shown in panel (*C*) of Fig. 6, with an efficient institution, cooperation rates remain positive even when the difficulty of cooperation exceeds 1. In some cases, cooperation persists even when $\delta^{b}>3$. By contrast, panel (*B*) of Fig. 6 shows that an inefficient institution (ρ=1/3) has only a marginal effect on cooperation rates.

#### Institutional success depends on the population being patient.

While institutional efficiency is crucial, it alone does not guarantee cooperation. People also need to be motivated to contribute to the institution, which requires them to engage in second-order cooperation. In the model, the institution generates incentives for cooperation in proportion to its efficiency ρ, the number of contributors $n_{2}$, and the value of each contribution $c_{2}$. Since contributions are relatively small by assumption ($c_{2}=c_{1}/3$ in our numerical solution), high efficiency (ρ) and a large number of contributors ($n_{2}$) are necessary to create strong enough incentives for solving hard cooperation problems.

Returning to panel (*C*) of Fig. 6, we observe that positive cooperation rates are sustained even in hard cooperation problems when the population is patient (high μ) in addition to the institution being efficient (ρ=3). This is because μ is indicative of individuals’ intrinsic motivation to cooperate, which in our model means accepting immediate costs (to reciprocate or contribute) in exchange for future benefits (increased trust from future choosers). As μ increases, the number of contributors grows, boosting the institution’s capacity to generate sufficient incentives for cooperation.

#### Institutions become wasteful when cooperation is easy and the population is very patient.

When the population is highly patient (high μ) but cooperation is easy (low $\delta^{b}$), institutions become unnecessary. In such cases, high cooperation rates are already achieved in the baseline equilibrium, without institutional support. Paying for institutional monitoring or punishment then becomes wasteful.

To illustrate, we subtract the rate of cooperation in the baseline equilibrium from that in the institution equilibrium with an efficient institution (ρ=3). The resulting difference is shown in panel (*A*) of Fig. 7. We also compare expected individual payoffs, as shown in panel (*B*). When cooperation is easy and the population is highly patient, the institution provides only a marginal increase in cooperation. As a result, individuals are worse off.

## Discussion

You can set up British-style courts of law, and even provide the barristers with wigs, but if the judges are venal and the barristers have no professional pride and if the public disdains them both, then the introduction of such a nice-sounding institution will fail to improve the rule of law. (39, chapter 15)

Humans rely on institutions to stabilize cooperation. Yet, as McCloskey vividly illustrates, institutions are no magic bullet. Institutions require more than just sound structures; they hinge on the people within them, whose personal interests will inevitably clash with the common good. They are, in other words, second-order cooperative interactions—cooperative interactions aimed at promoting cooperation—which emerge from the very communities they are supposed to regulate.

In existing models, institutions are sustained either without individuals incurring personal costs or through competition between groups. Many models assume that institutions can be enforced at no individual cost—whether because enforcement is in the interest of powerful leaders (40, 41), or because everyone commits to either reward enforcers (42, 43) or punish nonenforcers (44) (see also refs. 35, 45, and 46). In other models, institutions are maintained through competition between groups (47–51). Enforcers pay costs that are never recouped, putting them at a selective disadvantage within their group. However, groups with institutions tend to outcompete groups without them.

In contrast, our model excludes group competition, yet the institution still requires individual cooperation to function. Our model is built around two nested cooperative interactions. Actors can sometimes pay to reciprocate the trust of a chooser, and they can sometimes pay to contribute to an institution. The institution pools all individual contributions, and transforms them into incentives for reciprocation (e.g., by punishing individuals who cheat on a chooser’s trust).

We show that this nested architecture can create a social leverage effect. By nesting a hard dilemma within an easy one, the institution offers an indirect solution to the hard cooperation problem. For this solution to be effective, the institution must also be efficient. Efficient institutions allow reputation to indirectly stabilize cooperation in hard dilemmas, by embedding them within an easier, solvable dilemma, and still generating enough incentives based on the easy dilemma.

Our model is kept simple. In particular, individuals vary only in their time preferences and can either contribute to institutional efforts or choose to free-ride. Further research could employ dynamic methods (for a review, see ref. 52) to complement our equilibrium analysis or examine the robustness of our results in more complex scenarios, for instance, by allowing individuals to subvert institutional incentives for personal gain or by introducing other sources of variation, such as differences in endowment or power.

Our model generates distinctive predictions for how institutions form and stabilize in human societies. In the following, we detail the model’s predictions, and show that they are supported by evidence from across the psychological and social sciences.

### Institutions Require Intrinsic Honesty and Social Capital.

For the institution to work in our model, individuals need to cooperate. The more willing they are to shoulder the costs of second-order cooperation, the better the institution can push others to cooperate at the first order. This is consistent with a large body of evidence from psychology (31, 53), economics (54, 55), and political science (39, 56): well-functioning institutions require the costly cooperation of individuals, who must resist second-order free-riding (e.g., corruption) for the institution to successfully promote cooperation.

Consequently, our model predicts that institutions will be most successful in promoting cooperation in populations that are already predisposed toward cooperation. In line with this prediction, across 23 societies, institutional quality is associated with people’s intrinsic honesty—people’s propensity to cooperate even when they are not incentivized by institutions to do so (57). Further supporting this, in a famous study, Putnam et al. (58) showed that the best predictor of institutional performance across Italian regions was people’s propensity to engage in grassroots cooperative interactions such as sports clubs, literary guilds, or choral societies. Putnam explained this association in terms of social capital—the social networks and norms of reciprocity that emerge from a long history of grassroots cooperation. The importance of social capital for institutional functioning replicates in other geographic areas and historical periods (59–65).

### Institutional Honesty Depends on Reputational Incentives.

If institutional quality depends on intrinsic honesty, what compels agents to be honest in the first place? In line with previous models (66–68) and experimental evidence (69–71), our model suggests that reputational incentives can drive institutional cooperation.

In the real world, individuals who take on an institutional role are indeed motivated by reputation and social rewards. In her famous review, Ostrom underlines how, in communities that create long-lasting institutions for common-pool resources, monitors are incentivized through reputation: “The individual who finds a rule-infractor gains status and prestige for being a good protector of the commons” (28, p. 96). Similar dynamics can be found in nonindustrial societies. Among the Enga of Papua New Guinea, for example, mediators who resolve conflicts in customary courts gain a good reputation (72). Among the Amazonian Tsimane, similarly, men who mediate more conflicts are more frequently cited as cooperation partners (73). More largely, across nonindustrial societies, informal leaders tend to resolve conflicts on the one hand, and enjoy high status on the other (74).

### Reputation-Based Institutions Develop in Future-Oriented Populations.

If reputation drives institutional cooperation, what drives variation in institutional quality? Why does reputation, in many societies, manifestly fail to limit corruption? Our model suggests that this variability can be analyzed in terms of time preferences.

In the model, both first- and second-order cooperation involve a present-future trade-off—cooperative individuals pay to acquire a good reputation today, and increase their chances of being trusted tomorrow. As a result, future-oriented individuals are more likely to engage in either form of cooperation, and future-oriented populations are more likely to sustain the institution.

Consequently, our model predicts that better-functioning institutions should emerge in more future-oriented populations. This allows us to put two stylized facts in perspective. First, time preferences allow us to revisit the importance of social capital for institutional functioning (58). As Putnam explains, a long history of cooperation makes social capital. It also makes the future loom large. In communities with strong social networks and norms of reciprocity, individuals can expect more from their cooperative future. With respect to their reputation, they can be characterized as patient.

Our model also explains why material circumstances matter for institutional quality. In more affluent environments, individuals’ most pressing needs are already met, allowing them to explore other opportunities, like investing in their reputation or social network (75, 76). Thus, all other things being equal, individuals in more affluent environments should be more patient, and more able to trust that others will also invest in their cooperative reputation. Supporting this, experimental evidence shows that political leaders are more corrupt when their voters are poor (77), and that poorer individuals more often have to pay bribes to government officials (78). At the macroscopic level, a country’s level of corruption is negatively associated with its wealth (79–81). It should be noted, however, that the relationship is bidirectional (82, 83). While economic hardship paves the way for enduring corruption (84), corrupt institutions can also lead to economic hardship (32).

### Social Engineering and the Cultural Evolution of Institutions.

Last, our model contributes to understanding the cultural evolution of institutions. It illustrates how institutions can harness the social leverage effect—by nesting a hard dilemma within an easy one, the institution in our model offers an indirect solution to the hard dilemma. Put differently, it creates the possibility of stabilizing costly forms of cooperation with only weak reputational incentives. Thus, institutions appear as technologies that exploit social laws, just as material technologies exploit physical laws. They have been likely designed, and gradually refined, to build the most mutually beneficial social organizations that can be sustained by reputation alone.

For the social leverage effect to function, however, the institution must be sufficiently efficient—it needs to generate enough incentives for the hard dilemma using resources coming from the easy dilemma. The cultural evolution of institutions may have unfolded as humans discovered more efficient institutional arrangements, allowing them to exploit higher leverage, and expand the scope of cooperation. One way to maximize leverage, for example, is to assign monitoring and punishing duties to only a small group of specialized individuals, to ensure that deviations are easy to spot and identify (85). Accordingly, many real-world institutions rely on specialized monitors (28), and experimental evidence suggests that people prefer to delegate punishment decisions (86). Another way to maximize leverage is to rely on an increasingly nested architecture. Many institutional arrangements, particularly in large-scale societies, group individuals into lower-level units, ensuring that reputation can continue to act as a strong incentive even as the number of total individuals increases (14).

## Materials and Methods

### Model Description

#### Repeated Game.

We model a repeated game between a large number n≫1 of actors and an infinite pool of choosers. Actors participate in every round. They are each characterized by a hidden type—a discount factor δ (0<δ<1) whose value is drawn at the beginning of the game according to a continuous distribution of full support. Choosers participate in only one round of interaction.

#### Stage Game.

In each round, actors play a trust game with a randomly assigned chooser, with probability q. The chooser decides whether to trust or distrust, and, if trusted, the actor decides whether to reciprocate or cheat. Trust costs the chooser k and benefits the actor by r; reciprocation costs the actor $c_{1}$ and benefits the chooser by b>k.

Actors who do not draw a trust game play the institution game. Each of them decides whether to pay $c_{2}>0$ to contribute to the institution, or free-ride on others’ contributions.

#### Reputation.

Actors’ choices are observed with baseline probability $p_{1}$ in trust games, and fixed probability $p_{2}$ in the institution game. An actor’s reputation indicates their observed behavior in the previous round, if any. It is reciprocator, cheater, contributor or free-rider if the actor was observed playing the corresponding action, and empty otherwise.

#### Mechanism of the Institution.

In each round, the institution collects contributions made in the institution game, and multiplies them by an efficiency parameter ρ. It uses multiplied contributions to incentivize reciprocation. In every trust game occurring that round, the payoff for reciprocation increases by β, the payoff for cheating decreases by γ, and the probability of observation increases by $\pi_{1}$.

#### Strategies.

A pure actor strategy specifies whether to reciprocate or cheat in trust games and whether to contribute or free-ride in the institution game, as a function of the actor’s reputation and discount factor. A pure chooser strategy specifies whether to trust or distrust an actor as a function of their reputation. We restrict our analysis to pure actor strategies, and allow choosers to mix only when deciding whether to trust actors with empty reputation.

#### Beliefs.

In models with incomplete information, players form beliefs about others’ type—here, choosers form beliefs about an actor’s type δ. In a perfect Bayesian equilibrium (PBE), choosers’ beliefs are updated depending on the actor’s reputation, using Bayes’ rule when possible.

As we detail in *SI Appendix*, section 3.2, an issue here is that choosers do not know which round t they are playing in, but that the predictive value of empty reputation changes with time. Initially, reciprocators and cheaters are equally likely to have an empty reputation. However, as the game progresses, actors’ strategies are revealed, and cheaters become more likely to be distrusted and therefore more likely to acquire an empty reputation. (This is not an issue with other nonempty reputations, which are each stable predictors of whether an actor will reciprocate.)

To get around this issue, we assume that choosers form posterior beliefs based on the steady-state distribution of actor reputations, which we derive in *SI Appendix*, sections 4.6 and 6.3.

## Equilibrium Analysis

We analyze our model by characterizing its PBEs. Here, we describe the main steps of our demonstration.

### Baseline Equilibrium.

To establish a baseline, we turn off information coming from the institution game by taking $p_{2}=0$. In such a situation, we can restrict reputation to three possibilities: reciprocator, cheater, or empty.

For cooperation to occur with positive probability, we show that reputation must incentivize actors to reciprocate—choosers must trust reputed reciprocators and distrust actors labeled as cheaters. We denote by θ the probability that choosers trust actors with an empty reputation.

Assuming choosers adopt such a strategy, we turn to actors, and consider their continuation payoff in every case—for any type δ, any reputation, and after any action. We show that actors reciprocate if and only if the immediate cost of doing so $c_{1}$ is less than or equal to the delayed benefit of being labeled a reciprocator rather than a cheater, which depends on how much actors discount future payoffs (δ) and how likely they are to be observed ($p_{1}$).

By characterizing reputational incentives more precisely, we show that actors adopt a threshold strategy, whereby, regardless of reputation, they reciprocate if $\delta \geq {\hat{\delta }}^{b}\left(\theta \right)$ and cheat if $\delta <{\hat{\delta }}^{b}\left(\theta \right)$, where:

[B.1] $$\begin{array}{c} {\hat{\delta }}^{b}\left(\theta \right)=\frac{c_{1}}{p_{1}q\left(r-\theta c_{1}\right)}. \end{array}$$

Next, we determine the value of θ. To do so, we calculate the payoff of trusting an actor with empty reputation given that actor reputations have reached their steady state, as a function of θ—we denote this payoff $u^{\infty }\left(\theta \right)$ (recall that we have assumed that choosers form posteriors based on the steady-state distribution of actor reputations).

We show that θ is given by the following algorithm:

[A] $$\begin{array}{cc} \theta^{*} & \equiv \left\{\begin{array}{cc} 0 & \;\text{if}\;u^{\infty }\left(0\right)\leq 0,\\ 1 & \;\text{if}\;u^{\infty }\left(1\right)\geq 0,\\ t & \;\text{such that}\;u^{\infty }\left(t\right)=0. \end{array}\right. \end{array}$$

In other words, θ=0 if trusting actors with an empty reputation leads to a negative or null payoff. Having θ=0 is the best-case scenario for reciprocation. When actors are distrusted by default, good reputation (i.e., being labeled a reciprocator) has more value—in fact, ${\hat{\delta }}^{b}\left(\theta \right)$ is a decreasing function of θ as shown by condition [**B.1**]. Otherwise, if $u^{\infty }\left(0\right)>0$, θ takes a positive value, as it is beneficial to switch to trusting actors with an empty reputation. Specifically, the algorithm yields θ=1 if choosers can afford to trust in this case ($u^{\infty }\left(1\right)\geq 0$), which is the worse case for reciprocation. In all other cases, the algorithm yields a unique value θ∈(0,1).

Finally, we analyze chooser behavior given nonempty reputations. We show that cheaters always exist with positive probability, since discount factors are continuously distributed within (0,1) and ${\hat{\delta }}^{b}\left(\theta \right)>0$. Consequently, it is always beneficial to distrust reputed cheaters, since this label perfectly predicts future cheating (actors follow stationary strategies). Similarly, we show that reciprocators must exist with positive probability, which happens if and only if:

[B.2] $$\begin{array}{cc} {\hat{\delta }}^{b}\left(\theta \right) & <1. \end{array}$$

In fact, this condition defines the domain of existence of the baseline equilibrium, where θ is determined by algorithm (A) and actor strategies depend on the threshold ${\hat{\delta }}^{b}\left(\theta \right)$, as defined by Eq. **B.1**.

### Institution Equilibrium.

We begin by showing that reputation must incentivize both reciprocation and contribution for each to occur with positive probability—choosers must trust reputed reciprocators and contributors, and distrust actors labeled as cheaters or free-riders. We again denote by θ the probability that choosers trust actors with an empty reputation.

Assuming choosers adopt such as strategy, we turn to actors, and consider their continuation payoff in every case. We show that actors contribute under similar conditions as before, balancing the immediate cost of doing so $c_{2}$ against the delayed benefit of being labeled a contributor rather than a cheater, which depends on how much they discount future payoffs (δ) and how likely they are to be observed ($p_{2}$). Institutional incentives influence reciprocating behavior by decreasing the immediate cost of reciprocation to $c_{1}-\left(\beta +\gamma \right)$ and increasing the likelihood of observation to $p_{1}+\pi_{1}$.

By characterizing reputational incentives more precisely, we show that actors adopt a threshold strategy: regardless of reputation, they reciprocate if $\delta \geq {\hat{\delta }}_{1}\left(\theta \right)$ and cheat if $\delta <{\hat{\delta }}_{1}\left(\theta \right)$, and they contribute if $\delta \geq {\hat{\delta }}_{2}\left(\theta \right)$ and free-ride if $\delta <{\hat{\delta }}_{2}\left(\theta \right)$. The thresholds separating reciprocators from cheaters and contributors from free-riders are given by

[I.1] $${\hat{\delta }}_{1}(\theta )=\frac{c_{1}-\beta -\gamma }{\left(p_{1}+\pi_{1})q[r-\gamma -\theta (c_{1}-\gamma -\beta )\right]},$$

[I.2] $${\hat{\delta }}_{2}(\theta )=\frac{c_{2}}{q[p_{2}(r-\gamma )-(p_{1}+\pi_{1})\theta c_{2}]}.$$

Next, we show that θ can be determined using the same algorithm (A) as in the baseline equilibrium. We then similarly show that distrusting reputed cheaters is always beneficial and that we must have ${\hat{\delta }}_{1}\left(\theta \right)<1$, making it beneficial to trust reputed reciprocators.

We conclude by considering reputations acquired in the institution game. Since ${\hat{\delta }}_{2}\left(\theta \right)>0$ and since contribution is assumed to remain easier than reciprocation, it is always beneficial to distrust reputed free-riders—every free-rider will also cheat. For the contributor label, we show that we must have

[I.3] $${\hat{\delta }}_{2}\left(\theta \right)<1,$$

[I.4] $$\mathbb{P}(\delta \geq {\hat{\delta }}_{1}(\theta^{*})|\delta \geq {\hat{\delta }}_{2}(\theta^{*}))\geq \frac{k}{b}.$$

In other words, contribution must occur with positive probability [**I.3**] and be a sufficiently good predictor of future reciprocation, as compared to the ratio of the cost of trust k divided by the benefit of receiving reciprocation b. Condition [**I.4**] ensures that choosers who trust a reputed contributor earn a positive or null payoff. In fact, these conditions define the domain of existence of the institution equilibrium, where θ is determined by algorithm (A) and actor strategies depend on the thresholds ${\hat{\delta }}_{1}\left(\theta \right)$ and ${\hat{\delta }}_{2}\left(\theta \right)$, as defined by Eqs. **I.1** and **I.2**.

## Supplementary Material

Appendix 01 (PDF)

## Data Availability

There are no data underlying this work.
