# Supplementary material for: The social leverage effect: Institutions transform weak reputation effects into strong incentives for cooperation
Source: Proc Natl Acad Sci U S A. 2024 Dec 13;121(51):e2408802121. doi: 10.1073/pnas.2408802121 (PMC11665857; doi:10.1073/pnas.2408802121)
Supplement: Supplementary file 1 — Appendix 01 (PDF) [file pnas.2408802121.sapp.pdf]

Supplementary Information for:

The social leverage effect: Institutions transform weak reputation effects into strong incentives for cooperation

Julien Lie-Panis, Léo Fitouchi, Nicolas Baumard and Jean-Baptiste André
Correspondance: jliep@protonmail.com and leo.fitouchi@gmail.com

Contents

1 Overview of our approach **2**

2 Main elements of the model **2**
2.1 General structure . . . 2
2.2 Stage game . . . 4
2.3 Reputation . . . 5
2.4 Mechanism of the institution . . . 5

3 Technical details of the model **6**
3.1 Histories and strategies . . . 6
3.2 Types, beliefs and objective . . . 8
3.3 Three types of Perfect Bayesian Equilibrium . . . 10

4 Institution equilibrium: actor strategy **13**
4.1 Objective and simplifying notations . . . 13
4.2 Derivation of the reputational benefit R_δ . . . 14
4.3 Threshold discount factor for first-order cooperation . . . 17
4.4 Threshold discount factor for second-order cooperation . . . 18
4.5 Normalized actor payoff . . . 19
4.6 Steady state of actors' reputation . . . 21
4.7 Long-run level of cooperation . . . 24

5 Institution equilibrium: chooser strategy and domain of existence **24**
5.1 Objective . . . 24
5.2 Predictive value of reputation in the steady state . . . 24
5.3 Long-run chooser payoff . . . 27
5.4 Equilibrium value of θ . . . 27
5.5 Domain of existence of the institution equilibrium . . . 29

6 Baseline equilibrium **29**
6.1 Objective . . . 30
6.2 Threshold discount factor for cooperation . . . 30
6.3 Actor payoffs, long-run reputation and level of cooperation . . . 31
6.4 Chooser inferences and long-run payoff . . . 32
6.5 Equilibrium value of θ . . . 34
6.6 Domain of existence . . . 35

7 Implementation into Mathematica 35
7.1 Motivation and general algorithm 35
7.2 Algorithm for the baseline equilibrium 36
7.3 Algorithm for the institution equilibrium 36
7.4 Level of cooperation 37
7.5 Comparison between the monitoring-punishing institution and no institution 39
7.6 Difference in payoffs due to the rewarding, punishing and monitoring institutions 41

List of Figures

S1 Main variables and parameters 3
S2 Stage game 4
S3 Level of cooperation in the baseline equilibrium 37
S4 Level of cooperation for the rewarding, punishing and monitoring institutions 38
S5 Level of cooperation for the monitoring-punishing institution 39
S6 Increase in the level of cooperation due to the monitoring-punishing institution 39
S7 Change in actor and chooser payoff due to the monitoring-punishing institution 40
S8 Change in expected payoff due to the monitoring-punishing institution 41
S9 Change in expected payoff due to the three rewarding, punishing and monitoring institutions 42

1 Overview of our approach

In this supplementary document, we introduce a model of reputation-based first- and second-order cooperation. Second-order cooperation consists in contributing to an institution, that then produces additional incentives for first-order cooperation. First-order cooperation consists in reciprocating a partner's trust. Trust can be conditioned on an individual's reputation, which can create incentives for both first- and second-order cooperation.
We introduce the main elements of our model in section 2, and delve into further technical detail in section 3. We define the **institution equilibrium** of our model in this section, an equilibrium in which some individuals contribute to the institution. We characterize the institution equilibrium in three steps, over sections 3-5. We notably calculate the equilibrium level of first- and second-order cooperation in section 4, and derive the domain of existence in section 5.
In section 6, we establish a **baseline equilibrium** for comparison to the results of the institution equilibrium. Finally, we motivate and explain the computation of a **numerical solution** to our model in section 7.

2 Main elements of the model

In this section, we introduce the main elements of our model, without delving into technical detail (these details are explored in the next section). We explain the general structure of our model (section 2.1), how interactions occur (section 2.2), how we model reputation (section 2.3), and how we model the institution (section 2.4). Table S1 lists the key notations of this document.

2.1 General structure

We consider interactions between two types of players: a large number $n \gg 1$ of actors, and infinitely many choosers. To avoid lengthy repetitions of the terms chooser and actor, we have assigned a gender to each type of player based on the result of a coin toss. Throughout this text, we will use feminine pronouns (she/her) to refer to actors, and masculine pronouns to refer to choosers (he/him/his).

**General parameters**	n	Number of actors	Section 2.1
	δ	Actor discount factor (drawn at birth)	
	μ	Patience of the population (mode discount factor)	Section 7.1
	δ^b	Difficulty of cooperation	Section 6.2
	q	Probability of drawing the trust game	Section 2.2
**Trust game**	T	The chooser trusts the actor	Section 2.2
	$\neg T$	The chooser distrusts the actor	
	k	cost of trust	
	r	Benefit of being trusted	
	C_1	The actor reciprocates, or first-order cooperates	Section 2.2
	D_1	The actor cheats, or first-order defects	
	c_1	Cost of (first-order) cooperation	
	b	Benefit of receiving (first-order) cooperation	
	p_1	Probability that actor's action C_1 or D_1 is observed	
**Institution game**	C_2	The actor contributes, or second-order cooperates	Section 2.2
	D_2	The actor free-rides, or second-order defects	
	c_2	Cost of second-order cooperation	
	p_2	Probability that actor's action C_2 or D_2 is observed	
**Effect of the institution**	β	Reward for cooperators	Section 2.4
	γ	Penalty for defectors	
	π_1	Increase in the probability of observation	
	ρ	Efficiency of the institution	
	$\mathbf{r_C}$	Net payoff of cooperators, $\mathbf{r_C} = r - c_1 + \beta$	Section 4.1.4
	$\mathbf{r_D}$	Net payoff of defectors, $\mathbf{r_D} = r - \gamma$	
	$\mathbf{c_1}$	Net cost of cooperation, $\mathbf{c_1} = \mathbf{r_D} - \mathbf{r_C}$	
	$\mathbf{p_1}$	Net probability of observation, $\mathbf{p_1} = p_1 + \pi_1$	

Figure S1: This table lists the key variables and parameters introduced in this supplementary material.

Our model is composed of n infinitely repeated games—one for each actor. These repeated games occur in parallel and in discrete time, in rounds indexed by the letter $t \in \mathbb{N}$. We refer to all these interactions as the repeated game, or simply the game, and to the interactions that only concern a specific actor as the actor's repeated game. In every round, n choosers are drawn from the infinite chooser population, and assigned to a different actor. At the end of the round, the n actors move on to the next round of their repeated game, while the n choosers for that round exit the game. In other words, actors are long-lived, and play all rounds of their repeated game, while choosers are short-lived, and play only one round of interaction (if and when they are assigned to an actor). We complete the description of our game in section 2.2, by describing the stage game of a generic actor.

Choosers decide whether or not to trust the actor they are assigned to, based on limited information—her reputation, as defined in section 2.3. Their role is to motivate cooperation by actors.

Actors face two types of interactions, and two types of cooperative decisions. Sometimes they can pay to reciprocate a chooser's trust—first-order cooperation—and sometimes they can pay to contribute to an institution, whose purpose is to incentivize reciprocation by every actor—second-order cooperation. The functioning of the institution, which depends on individual contributions, is explained in section 2.4.

To capture the fact that individuals will not all equally be motivated to cooperate, we assume that actors have varying time preferences. Every actor is characterized by a discount factor, which is drawn before the onset of the game, according to a distribution μ_0. μ_0 is a probability density function defined over the entire interval of possible discount factors $\Delta \equiv (0, 1)$. Any value between 0 and 1 is possible: we assume that μ_0 is of full support, i.e., that: $\mu_0(\delta) > 0, \forall \delta \in \Delta$.

The value of a given actor's discount factor, which we denote using the symbol δ, is hidden to other players. Throughout their repeated game, actors discount future payoffs according to this value. The lifetime payoff of an actor of discount factor δ is equal to the payoff earned in the initial round ($t = 0$), plus δ times the payoff earned in the next round ($t = 1$), plus δ^2 times the payoffs earned in the round after that ($t = 2$), and so on. In section 4, we show that, in our main equilibrium, sufficiently patient actors (sufficiently high δ) engage in both forms of cooperation, while sufficiently impatient actors engage in neither form of cooperation. Actors of intermediate patience only engage in the cheapest form of cooperation.

2.2 Stage game

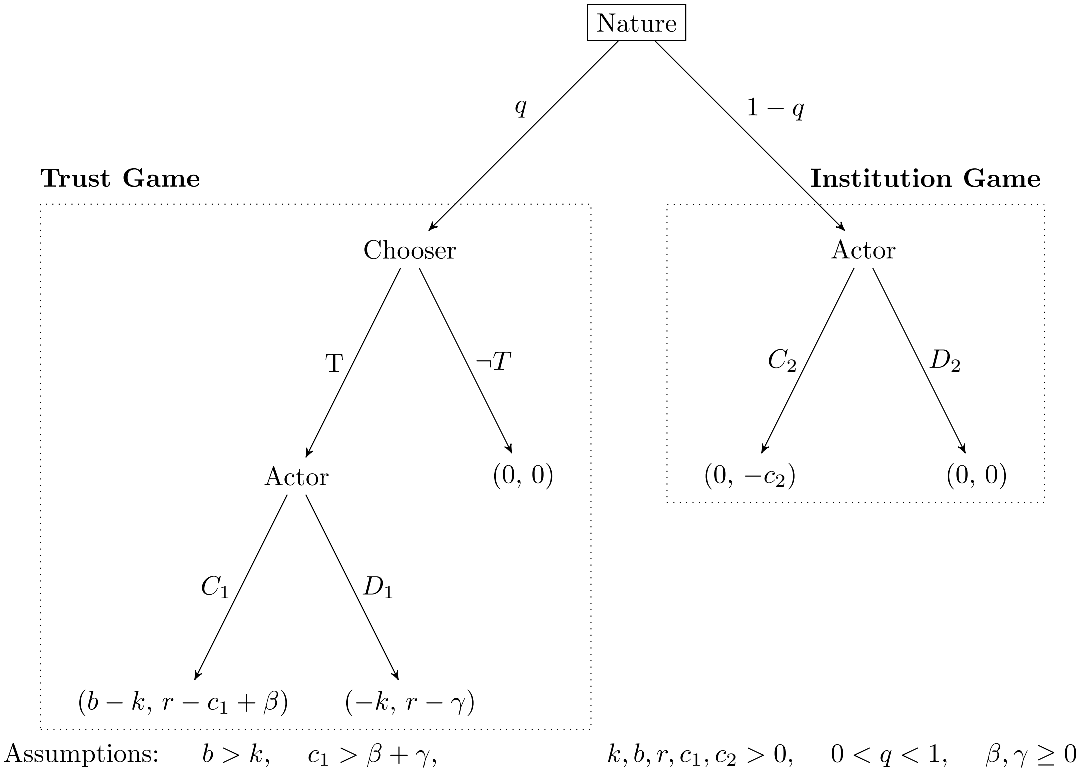

Figure S2: Stage game. Nature begins by setting the interaction type: a trust game is drawn with probability q, and the institution game is drawn with probability $1 - q$. In the case of a **trust game** (left branch), the actor play an asymmetric prisoner's dilemma with the chooser she has been assigned this round. We assume $b > k$ and $c_1 > \beta + \gamma$ to keep the structure of an asymmetric prisoner's dilemma. For the chooser, playing T instead of $\neg T$ is net beneficial if the actor subsequently plays C_1, but net costly if the actor subsequently plays D_1. For the actor, playing C_1 instead of D_1 is always net costly, despite the effect of the institution. The institution is materialized here by a reward $\beta \geq 0$ granted in the case that the actor plays C_1, and a penalty $\gamma \geq 0$ inflicted in the case that the actor plays D_1. In the **institution game** (right branch), the actor decides whether or not to contribute to the institution by paying $c_2 > 0$. This decision is made in parallel with every other actor who has drawn the institution game that round (we detail the functioning of the institution in section 2.4).

In this section, we consider a generic actor, without specifying the value of her discount factor. We describe the stage game of this generic actor's infinitely repeated game. This stage game is illustrated by Figure S2. As mentioned above, it is infinitely repeated, for each of the rounds $t \in \mathbb{N}$.

Each round proceeds as follows. First, nature draws between two types of interaction: a trust game with probability q, and the institution game with probability $1 - q$. Then, the actor and the chooser she has been assigned that round play according to the rules of the interaction at hand.

If a trust game is drawn, both players play an asymmetric prisoner's dilemma with two steps. In the first step, the chooser decides whether to trust (i.e. play action T) or distrust ($\neg T$) the actor, putting an early end to the

interaction. If he trusts her, the actor then decides whether to reciprocate (C_1) that trust, or cheat (D_1), in the second step. We refer to reciprocation as first-order cooperation, and to cheating as first-order defection—or simply cooperation and defection when there is no ambiguity—which is why we use the labels C_1 and D_1 to designate these two actor actions.[1]

Trust costs $k > 0$ to the chooser, and brings benefit $r > 0$ to the actor. First-order cooperation (reciprocation) costs $c_1 > 0$ to the actor, and brings benefit $b > 0$ to the chooser. We assume $b > k$: the chooser benefits from trusting the actor if she subsequently reciprocates that trust.

In the institution game, the actor plays without the chooser. She can either contribute (C_2) to an institution, whose functioning is described in section 2.4, or free-ride (D_2). We refer to contribution as second-order cooperation, and to free-riding as second-order defection, which is why we use the labels C_2 and D_2 to designate these other two actor actions. Second-order cooperation (contribution) costs $c_2 > 0$ to the actor.

2.3 Reputation

In this section, we continue to consider a generic actor. Throughout her repeated game, the actor faces infinitely many different short-lived choosers, who interact with her with probability q in the relevant round. In any given round, we refer to an actor's current co-player as the current chooser (or simply the chooser, when there is no ambiguity), and to her co-player in the next round as the next chooser.

We restrict the information available to choosers in the following manner. At the end of each round, we assume that the next chooser observes the actor's action in that round with baseline probability p_1 if the actor faced the trust game and was trusted by the current chooser (this probability can be increased through the effect of the institution; see section 2.4), and with fixed probability p_2 if the actor faced the institution game ($0 < p_1 < 1$, $0 < p_2 < 1$). We assume that choosers do not observe the actor's behavior in rounds before the one that just ended, and do not observe the behavior of previous choosers.

What this means is that in any round $t \geq 0$, when the current chooser faces the trust game, and therefore the option to trust or distrust the actor, he can be in one of five situations. If the actor did not play in round $t-1$ (because $t = 0$, or because in round $t-1$ she faced the trust game and was distrusted) or if her action was not observed, the chooser does not have access to any information. Otherwise, the chooser has access to one piece of information, pertaining to the actor's action in round $t-1$.

We refer to these five cases as the actor's **reputation**, or, interchangeably, as the information available to the (current) chooser. (Note that the actor's reputation is defined with respect to her current partner.) Let $\mathcal{R} \equiv \{\emptyset, \mathcal{C}_1, \mathcal{D}_1, \mathcal{C}_2, \mathcal{D}_2\}$ be the set of possible actor reputations, $\emptyset$ referring to the case of an empty reputation (i.e., the case when the chooser in a given round has no information), $\mathcal{C}_1$ referring to the case when the chooser has observed the actor playing C_1 in the previous round, and so on. Let $\mathcal{R}^* \equiv \mathcal{R} \setminus \{\emptyset\}$ be the set of non-empty reputations.

2.4 Mechanism of the institution

In this section, we consider the entire actor population, and the entire repeated game. In any given round of the entire game, in expectation, $(1-q) \times n$ actors play the institution game, in which they can decide to contribute to the same institution, while $q \times n$ actors play separate trust games, in which they can decide to cooperate with different choosers, if trusted. Since the number of actors is large ($n \gg 1$), we equate the actual number of actors in either game with their expected value.

Let n_2 be the number of actors who would contribute to the institution in this round if given the chance (we show below that n_2 does not depend on the round, or even the game history, which is defined in section 3; we also properly define actors' strategy in that section). Each contributions is worth c_2. Since individuals are assigned to the institution game independently of their behavior, the total amount of contributions received by the institution is equal to:

$$\text{Contributions Received} \equiv (1-q) \times n_2 \times c_2.$$

The institution transforms these contributions into incentives for first-order cooperation. These contributions are multiplied by a factor $\rho > 0$ measuring the efficiency of the institution. For every unit contributed to the institution, ρ

[1] In the main text, we use the term 'cooperation' exclusively to describe a successful interaction between a chooser and an actor, where the chooser trusts and the actor reciprocates. We represent the rate of such cooperation in our numerical solution. In contrast, in the mathematical description, we stick to the term reciprocation when referring to actor actions.
Here, however, we use the terms 'cooperation' and 'first-order cooperation' interchangeably with the term 'reciprocation', so that we can use the notation C_1 for that action. We also use the term 'defection' interchangeably with 'cheating', and denote that action D_1. This applies throughout the document, in particular in sections 2-6, where we analyze the model mathematically. In section 4.7, we introduce the term '(long-run) level of cooperation' to refer to the rate of successful chooser-actor encounters, which we use in section 7, where we compute our numerical solution, and plot figures like the ones presented in the main text.

units are created to incentivize first-order cooperation. The total amount of of incentives generated by the institution is equal to:

$$\text{Incentives Generated} \equiv \rho \times \text{Contributions Received}.$$

We assume that these incentives are equally applied to each of the $q \times n$ separate trust games that are played in that same round. One portion is allocated to rewarding cooperators, another portion is used for punishing cheaters, and the remaining portion is dedicated to monitoring. Every actor who plays C_1 earns a reward $\beta \geq 0$, ever actor who plays D_1 suffers a penalty $\gamma \geq 0$, and the probability of observation in every trust game increases by $\pi_1 \geq 0$. We apply a factor of conversion equal to c_1, the baseline cost of cooperation, to convert the increase in probability of observation π_1 into an amount in resource units $c_1\pi_1$. Summing over every trust game, the total incentives generated by the institution are also equal to:

$$\text{Incentives Generated} = q \times n \times (\beta + \gamma + c_1\pi_1).$$

Replacing, and noting $f_2 = n_2/n$ the proportion of would-be contributors in our large actor population, we deduce:

$$\rho = \frac{\text{Incentives Generated}}{\text{Contributions Received}} = \frac{q(\beta + \gamma + c_1\pi_1)}{(1-q)f_2c_2}. \tag{2.1}$$

Note that we use Greek letters to refer to the institution and the incentives it creates throughout the model. β, γ and π_1 are left unspecified: with this general model, we can consider different types of institutions. For instance, a (purely) punishing institution is obtained by taking $\beta = \pi_1 = 0$; in that case, the total contribution is entirely allocated to punishing defectors, who are inflicted a penalty of $\gamma = \rho f_2 c_2(1-q)/q$. A (purely) monitoring institution is obtained by taking $\beta = \gamma = 0$; in that case, the probability of observation in the trust game increases by $\pi_1 = \rho f_2(c_2/c_1)(1-q)/q$.

Accounting for the effect of the institution in a given round, the net cost of cooperation is equal to the total payoff of defectors minus the total payoff of cooperators, that is: $(r-\gamma) - (r - c_1 + \beta) = c_1 - (\beta + \gamma)$. We assume that, even after accounting for the effect of the institution, cooperation remains costly for actors, that is:

$$c_1 - (\beta + \gamma) > 0. \tag{2.2}$$

In addition, we assume that the likelihood of observation in the trust game remains below 1, i.e. that: $p_1 + \pi_1 < 1$.

3 Technical details of the model

In this section, we go into further technical detail. We define histories and the strategy space (section 3.1), and introduce the concept of a Perfect Bayesian Equilibrium (section 3.2). Importantly, we assume that choosers behave as if many rounds of the game have already been played, and introduce their long-run payoffs (as well as their long-run posterior beliefs). We define the institution equilibrium in section 3.3.

3.1 Histories and strategies

Throughout this section, we consider a generic actor, as well as her infinitely repeated game, and those choosers that are assigned to her repeated game, i.e., that are assigned to this actor in a given round t. Since choosers only observe the behavior of the actor they are assigned to, we define the histories of that repeated game based only on the reputation of that actor. Similarly, we define chooser and actor strategy, their payoffs, and the continuation game and strategies without reference to the other repeated games played by other actors. To do so, we rely on the framework developed by Mailath and Samuelon (1), which applies to a repeated game played between a long-lived player and infinitely many short-lived players, and adopt their notations when possible.

The general structure of our model, with n repeated games being played in parallel and $(1-q)n_2c_2$ contributions being transformed into incentives for cooperation by an institution, affects the value of players' payoffs and their equilibrium strategies, but not our ability to define payoffs and strategies in isolation. The fact that there is incomplete information about the actor's discount factor pushes us to introduce a Bayesian framework in section 3.2 below; here, throughout section 3.1, this issue can be ignored.

3.1.1 Chooser history equivalence classes and strategy space

Because we strongly restrict the information available to choosers, chooser histories of this repeated game can be divided in five equivalence classes, depending on the actor's reputation in the eyes of the (current) chooser. We denote by $\mathcal{H}_{ch} \mid R$ the equivalence class attained when the actor's reputation is $R \in \mathcal{R}$, and denote by $\mathcal{H}_{ch} \mid \mathcal{R}$ the set comprised of the five equivalence classes for histories of the repeated game; the set of chooser histories $\mathcal{H}_{ch}$ is the union of those equivalence classes.

For simplicity, we equate $\mathcal{R}$ with $\mathcal{H}_{ch} \mid \mathcal{R}$. That is, we define a chooser's strategy directly as a function of the reputation of the actor he is assigned to, rather than as a function of the history equivalence class of that actor's repeated game. A pure strategy for a chooser specifies whether to trust or distrust the actor depending on her reputation; it is a map:

$$\sigma_{ch} : \mathcal{R} \to \{T, \neg T\}.$$

We restrict to the set of chooser strategies $\mathcal{S}_{ch}$ which is pure for non-empty reputations, i.e. the set of strategies following which the chooser plays either T or $\neg T$ with certainty given any information $R \in \mathcal{R}^*$. We denote by $\sigma^*_{ch} \equiv \sigma_{ch} \mid_{\mathcal{R}^*}$ the restriction of a chooser's strategy to the non-empty information set. There are $2^4 = 16$ possible values for σ^*_{ch}, and an infinite number of possible chooser strategies since we allow choosers to mix between T and $\neg T$ given $\emptyset$. When choosers play according to a strategy $\sigma_{ch} \in \mathcal{S}_{ch}$, we denote by θ the probability that they trust given $\emptyset$; a chooser's strategy is completely described by σ^*_{ch} and $\theta \in [0, 1]$.

We have allowed choosers to mix given empty reputation. We return to this issue in section 5.4, in which we calculate the value of θ in equilibrium. Our calculation shows that restricting to pure strategies would lead us not to consider certain cases—under certain parameter conditions, choosers benefit from deviation to trusting given $\theta = 0$ and from deviation to trusting given $\theta = 1$ (because the value of θ influences actor equilibrium strategy).

3.1.2 Actor history equivalence classes and strategy space

The actor does not always have an opportunity to act. In each round, there are three possibilities: either the trust game is drawn and the current chooser plays T, in which case the actor has an opportunity to play C_1 or play D_1; or the trust game is drawn and the chooser plays $\neg T$, in which case the actor does not play this round; or the institution game is drawn, in which case the actor has an opportunity to play C_2 or play D_2. Let $\mathcal{T}$, $\neg\mathcal{T}$ and $\mathcal{I}$ be the corresponding events, in the above order.

We restrict actor histories in accordance to the restriction applied to chooser histories. Relevant actor histories can be grouped into 10 classes, 5 classes of the form $(R, \mathcal{T})$, in which the actor plays in the trust game after have been trusted by the chooser, and 5 classes of the form $(R, \mathcal{I})$, in which the actor plays in the institution game—R being any element of the set of reputations $\mathcal{R}$. In other words, relevant actor histories are elements of the set $\mathcal{R} \times \{\mathcal{T}\}$, and elements of the set $\mathcal{R} \times \{\mathcal{I}\}$.

A pure actor strategy $\sigma_{ac} \equiv (\sigma_{ac,1}, \sigma_{ac,2})$ is comprised of two maps. The first map deals with first-order cooperation; it specifies whether to reciprocate or cheat after being trusted in the trust game, as a function of an actor's (current) reputation and her (fixed) discount factor:

$$\sigma_{ac,1} : \mathcal{R} \times \Delta \to \{C_1, D_1\}.$$

The second deals with second-order cooperation; it specifies whether to contribute or free-ride in the institution game, again as a function of an actor's (current) reputation and her (fixed) discount factor:

$$\sigma_{ac,2} : \mathcal{R} \times \Delta \to \{C_2, D_2\}.$$

Note that for the sake of economy of notations, we omit the events $\mathcal{T}$ and $\mathcal{I}$ from $\sigma_{ac,1}$ and $\sigma_{ac,2}$, respectively. We restrict to the set $\mathcal{S}_{ac}$ of pure actor strategies.

3.1.3 Continuation strategy profile

For any $R \in \mathcal{R}$, the continuation game associated with R is defined as the infinitely repeated game in which the chooser initially has information R, corresponding to the actor's initial reputation. The continuation game associated with R occurs each time the actor attains reputation R at the end of the previous round.

In the continuation game associated with R, the chooser plays directly after. For any strategy profile σ, we denote by $\sigma \mid_R$ the continuation strategy profile induced by R.

The actor plays after histories of the form $(R, \mathcal{T})$ and $(R, \mathcal{I})$. For any strategy profile σ, and every $(R, \mathcal{X}) \in \mathcal{R} \times \{\mathcal{T}, \mathcal{I}\}$, we denote by $\sigma \mid_{R,\mathcal{X}}$ the continuation strategy profile induced by $(R, \mathcal{X})$.

3.1.4 Chooser payoffs

For any strategy profile σ, reputation R, and round t, let $u(\sigma \mid_R)$ be the expected payoff of a chooser in the continuation game. This is the payoff that the chooser for round t can expect to gain given that the trust game is drawn, when players play according to σ, and that the chooser has information R on the actor. A chooser's payoff given a piece of information R depend on her posterior beliefs concerning the actor's discount factor given that R is true (this is implicit in the above notation)—we return to this issue, and define these posterior beliefs, below, in section 3.2.

3.1.5 Actor continuation payoff

The actor earns payoffs throughout the game. For any δ, σ and R, let $U_\delta(\sigma \mid_R)$ be the lifetime expected payoff of the actor starting from the continuation game associated with R. Since the actor begins with empty reputation, $U_\delta(\sigma) \equiv U_\delta(\sigma \mid_\emptyset)$ is the actor's expected payoff over the entire game.

In addition, we define two other classes of continuation payoffs for the actor, relevant to the histories after which she actually plays, in the trust and institution game respectively. For any δ, σ and R, let $U_\delta(\sigma \mid_{R,\mathcal{T}})$ be the lifetime expected payoff of the actor given history $(R, \mathcal{T})$, and $U_\delta(\sigma \mid_{R,\mathcal{I}})$ be the lifetime expected payoff of the actor given history $(R, \mathcal{I})$. These correspond to the lifetime's payoff of the actor in the continuation game associated with R, once either event $\mathcal{T}$ or event $\mathcal{I}$ has occurred (hence not comprising the benefit of being trusted by choosers in the first case).

3.2 Types, beliefs and objective

3.2.1 Equilibrium concept and objective

Here, we return to the fact that there is incomplete information about the generic actor's time preferences. In the vocabulary of Bayesian Games (see e.g., 2), the value of the actor's discount factor, her δ, constitutes her hidden type. This type is hidden to choosers but is crucial for them, since actors can cooperate and contribute depending on their δ (and in fact cooperate and contribute depending only on their δ in the equilibria of interest, as we will see).

An actor's δ is drawn at birth according to the probability density function μ_0. We assume that μ_0 is common knowledge among all players of the generic repeated game under consideration: in the vocabulary of Bayesian Games, μ_0 captures the prior beliefs of choosers about the relevant actor's type δ. For any piece of information $R \in \mathcal{R}$, we denote by $\mu(. \mid R)$ the posterior beliefs of choosers about the relevant actor's type—like μ_0, $\mu(. \mid R)$ is a probability density function defined over the set of possible discount factor values Δ.

In general, in a multi-stage model with incomplete information, a pair (σ, μ), comprising a strategy profile $\sigma = (\sigma_{ch}, \sigma_{ac})$ and posterior beliefs μ is a Perfect Bayesian Equilibrium (PBE) if for any history (even an unrealized history): (B) Bayes' rule is used to update choosers' beliefs whenever possible (P) there are no beneficial deviations available to choosers given their beliefs, or to any type of actor.

Objective: Our goal is to characterize every pair (σ, μ) such that:

(i) the strategy profile $\sigma = (\sigma_{ch}, \sigma_{ac})$ belongs to the set $\mathcal{S} \equiv \mathcal{S}_{ch} \times \mathcal{S}_{ac}$, and

(ii) (σ, μ) is a Perfect Bayesian Equilibria of the repeated game.

3.2.2 A comment concerning perfection (P)

Given our assumptions restricting player histories, the perfection criterion (P) can be written as (recall that chooser payoffs given a reputation R depend on their posterior beliefs $\mu(. \mid R)$):

$$\begin{aligned}
&\forall \sigma'_{ch} \in \mathcal{S}_{ch}, \forall R \in \mathcal{R}, && u(\sigma \mid_R) \geq u((\sigma'_{ch}, \sigma_{ac}) \mid_R),\\
&\forall \sigma'_{ac} \in \mathcal{S}_{ac}, \forall R \in \mathcal{R}, \forall \delta \in \Delta, && U_\delta(\sigma \mid_{R,\mathcal{T}}) \geq U_\delta((\sigma_{ch}, \sigma'_{ac}) \mid_{R,\mathcal{T}}),\\
& && U_\delta(\sigma \mid_{R,\mathcal{I}}) \geq U_\delta((\sigma_{ch}, \sigma'_{ac}) \mid_{R,\mathcal{I}}).
\end{aligned}$$

Perfection thus pushes us to consider best responses for either player even given unrealized histories, e.g., even given that an actor of type δ is trusted despite holding uncooperative reputation $\mathcal{D}_1$ (which we show below does not

occur along the outcome path of that type of actor). By restricting our analysis to perfect equilibria, we restrict to strategy profiles that are stable to rare mistakes by players (e.g., a chooser mistakenly trusting an actor of uncooperative reputation) (see 3, for a thorough analysis in games without hidden types). Arguably, perfection is relevant when one is interested in endpoints of an evolutionary process—mistakes or misunderstandings can happen.

For simplicity of notations, we will further assume that players play the prosocial action when indifferent between two options; that is, that an actor of type δ plays C_1 given a history $(R, \mathcal{T})$ if indifferent between C_1 and D_1, C_2 if indifferent between C_2 and D_2, and that the chooser plays T if indifferent between T and $\neg T$ (we show below that such indifference happens for specific real values of our parameters, which they take with probability 0).

3.2.3 An important assumption concerning Bayesian inferences (B)

The Bayesian inference criterion (B) imposes to use Bayes' rule to form posteriors whenever possible (there are other restrictions, listed in 2, pp. 331-3, which are already met here). In the context of our model, in which each chooser enters the game with exactly one piece of information R, we take this to mean that choosers must always use Bayes' rule to form posteriors given the empty reputation $\emptyset$ (since $p_1 + \pi_1 < 1$ and $p_2 < 1$, there is always a positive chance that an actor has an empty reputation, whatever players' strategies), and must also use Bayes' rule to form posteriors for any non-empty reputation for which there exists a non-negligible subset of types that would play the corresponding action if given the opportunity (e.g., for $R = \mathcal{C}_1$, if there exists a reputation R' and a subset $I \subset \Delta$ of probability $\mathbb{P}(\delta \in I) = \int_I \mu_0 > 0$ such that any actor with $\delta \in I$ and reputation R' cooperates if trusted).

In practice, there will be three possibilities, as detailed below (section 3.3). In situations where every type of actor never cooperates and never contributes, whatever the history, (B) constrains chooser posteriors given uncooperative reputations $\mathcal{D}_1$ and $\mathcal{D}_2$, but does not constrain their posteriors given cooperative reputations. We can then technically set any posterior we want given $\mathcal{C}_1$ or $\mathcal{C}_2$, meaning that choosers can make any arbitrary inferences about the δ of an actor whose reputation is null. This permitted arbitrariness has little bearing on the final result, since following the perfection criterion, choosers will distrust even given $\mathcal{C}_1$ or $\mathcal{C}_2$—whatever their beliefs about an actor's type, trust is strictly costly since every type of actor will defect. The other two situations are the one in which every type of actor never contributes but certain types cooperate, and the situation in which certain types engage in both forms of cooperation.

This leaves an important question unresolved: how should Bayesian inference be implemented? When (B) applies, a reputation can be attained by infinitely many different histories: how should we weigh different elements of the history equivalence class, and compared to what?

To solve this question, we assume that choosers form posterior beliefs given that every actor's reputation has attained its steady state. As we will see in more detail, along her outcome path, the reputation of any actor type follows a Markov process, which converges to a probabilistic steady state, depending on (her type and therefore on) her actions. In our two equilibria of interest, in sections 4 and 6, we will derive the long-run probability distribution of actors' reputation as a function of the distribution of their time preferences μ_0. The probability of a reputation R taken over the entire long-run distribution is denoted by $\mathbb{P}^\infty(R)$. We will then use this distribution to derive chooser posteriors, whenever possible.

Another way of stating this assumption, is to say that we consider equilibria in which choosers' behave as if a large number of rounds of the game have already been played, based on the corresponding beliefs. To be clear about this, we introduce the **chooser long-run payoffs**, which, for any σ and R, we denote by $u^\infty(\sigma \mid_R)$. This is the expected payoff of a chooser given that he faces an actor of reputation R, assuming that every actor's reputation can be approximated using the steady state of their Markov chain. In addition, we denote chooser posteriors by $\mu^\infty(. \mid R)$ from here on.

Given that a chooser has these posteriors and faces an actor of reputation R, we denote the subjective probability that this actor will cooperate if trusted by the chooser by:

$$\mathbb{P}_{ch}(C_1 \mid R) \equiv \mathbb{P}(\sigma_{ac,1}(R, \delta) = C_1 \mid R, \delta \sim \mu^\infty(. \mid R)).$$

When a reputation R occurs with positive probability in the steady state ($\mathbb{P}^\infty(R) > 0$), the objective probability that an actor of reputation R cooperates if trusted in the long-run steady state (i.e., using the long-run distribution of reputations derived in section 4) is denoted by $\mathbb{P}^\infty(C_1 \mid R)$. When the Bayesian inference criterion applies and $\mathbb{P}^\infty(R) > 0$, we have:

$$\mathbb{P}_{ch}(C_1 \mid R) = \mathbb{P}^\infty(C_1 \mid R).$$

As we will see, relying on the steady state of actors' reputation to form chooser posteriors will be useful for the empty reputation $\emptyset$. The informative value of the empty reputation is indeed a function of the history. In particular, it is a function of time: along the outcome path, $\emptyset$ is non-informative at $t = 0$ (since every actor starts out with

empty reputation), but $\emptyset$ indirectly informs about actor actions for $t > 0$ (every actor that isn't trusted in round $t-1$ is certain to acquire empty reputation—therefore, an uncooperative actor is more likely to acquire empty reputation than a cooperative actor).

In contrast, our concern is largely moot for non-empty reputations. Intuitively, since actor strategy is stationary (Lemma 3.4 below), every non-empty reputation gives information about the actor's discount factor δ, and therefore her future actions. Since $\mathcal{C}_1$ and $\mathcal{D}_1$ perfectly indicate whether an actor cooperated or defected in the past, in both equilibria of interest, it always pays to trust given $\mathcal{C}_1$ and distrust given $\mathcal{D}_1$. In addition, since the actor's access to the institution game is not constrained by her past reputation (she does not need to be 'trusted' by the institution to free-ride or contribute), $\mathcal{C}_2$ and $\mathcal{D}_2$ always give the same information about her δ, and are thus stationary predictors of whether she will cooperate or defect if trusted.

3.3 Three types of Perfect Bayesian Equilibrium

3.3.1 Condition for trust

We begin by deriving a general condition for trust.

Lemma 3.1: Condition for inferring trust

$\forall \sigma = (\sigma_{ch}, \sigma_{ac}) \in \mathcal{S}, \forall \mu^\infty,$ such that (σ, μ^∞) is a PBE, $\forall R \in \mathcal{R}$,

$$\sigma_{ch}(R) = T \iff \mathbb{P}_{ch}(C_1 \mid R) \geq \frac{k}{b}. \tag{3.1}$$

In a PBE, choosers trust given sufficiently good predictors of the actor's cooperation, and distrust given sufficiently bad predictors of the actor's cooperation.

Proof. Let us consider a PBE (σ, μ^∞), with $\sigma = (\sigma_{ch}, \sigma_{ac})$, and a certain reputation $R \in \mathcal{R}$. Given R, choosers form the posterior $\mu^\infty(. \mid R)$. This posterior is either arbitrary, if actors never perform the corresponding action when given the chance, or formed via Bayesian inference based on the steady state of the game, as detailed in section 5.

If choosers trust an actor of reputation R, they pay k with certainty, and earn back b if the actor reciprocates. The subjective probability that this actor reciprocates depends on her strategy σ_{ac}, her current reputation, which is R and commonly known, and her δ, which is hidden, but over which choosers hold beliefs $\mu^\infty(. \mid R)$. This is the probability $\mathbb{P}_{ch}(C_1 \mid R)$ introduced just above.

In contrast, choosers that distrust given R gain 0 with certainty. In a PBE, choosers will then trust given R if and only if:

$$-k + \mathbb{P}_{ch}(C_1 \mid R) \times b \geq 0,$$

which, re-arranging, is equivalent to the proposed conditon. □

3.3.2 Conditions for cooperation

We derive two general conditions for first- and second-order cooperation, respectively.

Lemma 3.2: Condition for first-order cooperation

$\forall \sigma = (\sigma_{ch}, \sigma_{ac,1}, \sigma_{ac,2}) \in \mathcal{S}, \forall \mu^\infty,$ such that (σ, μ^∞) is a PBE, $\forall R \in \mathcal{R}, \forall \delta \in \Delta$,

$$\sigma_{ac,1}(R, \delta) = C_1 \iff c_1 - (\beta + \gamma) \leq \delta \times (p_1 + \pi_1) \times (U_\delta(\sigma \mid_{\mathcal{C}_1}) - U_\delta(\sigma \mid_{\mathcal{D}_1})). \tag{3.2}$$

In a PBE, whatever her current reputation R, an actor of discount factor δ cooperates when given the opportunity to do so if and only if the immediate, net cost of cooperation $c_1 - \gamma - \beta$ is smaller than her future (i.e., multiplied by her δ) benefit of achieving reputation $\mathcal{C}_1$ rather than $\mathcal{D}_1$, if observed (with probability $p_1 + \pi_1$).

Proof. Let us consider a PBE (σ, μ^∞), with $\sigma = (\sigma_{ch}, \sigma_{ac})$, and $\sigma_{ac} = (\sigma_{ac,1}, \sigma_{ac,2})$. Given any history of the form $(R, \mathcal{T})$, σ_{ac} will prescribe playing C_1 if and only if her continuation payoff were she to play C_1 is greater or equal than her continuation payoff were she to play D_1 (recall that we assumed that actors play C_1 if indifferent between both options).

If the actor cheats, she gains $r-\gamma$ in the current round. Her reputation starting in the next round is then $\mathcal{D}_1$ with probability $p_1+\pi_1$, and $\emptyset$ with probability $1-(p_1+\pi_1)$. If she reciprocates, she gains only $r-c_1+\beta<r-\gamma$, and achieves future reputation $\mathcal{C}_1$ with probability $p_1+\pi_1$, and, again, $\emptyset$ with probability $1-(p_1+\pi_1)$. Following the actor's action, the continuation game associated with $\mathcal{C}_1$, $\mathcal{D}_1$ or $\emptyset$ occurs, depending on the actor's chosen action and the outcome of observation.

Given $(R,\mathcal{T})$, σ_{ac} will then prescribe playing C_1 if and only if:

$$r-\gamma+\delta[(p_1+\pi_1)U_\delta(\sigma\mid_{\mathcal{D}_1})+(1-p_1+\pi_1)U_\delta(\sigma\mid_\emptyset)] \\ \leq r-c_1+\beta+\delta[(p_1+\pi_1)U_\delta(\sigma\mid_{\mathcal{C}_1})+(1-p_1+\pi_1)U_\delta(\sigma\mid_\emptyset)].$$

Simplifying, this is equivalent to:

$$r-\gamma+\delta\times(p_1+\pi_1)\times U_\delta(\sigma\mid_{\mathcal{D}_1})\leq r-c_1+\beta+\delta\times(p_1+\pi_1)\times U_\delta(\sigma\mid_{\mathcal{C}_1}).$$

Re-arranging, we obtain the proposed inequality:

$$c_1-(\beta+\gamma)\leq\delta\times(p_1+\pi_1)\times(U_\delta(\sigma\mid_{\mathcal{C}_1})-U_\delta(\sigma\mid_{\mathcal{D}_1})). \tag{3.2}$$

□

Lemma 3.3: Condition for second-order cooperation

$\forall\sigma=(\sigma_{ch},\sigma_{ac,1},\sigma_{ac,2})\in\mathcal{S},\forall\mu^\infty,$ such that (σ,μ^∞) is a PBE, $\forall R\in\mathcal{R},\forall\delta\in\Delta,$

$$\sigma_{ac,2}(R,\delta)=C_2\iff c_2\leq\delta\times p_2\times(U_\delta(\sigma\mid_{\mathcal{C}_2})-U_\delta(\sigma\mid_{\mathcal{D}_2})). \tag{3.3}$$

In a PBE, whatever her current reputation R, an actor of discount factor δ contributes when given the opportunity to do so if and only if the immediate cost of second-order cooperation c_2 is smaller than her future (i.e., multiplied by her δ) benefit of achieving reputation $\mathcal{C}_2$ rather than $\mathcal{D}_2$, if observed (with probability p_2).

Proof. The proof is analogous to the one above. For any PBE (σ,μ^∞), with $\sigma=(\sigma_{ch},\sigma_{ac})$ and $\sigma_{ac}=(\sigma_{ac,1},\sigma_{ac,2})$, given any history of the form $(R,\mathcal{I})$, the actor gains 0 if she plays D_2 in the current round, following which she attains reputation $\mathcal{D}_2$ with probability p_2. If she plays C_2, she pays c_2, but instead attains reputation $\mathcal{C}_2$ with probability p_2. Whatever her action, she attains empty reputation with probability $1-p_2$.

Comparing the continuation payoffs in both cases (similarly to before, $\delta\times(1-p_2)\times U_\delta(\sigma\mid_\emptyset)$ is a factor in both continuation payoffs, and cancels out), we deduce that σ_{ac} will prescribe playing C_2 if and only if:

$$0+\delta\times p_2\times U_\delta(\sigma\mid_{\mathcal{D}_2})\leq -c_2+\delta\times p_2\times U_\delta(\sigma\mid_{\mathcal{C}_2}).$$

Re-arranging, we obtain the proposed inequality. □

3.3.3 Actor strategy is stationary

Using Lemmas (3.2-3.3), we deduce that the strategy of actors must be stationary.

Lemma 3.4: Actor strategy is stationary

$\forall\sigma=(\sigma_{ch},\sigma_{ac,1},\sigma_{ac,2})\in\mathcal{S},\forall\mu^\infty,$ such that (σ,μ^∞) is a PBE, $\forall(R,R')\in\mathcal{R}^2,\forall\delta\in\Delta,$

$$\sigma_{ac,1}(R,\delta)=\sigma_{ac,1}(R',\delta),$$
$$\sigma_{ac,2}(R,\delta)=\sigma_{ac,2}(R',\delta).$$

In a PBE, the strategy of actors is stationary—actors cooperate and contribute depending only on the value of their discount factor δ, and not on the value of their current reputation R.

Proof. This immediately from the above two lemmas, as the actor's current reputation R is absent from both equations (3.2) and (3.3). In a PBE, using the reasoning in Lemma 3.2, we deduce that actors play C_1 or D_1 given the opportunity to do so depending solely on the value of their discount factor δ and on the lifetime benefit of achieving reputation $\mathcal{C}_1$ rather than reputation $\mathcal{D}_1$—which does not depend on their current reputation R (but instead, notably depends on the chooser's strategy, as shown below). Using the reasoning in Lemma 3.3, we similarly deduce that actors plays C_2 or D_2 depending only on their δ and the lifetime benefit of achieving reputation $\mathcal{C}_2$ rather than reputation $\mathcal{D}_2$. The strategy of actors does not therefore depend on their current reputation. □

3.3.4 Possible equilibrium chooser strategies for non-empty reputations σ^*_{ch}

Using Lemmas (3.2-3.4), we deduce that reputation must incentivize first-order cooperation for there to be a positive fraction of would-be cooperators, who would reciprocate if trusted, and that reputation must incentivize both forms of cooperation for there to be a postive fraction of contributors—and therefore for the institution to receive contributions in any meaningful way. This restricts the possibilities for σ^*_{ch}.

Lemma 3.5: If some actors cooperate, cooperation is incentivized by reputation

Consider a PBE (σ, μ^∞), with $\sigma = (\sigma_{ch}, \sigma_{ac,1}, \sigma_{ac,2}) \in \mathcal{S}$, and a non-trivial subset of discount factors $I \subset \Delta$, such that $\mathbb{P}(\delta \in I) > 0$.

$$\forall \delta \in I, \sigma_{ac,1}(R, \delta) = C_1 \implies \begin{cases} \sigma_{ch}(\mathcal{C}_1) = T, \\ \sigma_{ch}(\mathcal{D}_1) = \neg T. \end{cases}$$

In a PBE in which a non-trivial subset of actors cooperate, reputation incentivizes cooperation—choosers trust given $\mathcal{C}_1$, and distrust given $\mathcal{D}_1$.

Proof. This follows from the above lemmas, and the fact that net cost of cooperation is positive: $c_1 - \gamma - \beta > 0$. Given the opportunity to do so, playing C_1 instead of D_1 leads to an immediate payoff loss, which can only be upset by a future gain, reflected in the difference $U_\delta(\sigma \mid_{\mathcal{C}_1}) - U_\delta(\sigma \mid_{\mathcal{D}_1})$, as visible in equation (3.2).

In a PBE in which a non-trivial subset of actors cooperate, this difference must therefore be positive; which requires that choosers trust given $\mathcal{C}_1$ and distrust given $\mathcal{D}_1$. Note that we will calculate this difference in continuation payoffs in the following section. Here, it suffices to note that $U_\delta(\sigma \mid_{\mathcal{C}_1}) - U_\delta(\sigma \mid_{\mathcal{D}_1}) = 0$ if choosers play the same action given both of these reputations, and that $U_\delta(\sigma \mid_{\mathcal{C}_1}) - U_\delta(\sigma \mid_{\mathcal{D}_1}) \leq 0$ if they trust given $\mathcal{D}_1$ and distrust given $\mathcal{C}_1$. □

Lemma 3.6: If some actors contribute, both forms of cooperation are incentivized by reputation

Consider a PBE (σ, μ^∞), with $\sigma = (\sigma_{ch}, \sigma_{ac,1}, \sigma_{ac,2}) \in \mathcal{S}$, and a non-trivial subset of discount factors $I \subset \Delta$, such that $\mathbb{P}(\delta \in I) > 0$.

$$\forall \delta \in I, \sigma_{\text{ac},2}(R, \delta) = C_2 \implies \begin{cases} \sigma_{\text{ch}}(\mathcal{C}_1) = T \quad \text{and} \quad \sigma_{\text{ch}}(\mathcal{C}_2) = T \\ \sigma_{\text{ch}}(\mathcal{D}_1) = \neg T \quad \text{and} \quad \sigma_{\text{ch}}(\mathcal{D}_2) = \neg T \end{cases}$$

In a PBE in which a non-trivial subset of actors contribute, reputation incentivizes both forms of cooperation—choosers trust given $\mathcal{C}_1$ and $\mathcal{C}_2$, and distrust given $\mathcal{D}_1$ and $\mathcal{D}_2$.

Proof. Let (σ, μ^∞) be a PBE, with $\sigma = (\sigma_{ch}, \sigma_{ac,1}, \sigma_{ac,2}) \in \mathcal{S}$. We assume the existence of a non-trivial subset of discount factors $I \subset \Delta$ such that every actor of discount factor $\delta \in I$ contributes, whatever her reputation.

As in the proof just above, using Lemma 3.3 and 3.4, and the fact that $c_2 > 0$, we deduce that choosers must trust given $\mathcal{C}_2$ and distrust given $\mathcal{D}_2$. Indeed, given the opportunity to do so, playing C_2 instead of D_2 leads to an immediate payoff loss, which can only be upset by a future gain, that will be reflected in the difference $U_\delta(\sigma \mid_{\mathcal{C}_2}) - U_\delta(\sigma \mid_{\mathcal{D}_2})$, as visible in equation (3.3). We conclude as above (and calculate this difference in the next section).

To show that choosers must also incentivize first-order cooperation, we begin by recalling that the strategy of actors is stationary following Lemma 3.4, and then calculate choosers' expected payoff from playing T given information $\mathcal{C}_1$, and given information $\mathcal{D}_1$. Note that we will return to, and rigorously calculate, choosers' payoff in section 5; here, it suffices to note that this payoff must be positive in the first case, and negative in the second case.

Let I_{C_1} be the subset of Δ over which actors cooperate, and $I_{D_1} = \Delta \setminus I_{C_1}$ be the subset of Δ over which they defect. Since choosers trust given $\mathcal{C}_2$, as we just showed, and since $k > 0$, we deduce that $\mathbb{P}(\delta \in I_{C_1}) > 0$. Actors must cooperate over a non-trivial interval, otherwise trusting given $\mathcal{C}_2$ would be net costly.

Faced with an actor of reputation $\mathcal{C}_1$, choosers then use Bayes' rule to form posteriors, and infer that the actor's discount factor belongs to I_{C_1}—and therefore that she will reciprocate again, since her strategy is stationary.

In other words, given information $\mathcal{C}_1$ about an actor, that actor reciprocates with certainty. If choosers then trust, they gains payoff:

$$-k + 1 \times b = b - k > 0,$$

which is strictly larger than 0, the payoff of distrusting. In a PBE, choosers therefore trust given $\mathcal{C}_1$.

We conclude by, similarly, showing that $\mathbb{P}(\delta \in I_{D_1}) > 0$: since choosers distrust given $\mathcal{D}_2$, actors must cheat with positive probability—otherwise, trusting given $\mathcal{D}_2$ would yield payoff $-k+b>0$, and it would be strictly beneficial to deviate to doing so.

Faced with an actor of reputation $\mathcal{D}_1$, choosers then use Bayes' rule to form posteriors, and infer that the actor's discount factor belongs to I_{D_1}—and therefore that she will cheat again, since her strategy is stationary.

In other words, if choosers trust an actor of reputation $\mathcal{D}_1$, they gain payoff:

$$-k + 0 \times b = -k < 0,$$

which is strictly smaller than the payoff of distrusting, 0. In a PBE, choosers distrust given $\mathcal{D}_1$.

□

3.3.5 Three types of PBE

The last two lemmas restrict the possibilities for σ^*_{ch}. We deduce that there are in fact only three cases that can be part of a PBE.

First case: reputation does not incentivize either form of cooperation. Following Lemmas 3.5 and 3.6, every actor then defects and free-rides when given the chance (except perhaps for a negligible subset of actor types which occurs with probability 0). It follows that choosers distrust given any reputation in a perfect equilibrium; we obtain a trivially uncooperative PBE, in which every actor plays D_1 and D_2, and choosers always play $\neg T$.

Second case: reputation incentivizes first-order cooperation, but not second-order cooperation. In such a situation, following Lemma 3.6, every actor free-rides when given the chance (except perhaps for a negligible subset of actors). In section 6, we derive the conditions under which we obtain a PBE in which choosers trust given $\mathcal{C}_1$ and distrust given $\mathcal{D}_1$ in a specific parameter case—when the probability of observation in the institution game is $p_2 = 0$.

Third and final case: reputation incentivizes first- and second-order cooperation. As we just saw, this means that choosers trust given $\mathcal{C}_1$ and $\mathcal{C}_2$, and distrust given $\mathcal{D}_1$ and $\mathcal{D}_2$—in other words, this fully determines σ^*_{ch}. The only thing left to determine is the probability $\theta \in [0,1]$ that they trust given empty reputation.

We call such a situation an **institution equilibrium**—this is the only case in which the institution receives contributions in any meaningful way, and can have any effect on first-order cooperation. In section 4, we show that for every possible θ, actors' strategy is fully determined. In section 5, we determine the equilibrium value of θ, which in the parameter range we study in section 7 is unique. This prompts us to call this strategy profile *the* institution equilibrium, rather than an institution equilibrium from here on, in anticipation of the below results. In section 5, we derive the domain of existence of the institution equilibrium.

4 Institution equilibrium: actor strategy

In this section, we characterize actors' strategy in the institution equilibrium, in which we have shown that σ^*_{ch} is determined, but that choosers may trust given empty reputation with any probability $\theta \in [0,1]$. We show that, necessarily, actors cooperate if and only if their discount rate exceeds a certain threshold $\hat{\delta}_1(\theta)$ (in section 4.3), and that they contribute if and only if their discount rate exceeds another threshold $\hat{\delta}_2(\theta)$ (in section 4.4). We also define and calculate actors' normalized payoff in the institution equilibrium, as well as the steady state of their reputation, and the long-run level of cooperation.

4.1 Objective and simplifying notations

4.1.1 Objective

Throughout this section, we assume that choosers trust given $\mathcal{C}_1$ and $\mathcal{C}_2$, distrust given $\mathcal{D}_1$ and $\mathcal{D}_2$, and trust given $\emptyset$ with probability $\theta \in [0,1]$. We denote such a strategy by $\sigma^{inst,\theta}_{ch}$. As we just saw, following Lemma 3.6, this is the form that choosers' strategy must take for an institution to be established in any meaningful way.

We assume the existence of a PBE (σ, μ^∞), with $\sigma = (\sigma^{inst,\theta}_{ch}, \sigma_{ac})$, and call this the institution equilibrium. Our objective in this section is to characterize actors' strategy σ_{ac}, and show that it is uniquely determined. To do so, we first introduce a set of condensed notations (sections 4.1.2-4.1.5), all of which omit the (fixed) strategy profile σ for concision. Later, in section 5, we will show that θ is also uniquely determined, and derive the domain of existence of the institution equilibrium.

4.1.2 Reputational benefit

Note that when choosers play according to $\sigma_{ch}^{inst,\theta}$, reputation incentivizes first- and second-order cooperation *equally*—for any actor, the benefit of attaining reputation $\mathcal{C}_1$ rather than $\mathcal{D}_1$ is the same as the benefit of attaining reputation $\mathcal{C}_2$ rather than $\mathcal{D}_2$ (in our set up, reputational incentives are the fruit of only one decision, namely choosers' decision to trust an actor).

Given discount factor $\delta \in \Delta$, let $U_\delta^G \equiv U_\delta(\sigma\ |_{\mathcal{C}_1}) = U_\delta(\sigma\ |_{\mathcal{C}_2})$ be the actor's lifetime payoff in a continuation game associated with $\mathcal{C}_1$ or $\mathcal{C}_2$—in which case we say that the actor is in *good standing*. Similarly, we define $U_\delta^B \equiv U_\delta(\sigma\ |_{\mathcal{D}_1}) = U_\delta(\sigma\ |_{\mathcal{D}_2})$, and say that the actor is in *bad standing*, when her reputation is $\mathcal{D}_1$ or $\mathcal{D}_2$. Finally, we define $U_\delta^\emptyset \equiv U_\delta(\sigma\ |_\emptyset)$, and say that the actor is in *null standing*, when her reputation is $\emptyset$.

We define the **reputational benefit** of good behavior (or simply reputational benefit) R_δ to be the difference in continuation payoffs given good vs. bad standing, i.e. we define:

$$R_\delta \equiv U_\delta^G - U_\delta^B.$$

4.1.3 Normalized actor payoff

Since actors start out in null standing, the lifetime payoff of an actor of type δ is the discounted sum: $U_\delta(\sigma) = U_\delta^\emptyset$. Her normalized payoff $\overline{U}_\delta$ is obtained by multiplying the discounted sum by $(1-\delta)$:

$$\overline{U}_\delta \equiv (1-\delta)U_\delta^\emptyset. \tag{4.1}$$

In our numerical results, in section 7, we rely on the expected value of $\overline{U}_\delta$ given a certain distribution of discount factors—that is, the average normalized payoff of a randomly selected actor.

4.1.4 Condensed payoffs

Let $\mathbf{r_C} \equiv r - c_1 + \beta$ be the stage payoff of the actor if she is trusted and reciprocates, taking into account the potential institutional reward $\beta \geq 0$. Similarly, let $\mathbf{r_D} \equiv r - \gamma$ be her stage payoff if is trusted and cheats, let $\mathbf{c_1} \equiv \mathbf{r_D} - \mathbf{r_C} = r - (\beta + \gamma)$ be the cost of cooperation, and $\mathbf{p_1} = p_1 + \pi_1$ be the probability of cooperation—all of which take into account the effect of the institution in the proposed equilibrium σ (which notably depends on actors' prescribed contribution behavior).

Using our new notations, for any R and δ, we can rewrite the condition (3.2), obtained in Lemma 3.2, as:

$$\sigma_{ac,1}(R,\delta) = C_1 \iff \mathbf{c_1} \leq \delta \times \mathbf{p_1} \times R_\delta, \tag{3.2'}$$

and rewrite the condition (3.3) for second-order cooperation, obtained in Lemma 3.3, as:

$$\sigma_{ac,2}(R,\delta) = C_2 \iff c_2 \leq \delta \times p_2 \times R_\delta. \tag{3.3'}$$

We use our condensed notations to reduce the size of our calculations in the proofs below.

4.1.5 Partition of the set of possible discount factors Δ

As we have seen, actors' strategy is stationary (3.4): they reciprocate and contribute depending only on the value of their discount factor δ. We denote by $I_{C_1} \subset \Delta$ the subset of discount factors for which $\sigma_{ac,1}$ prescribes reciprocation, that is the maximum interval such that, $\forall R \in \mathcal{R}, \forall \delta \in I_{C_1}, \sigma_{ac,1}(R,\delta) = C_1$. We denote by $I_{D_1} \equiv \Delta \setminus I_{C_1}$ the subset of discount factor for which $\sigma_{ac,1}$ prescribes cheating; and we analogously define I_{C_2} and I_{D_2}.

The intersections of these intervals partition the set of possible discount factors Δ into four, depending on the two actions prescribed by σ_{ac} in both games. For instance, an actor of discount factor $\delta \in I_{C_1} \cap I_{C_2}$ plays C_1 and C_2 each time she is given the opportunity to, throughout the repeated game. Below, we show that these sets take a simple expression in a PBE, and that everything can be reduced to two threshold discount factors.

4.2 Derivation of the reputational benefit R_δ

Using a bit of algebra (Lemma 4.1) and the notations introduced above, we derive the value of an actor's reputational benefit R_δ as a function of δ (Lemma 4.2).

Lemma 4.1: Continuation payoff given empty reputation

Assume that choosers play $\sigma_{ch}^{inst,\theta}$, and take $\sigma = (\sigma_{ch}^{inst,\theta}, \sigma_{ac})$ and μ^∞ such that (σ, μ^∞) is a PBE. For any $\delta \in \Delta$, we have:

$$U_\delta^\emptyset = \theta U_\delta^G + (1-\theta) U_\delta^B, \tag{4.2}$$

$$U_\delta^\emptyset - U_\delta^B = \theta R_\delta, \tag{4.3}$$

$$U_\delta^G - U_\delta^\emptyset = (1-\theta) R_\delta. \tag{4.4}$$

Proof. Take our strategy profile $\sigma = (\sigma_{ch}^{inst,\theta}, \sigma_{ac}) \in \mathcal{S}$, and μ^∞ such that (σ, μ^∞) is a PBE. Since actors' strategy is stationary (Lemma 3.4), the only things that affect their continuation payoff are their current standing and their discount factor—the latter defining her actions throughout her repeated game.

When in good standing, actors are trusted in the trust game, in which case they subsequently have an opportunity to act. In the institution game, their standing does not affect their ability to act, and they are always able to either contribute or free-ride.

When in bad standing, the only thing that changes is that they are distrusted in the trust game. When in null standing, the only thing that changes is that they are trusted with probability probability θ, and distrusted with probability $1-\theta$.

In other words, for an actor of any discount factor δ, being in null standing is equivalent to being in good standing with probability θ, and bad standing with probability $1-\theta$. We deduce:

$$U_\delta^\emptyset = \theta U_\delta^G + (1-\theta) U_\delta^B. \tag{4.2}$$

The other two equations are deduced from this equation and the definition of R_δ, using $U_\delta^G = R_\delta + U_\delta^B$ to obtain (4.3), and $U_\delta^B = U_\delta^G - R_\delta$ to obtain (4.4). □

Lemma 4.2: Reputational benefit

Assume that choosers play $\sigma_{ch}^{inst,\theta}$, and take $\sigma = (\sigma_{ch}^{inst,\theta}, \sigma_{ac})$ and μ^∞ such that (σ, μ^∞) is a PBE. For any $\delta \in \Delta$, we have:

$$R_\delta = \begin{cases} \frac{q(r-\gamma)}{1+q\delta(p_1+\pi_1)\theta} & \text{if } \delta \in I_{D_1}, \\ \frac{q(r-c_1+\beta)}{1-q\delta(p_1+\pi_1)(1-\theta)} & \text{if } \delta \in I_{C_1}. \end{cases} \tag{4.5}$$

Proof. Take our strategy profile $\sigma = (\sigma_{ch}^{inst,\theta}, \sigma_{ac}) \in \mathcal{S}$, and μ^∞ such that (σ, μ^∞) is a PBE. As we have seen, the intersections $I_{C_1} \cap I_{C_2}$, $I_{C_1} \cap I_{D_2}$, $I_{D_1} \cap I_{C_1}$, and $I_{D_1} \cap I_{D_2}$ partition the set of possible discount factors into four, according to the four possible pairs of actor actions. For instance, every actor whose discount factor verifies $\delta \in I_{C_1} \cap I_{C_2}$ cooperates and contributes throughout her repeated game.

Using this partition, we can calculate R_δ four times, each time taking the actors' actions as given. As we will show, only behavior in the trust game affects the value of R_δ, meaning that we only need to partition between I_{C_1} and I_{D_1}.

To begin, let us consider the case of an actor whose discount factor verifies $\delta \in I_{C_1} \cap I_{C_2}$. Given bad standing, such an actor isn't trusted in the trust game, earning payoff 0, following which her standing becomes null, with certainty (since she does not act). In the institution game, she contributes, earning payoff $-c_2$, following which her standing becomes good if she is observed, with probability p_2, and null otherwise, with probability $1-p_2$. Since the trust game is drawn with probability q and the institution game with probability $1-q$, we deduce:

$$U_\delta^B = q \times (0 + \delta \times [1 \times U_\delta^\emptyset]) + (1-q) \times (-c_2 + \delta \times [p_2 \times U_\delta^G + (1-p_2) \times U_\delta^\emptyset]), \qquad \forall \delta \in I_{C_1} \cap I_{C_2}.$$

Given good standing, this actor is trusted in the trust game, and reciprocates that trust, earning payoff $\mathbf{r_C}$ instead of 0, following which she achieves good standing with probability $\mathbf{p_1}$, and null standing with probability $1-\mathbf{p_1}$. In other words:

$$U_\delta^G = q \times (\mathbf{r_C} + \delta \times [\mathbf{p_1} \times U_\delta^G + (1-\mathbf{p_1}) \times U_\delta^\emptyset]) + (1-q) \times (-c_2 + \delta \times [p_2 \times U_\delta^G + (1-p_2) \times U_\delta^\emptyset]), \quad \forall \delta \in I_{C_1} \cap I_{C_2}.$$

Subtracting U_δ^B to U_δ^G, the right parts of each expressions simplify—since the actor's standing does not affect her ability to act in the institution game, her action in that game (here, C_2) does not affect her reputational benefit. We obtain:

$$R_\delta = q \times (\mathbf{r_C} + \delta \times [\mathbf{p_1} \times U_\delta^G + (1-\mathbf{p_1}) \times U_\delta^\emptyset]) - q \times (0 + \delta \times [1 \times U_\delta^\emptyset]), \qquad \forall \delta \in I_{C_1} \cap I_{C_2},$$

which simplifies into:

$$R_\delta = q \times (\mathbf{r_C} + \delta \times \mathbf{p_1}(U_\delta^G - U_\delta^\emptyset)), \qquad \forall \delta \in I_{C_1} \cap I_{C_2}.$$

We obtain the same expression for $\delta \in I_{C_1} \cap I_{D_2}$—since, once again, R_δ is unaffected by behavior in the institution game (in this case, the right parts of U_δ^B and U_δ^G are both equal to $(1-q) \times (0 + \delta \times [p_2 \times U_\delta^B + (1-p_2) \times U_\delta^\emptyset]))$. In other words, the above expression is valid for any $\delta \in I_{C_1}$.

Replacing $U_\delta^G - U_\delta^\emptyset$ using equation (4.4), we deduce:

$$R_\delta = q \times (\mathbf{r_C} + \delta \times \mathbf{p_1} \times (1-\theta) R_\delta(\sigma)), \qquad \forall \delta \in I_{C_1},$$

which, re-arranging, is equivalent to:

$$R_\delta \times [1 - q \times \delta \times \mathbf{p_1} \times (1-\theta)] = q \times \mathbf{r_C}, \qquad \forall \delta \in I_{C_1},$$

and, re-arranging once again, is equivalent to:

$$R_\delta = \frac{q \times \mathbf{r_C}}{1 - q \times \delta \times \mathbf{p_1} \times (1-\theta)}, \qquad \forall \delta \in I_{C_1}.$$

Replacing our condensed payoffs with their value, we obtain:

$$R_\delta = \frac{q \times (r - c_1 + \beta)}{1 - q \times \delta \times (p_1 + \pi_1) \times (1-\theta)}, \qquad \forall \delta \in I_{C_1}.$$

This proves the lower part of the (4.5). To prove the remaining upper part of the equation, and prove the lemma, we calculate R_δ directly for an actor of discount factor $\delta \in I_{D_1}$. Given bad standing, such an actor isn't trusted in the trust game, earning payoff 0, following which her standing becomes null, with certainty. Given good standing, this actor is trusted in the trust game, earning payoff $\mathbf{r_D}$, following which her standing becomes bad with probability $\mathbf{p}_1$, and null with probability $1 - \mathbf{p}_1$. Her behavior in the institution game does not matter to R_δ, since it simplifies in both cases, whether her discount factor verifies $\delta \in I_{D_1} \cap I_{C_2}$ or $\delta \in I_{D_1} \cap I_{D_2}$.

Subtracting her prospects in the trust game given bad standing to those same prospects given good standing, we deduce:

$$R_\delta = q \times (\mathbf{r_D} + \delta \times [\mathbf{p_1} \times U_\delta^B + (1-\mathbf{p_1}) \times U_\delta^\emptyset]) - q \times (0 + \delta \times [1 \times U_\delta^\emptyset]), \qquad \forall \delta \in I_{D_1},$$

which simplifies to:

$$R_\delta = q \times (\mathbf{r_D} + \delta \times \mathbf{p_1}(U_\delta^B - U_\delta^\emptyset)), \qquad \forall \delta \in I_{D_1}.$$

Replacing $U_{\delta_-}^B - U_{\delta_-}^\emptyset$ using equation (4.3), we deduce:

$$R_\delta = q \times (\mathbf{r_D} + \delta \times \mathbf{p_1} \times (-\theta R_\delta)), \qquad \forall \delta \in I_{D_1},$$

which is equivalent to:

$$R_\delta \times [1 + q \times \delta \times \mathbf{p_1} \times \theta] = q \times \mathbf{r_D}, \qquad \forall \delta \in I_{D_1},$$

and to:

$$R_\delta = \frac{q \times \mathbf{r_D}}{1 + q \times \delta \times \mathbf{p_1} \times \theta}, \qquad \forall \delta \in I_{D_1}.$$

Witout our uncondensed payoffs we obtain:

$$R_\delta = \frac{q \times (r - \gamma)}{1 + q \times \delta \times (p_1 + \pi_1) \times \theta}, \qquad \forall \delta \in I_{D_1}.$$

□

4.3 Threshold discount factor for first-order cooperation

Using the general condition for cooperation (3.2) (shown in Lemma 3.2), we deduce that, in the institution equilibrium, sufficiently patient actors cooperate, and sufficiently impatient actors defect—the indifference point being captured by a single threshold value $\hat{\delta}_1(\theta)$.

Proposition 4.1: Threshold discount factor for cooperation

Assume that choosers play $\sigma_{ch}^{inst,\theta}$, and take $\sigma = (\sigma_{ch}^{inst,\theta}, \sigma_{ac})$ and μ^∞ such that (σ, μ^∞) is a PBE. Then:

$$I_{D_1} =]0, \hat{\delta}_1(\theta)[\cap \Delta,$$

and:

$$I_{C_1} = [\hat{\delta}_1(\theta), 1[\cap \Delta,$$

where:

$$\hat{\delta}_1(\theta) \equiv \frac{c_1 - (\beta + \gamma)}{(p_1 + \pi_1)q[r - \gamma - \theta(c_1 - (\beta + \gamma))]}. \tag{4.6}$$

In the institution equilibrium, sufficiently patient ($\delta \geq \hat{\delta}_1(\theta)$) actors always reciprocate the trust of choosers, and sufficiently impatient ($\delta < \hat{\delta}_1(\theta)$) actors always cheat—the value of the threshold separating reciprocators from cheaters being a function of the likelihood θ of being trusted given empty reputation.

Proof. Take our strategy profile $\sigma = (\sigma_{ch}^{inst,\theta}, \sigma_{ac}) \in \mathcal{S}$, and μ^∞ such that (σ, μ^∞) is a PBE. Using the general condition for cooperation (3.2), shown in Lemma 3.2, our condensed payoffs, and the definition of I_{C_1}, we have:

$$\delta \in I_{C_1} \iff \mathbf{c_1} \leq \delta \times \mathbf{p_1} \times R_\delta.$$

In the previous lemma, we showed that:

$$R_\delta = \frac{q \times \mathbf{r_C}}{1 - q \times \delta \times \mathbf{p_1} \times (1 - \theta)}, \qquad \forall \delta \in I_{C_1}.$$

Replacing, we deduce:

$$\begin{aligned}
\delta \in I_{C_1} &\iff \mathbf{c_1} \leq \delta \times \mathbf{p_1} \times \frac{q \times \mathbf{r_C}}{1 - q \times \delta \times \mathbf{p_1} \times (1 - \theta)}, \\
&\iff \mathbf{c_1} \times [1 - q \times \delta \times \mathbf{p_1} \times (1 - \theta)] \leq \delta \times \mathbf{p_1} \times q \times \mathbf{r_C}, \\
&\iff \mathbf{c_1} \leq \delta \times [\mathbf{p_1} \times q \times \mathbf{r_C} + q \times \mathbf{p_1} \times (1 - \theta) \times \mathbf{c_1}] \\
&\iff \delta \geq \frac{\mathbf{c_1}}{\mathbf{p_1} \times q \times (\mathbf{r_C} + (1 - \theta) \times \mathbf{c_1})}.
\end{aligned}$$

Using $\mathbf{r_C} + 1 \times \mathbf{c_1} = \mathbf{r_D}$, and replacing our condensed payoffs, we deduce:

$$\begin{aligned}
\delta \in I_{C_1} &\iff \delta \geq \frac{\mathbf{c_1}}{\mathbf{p_1} \times q \times (\mathbf{r_D} - \theta \times \mathbf{c_1})}, \\
&\iff \delta \geq \frac{c_1 - (\beta + \gamma)}{(p_1 + \pi_1) \times q \times (r - \gamma - \theta \times (c_1 - (\beta + \gamma)))}.
\end{aligned}$$

This proves that I_{C_1} is composed of all the possible discount factor values that are greater or equal to the threshold $\hat{\delta}_1(\theta)$, as given by condition (4.6), and therefore $I_{C_1} = [\hat{\delta}_1(\theta), 1[\cap \Delta$. Since $I_{D_1} = \Delta \setminus I_{C_1}$, we deduce $I_{D_1} =]0, \hat{\delta}_1[\cap \Delta$, proving the proposition.

For good measure, let us note that we obtain the same result when focusing on I_{D_1}. The same general condition for cooperation shows that:

$$\delta \in I_{D_1} \iff \mathbf{c_1} > \delta \times \mathbf{p_1} \times R_\delta.$$

Using the expression $R_\delta = \frac{q\times\mathbf{r_D}}{1+q\times\delta\times\mathbf{p_1}\times\theta}$, valid over the entire set I_{D_1}, we deduce:

$$\begin{aligned}
\delta \in I_{D_1} &\iff \mathbf{c_1} > \delta\times\mathbf{p_1}\times\frac{q\times\mathbf{r_D}}{1+q\times\delta\times\mathbf{p_1}\times\theta},\\
&\iff \mathbf{c_1}\times[1+q\times\delta\times\mathbf{p_1}\times\theta] > \delta\times\mathbf{p_1}\times q\times\mathbf{r_D},\\
&\iff \mathbf{c_1} > \delta\times\mathbf{p_1}\times q\times[\mathbf{r_D}-\theta\times\mathbf{c_1}],\\
&\iff \delta < \frac{\mathbf{c_1}}{\mathbf{p_1}\times q\times(\mathbf{r_D}-\theta\times\mathbf{c_1})}\\
&\iff \delta < \hat{\delta}_1(\theta).
\end{aligned}$$

□

We refer to $\hat{\delta}_1(\theta)$ as the **difficulty of cooperation in the institution equilibrium for** θ. Since $\hat{\delta}_1(\theta) > 0$, we always obtain a positive fraction of would-be cheaters, who would cheat if trusted. As evoked above (in the demonstration of Lemma 3.6), we need a positive fraction of would-be cooperators to obtain an institution equilibrium (otherwise it is beneficial to distrust even given $\mathcal{C}_1$), which can only occur if $\hat{\delta}_1(\theta) < 1$; cooperation cannot be 'too difficult'.

4.4 Threshold discount factor for second-order cooperation

Using the general condition for contribution (3.3) (shown in Lemma 3.3), we similarly show that sufficiently patient actors contribute, and sufficiently impatient actors free-ride.

We refer to the indifference point $\hat{\delta}_2(\theta)$ as the **difficulty of second-order cooperation in the institution equilibrium for** θ. There are two cases, depending on whether this threshold is smaller, or greater, than $\hat{\delta}_1(\theta)$—that is, on whether second-order cooperation can be said to be 'easier', or 'more difficult', than first-order cooperation.

Proposition 4.2: Threshold discount factor for second-order cooperation

Assume that choosers play $\sigma_{ch}^{inst,\theta}$, and take $\sigma = (\sigma_{ch}^{inst,\theta}, \sigma_{ac})$ and μ^∞ such that (σ, μ^∞) is a PBE. Then:

$$I_{D_2} =]0, \hat{\delta}_2(\theta)[\cap \Delta,$$

and:

$$I_{C_2} = [\hat{\delta}_2(\theta), 1[\cap \Delta,$$

where:

$$\hat{\delta}_2(\theta) \equiv \begin{cases} \frac{c_2}{q[p_2(r-\gamma)-(p_1+\pi_1)\theta c_2]} & \text{if } \hat{\delta}_2(\theta) < \hat{\delta}_1(\theta),\\ \frac{c_2}{q[p_2(r-c_1+\beta)+(p_1+\pi_1)(1-\theta)c_2]} & \text{if } \hat{\delta}_2(\theta) \geq \hat{\delta}_1(\theta).\end{cases} \tag{4.7}$$

In the institution equilibrium, sufficiently patient ($\delta \geq \hat{\delta}_2(\theta)$) actors always contribute to the institution, and sufficiently impatient ($\delta < \hat{\delta}_2(\theta)$) actors always free-ride—the value of the threshold separating contributors from free-riders being a function of the likelihood θ of being trusted given empty reputation, as well as whether second-order cooperation is more difficult than first-order cooperation ($\hat{\delta}_2(\theta) \geq \hat{\delta}_1(\theta)$), or less difficult ($\hat{\delta}_2(\theta) < \hat{\delta}_1(\theta)$) (the threshold $\hat{\delta}_1(\theta)$ is defined in Proposition 4.1).
What's more:

$$\hat{\delta}_2(\theta) < \hat{\delta}_1(\theta) \iff \frac{c_2}{p_2} < \frac{\mathbf{c_1}}{\mathbf{p_1}} = \frac{c_1-(\beta+\gamma)}{p_1+\pi_1}. \tag{4.8}$$

The easiest form of cooperation is the one with the lowest ratio of net cost divided by observability.

Proof. Take our strategy profile $\sigma = (\sigma_{ch}^{inst,\theta}, \sigma_{ac}) \in \mathcal{S}$, and μ^∞ such that (σ, μ^∞) is a PBE. Using equation (3.3) and a similar reasoning to the proof above, we deduce that actors contribute given the opportunity to do so if and only if their discount factor exceeds the threshold $\hat{\delta}_2(\theta)$ satisfying the equation:

$$c_2 = \hat{\delta}_2(\theta) p_2 R_{\hat{\delta}_2(\theta)}(\sigma).$$

There are two possibilities, depending on whether this threshold is smaller than $\hat{\delta}_1(\theta)$—in which case we must use the upper part of (4.5) to replace $R_{\hat{\delta}_2(\theta)}(\sigma)$—or larger than this threshold—in which case we must use the lower part of this equation.

First case (second-order cooperation is easier): when $\hat{\delta}_2(\theta) < \hat{\delta}_1(\theta)$, the critical reputational benefit is obtained using the upper part of (4.5). We obtain:

$$c_2 = \hat{\delta}_2(\theta)p_2\frac{q(r-\gamma)}{1+\hat{\delta}_2(\theta)(p_1+\pi_1)q\theta}, \qquad \text{if } \hat{\delta}_2(\theta) < \hat{\delta}_1(\theta),$$

which is equivalent to:

$$\hat{\delta}_2(\theta) = \frac{c_2}{q[p_2(r-\gamma)-(p_1+\pi_1)\theta c_2]}, \qquad \text{if } \hat{\delta}_2(\theta) < \hat{\delta}_1(\theta).$$

Second case (second-order cooperation is more difficult): when $\hat{\delta}_2(\theta) \geq \hat{\delta}_1(\theta)$, we obtain:

$$c_2 = \hat{\delta}_2(\theta)p_2\frac{q(r-c_1+\beta)}{1-q\hat{\delta}_2(\theta)(p_1+\pi_1)(1-\theta)}, \qquad \text{if } \hat{\delta}_2(\theta) \geq \hat{\delta}_1(\theta),$$

which is equivalent to:

$$\hat{\delta}_2(\theta) = \frac{c_2}{q[p_2(r-c_1+\beta)+(p_1+\pi_1)(1-\theta)c_2]}, \qquad \text{if } \hat{\delta}_2(\theta) \geq \hat{\delta}_1(\theta).$$

Bringing both equations together, we have proven condition (4.7). Using condition (4.6), proven in the previous Proposition, the upper part of (4.7), as well as the condensed notations introduced above, we deduce that if $\hat{\delta}_2(\theta) < \hat{\delta}_1(\theta)$, then we must have:

$$\frac{c_2}{q[p_2\mathbf{r_D}-\mathbf{p_1}\theta c_2]} < \frac{\mathbf{c_1}}{\mathbf{p_1}q[\mathbf{r_D}-\theta\mathbf{c_1}]},$$

which is equivalent to:

$$c_2\mathbf{p_1}(\mathbf{r_D}-\theta\mathbf{c_1}) < \mathbf{c_1}(p_2\mathbf{r_D}-\mathbf{p_1}\theta c_2),$$

and also to:

$$c_2\mathbf{p_1}\mathbf{r_D} < \mathbf{c_1}p_2\mathbf{r_D},$$

and finally to:

$$\frac{c_2}{p_2} < \frac{\mathbf{c_1}}{\mathbf{p_1}}.$$

Using the bottom part of (4.7), we deduce that the above is also a sufficient condition. Second-order cooperation is easier than first-order cooperation if and only if its cost c_2 divided by the relevant probability of observation p_2 is smaller than the net cost of first-order cooperation $\mathbf{c_1}$ divided by the relevant total probability of observation $\mathbf{p_1}$. In other words:

$$\hat{\delta}_2(\theta) < \hat{\delta}_1(\theta) \iff \frac{c_2}{p_2} < \frac{\mathbf{c_1}}{\mathbf{p_1}} = \frac{c_1-\gamma-\beta}{p_1+\pi_1}. \tag{4.8}$$

□

4.5 Normalized actor payoff

Using our previous results, we calculate the normalized payoff of any actor in the institution equilibrium.

Lemma 4.3: Normalized actor payoff

Assume that choosers play $\sigma_{ch}^{inst,\theta}$, and take $\sigma = (\sigma_{ch}^{inst,\theta}, \sigma_{ac})$ and μ^∞ such that (σ, μ^∞) is a PBE. For any $\delta \in \Delta$, we have:

$$\overline{U}_\delta = \begin{cases} [1-(1-q)p_2\delta]\theta R_\delta, & \text{if } \delta < \hat{\delta}_2(\theta), \\ (1-q)(-c_2), +[\theta+(1-q)p_2\delta(1-\theta)]R_\delta & \text{if } \delta \geq \hat{\delta}_2(\theta). \end{cases} \tag{4.9}$$

Proof. Take our strategy profile $\sigma = (\sigma_{ch}^{inst,\theta}, \sigma_{ac}) \in \mathcal{S}$, and μ^∞ such that (σ, μ^∞) is a PBE. We begin by calculating the continuation payoff of an actor of generic discount factor δ given bad standing—in which case, she is never trusted this round, and her action in the trust game has no bearing for her immediate payoffs. Following Proposition (4.2), there are two cases: either $\delta < \hat{\delta}_2(\theta)$, and the actor will free-ride if the institution game is drawn, or $\delta \geq \delta_2(\theta)$, and the actor will contribute if the institution game is drawn.

First case: $\delta < \hat{\delta}_2(\theta)$. If the trust game is drawn, the actor does not play and achieves null standing with certainty. If the institution game is drawn, the actor free-rides, and achieves bad standing if she is observed, and null standing otherwise. Thus, she obtains:

$$U_\delta^B = q \times (0 + \delta \times [1 \times U_\delta^\emptyset]) + (1-q) \times (0 + \delta \times [p_2 \times U_\delta^B + (1-p_2) \times U_\delta^\emptyset), \qquad \forall \delta < \hat{\delta}_2(\theta),$$

which, re-arranging, yields:

$$U_\delta^B = [q + (1-q)(1-p_2)] \times \delta U_\delta^\emptyset + (1-q)p_2 \times \delta U_\delta^B, \qquad \forall \delta < \hat{\delta}_2(\theta),$$

and, re-arranging once more:

$$U_\delta^B[1 - (1-q)p_2 \times \delta] = [1 - (1-q)p_2] \times \delta U_\delta^\emptyset, \qquad \forall \delta < \hat{\delta}_2(\theta).$$

Since $U_\delta^B = U_\delta^\emptyset - \theta R_\delta$, following equation (4.3), we deduce:

$$(U_\delta^\emptyset - \theta R_\delta)[1 - (1-q)p_2 \times \delta] = [1 - (1-q)p_2] \times \delta U_\delta^\emptyset, \qquad \forall \delta < \hat{\delta}_2(\theta),$$

which, re-arranging, yields:

$$U_\delta^\emptyset([1 - (1-q)p_2 \times \delta] - [1 - (1-q)p_2] \times \delta) = [1 - (1-q)p_2\delta]\theta R_\delta, \qquad \forall \delta < \hat{\delta}_2(\theta),$$

and, re-arranging once more:

$$U_\delta^\emptyset(1-\delta) = [1 - (1-q)p_2\delta]\theta R_\delta, \qquad \forall \delta < \hat{\delta}_2(\theta).$$

We recognize the actor's normalized payoff on the left, thus proving the upper part of (4.9). To prove the lower part of the equation, and thus the lemma, we consider the second case: $\delta \geq \hat{\delta}_2(\theta)$. In this case, in contrast to before, the actor contributes when given the opportunity, thus achieving good standing if observed. Her payoff given bad standing is then:

$$U_\delta^B = q \times (0 + \delta \times [1 \times U_\delta^\emptyset]) + (1-q) \times (-c_2 + \delta \times [p_2 \times U_\delta^G + (1-p_2) \times U_\delta^\emptyset]), \qquad \forall \delta \geq \hat{\delta}_2(\theta),$$

which, re-arranging, yields:

$$U_\delta^B = [1 - (1-q)p_2]\delta U_\delta^\emptyset + (1-q)(-c_2 + p_2 \times \delta U_\delta^G), \qquad \forall \delta \geq \hat{\delta}_2(\theta).$$

By subtracting $(1-q)(p_2 \times \delta U_\delta^B)$ from both sides of the equation, and using $U_\delta^G - U_\delta^B = R_\delta$, we deduce:

$$U_\delta^B[1 - (1-q)p_2\delta] = [1 - (1-q)p_2]\delta U_\delta^\emptyset + (1-q)(-c_2) + (1-q)p_2 \times \delta R_\delta, \qquad \forall \delta \geq \hat{\delta}_2(\theta).$$

And, using $U_\delta^B = U_\delta^\emptyset - \theta R_\delta$ once again, which follows from (4.3), we deduce:

$$(U_\delta^\emptyset - \theta R_\delta)[1 - (1-q)p_2\delta] = [1 - (1-q)p_2]\delta U_\delta^\emptyset + (1-q)(-c_2) + [(1-q)p_2\delta]R_\delta, \qquad \forall \delta \geq \hat{\delta}_2(\theta),$$

which, re-arranging, yields:

$$U_\delta^\emptyset(1-\delta) = (1-q)(-c_2) + ([(1-q)p_2\delta] + \theta[1 - (1-q)p_2\delta]]R_\delta, \qquad \forall \delta \geq \hat{\delta}_2(\theta).$$

We recognize the actor's normalized payoff on the left, thus proving the bottom part of (4.9). □

4.6 Steady state of actors' reputation

In the institution equilibrium, the reputation of every actor follows a Markov process—depending on her actions, and therefore, as we have shown, depending on how her discount factor δ compares to both thresholds $\hat{\delta}_1(\theta)$ and $\hat{\delta}_2(\theta)$. For instance, if $\delta \geq \max\{\hat{\delta}_1(\theta), \hat{\delta}_2(\theta)\}$, then the actor cooperates in both games, and at the end of any given round, her reputation becomes $\emptyset$, $\mathcal{C}_1$ or $\mathcal{C}_2$—depending on the game that is drawn that round, whether or not she is observed, and her reputation at the beginning of the round (which affects her ability to cooperate); but not on her reputation in any round before.

Here, we characterize the steady state of any actor's reputation, which we also call her long-run reputation, assuming that $(c_2/p_2) < (\mathbf{c_1}/\mathbf{p_1})$—in which case, following Proposition 4.2, first-order cooperation is more difficult than second-order cooperation ($\hat{\delta}_1(\theta) \geq \hat{\delta}_2(\theta)$). There are then only three cases to consider: $\delta \geq \hat{\delta}_1(\theta)$, in which case the actor reciprocates and contributes; $\delta < \hat{\delta}_2(\theta)$, in which case the actor cheats and free-rides; and $\hat{\delta}_2(\theta) \leq \delta < \hat{\delta}_1(\theta)$, in which case the actor contributes but does not reciprocate.

We use the steady state of the actor's reputation to calculate and plot the long-run level of cooperation, defined just below. In the main document and our numerical solution (section 7), we assume $c_2 < \mathbf{c_1}$ and $p_2 > \mathbf{p_1}$, guaranteeing that first-order cooperation be more difficult than second-order cooperation, which is the case of interest for us. Were we to allow the converse to be true, we would need to prove a fourth lemma to replace 4.5, as actors of intermediary δ would then reciprocate but not contribute, rather than the opposite.

Lemma 4.4: Long-run reputation for a high patience actor

Assume that choosers play $\sigma_{ch}^{inst,\theta}$, and take $\sigma = (\sigma_{ch}^{inst,\theta}, \sigma_{ac})$ and μ^∞ such that (σ, μ^∞) is a PBE. Assume also that $(c_2/p_2) < (\mathbf{c_1}/\mathbf{p_1})$.

For any $\delta \geq \hat{\delta}_1(\theta)$, the reputation of discount factor δ follows a Markov process with three states: $\mathcal{C}_1$, $\mathcal{C}_2$ and $\emptyset$. The steady state $\pi^H \equiv (\pi^H_{\mathcal{C}_1}, \pi^H_{\mathcal{C}_2}, \pi^H_\emptyset)$ of the actor's reputation is given by:

$$\pi^H_{\mathcal{C}_1} \equiv q\mathbf{p}_1 \frac{(1-q)p_2(1-\theta)+\theta}{1-q(1-\theta)\mathbf{p}_1}, \tag{4.10}$$

$$\pi^H_{\mathcal{C}_2} \equiv (1-q)p_2, \tag{4.11}$$

$$\pi^H_\emptyset \equiv 1 - q\mathbf{p}_1 \frac{(1-q)p_2(1-\theta)+\theta}{1-q(1-\theta)\mathbf{p}_1} - (1-q)p_2. \tag{4.12}$$

Proof. Take our strategy profile $\sigma = (\sigma_{ch}^{inst,\theta}, \sigma_{ac}) \in \mathcal{S}$, and μ^∞ such that (σ, μ^∞) is a PBE. We assume $(c_2/p_2) < (\mathbf{c_1}/\mathbf{p_1})$, and consider an actor of any discount factor $\delta \geq \hat{\delta}_1(\theta)$, assuming such a value is possible.

The actor plays C_1 and C_2 (or doesn't play) throughout the game. Her reputation can thus take three values during her life: $\mathcal{C}_1$, $\mathcal{C}_2$, and $\emptyset$. For any $t \geq 0$, we denote by $\pi^H_{\mathcal{C}_1}(t)$, $\pi^H_{\mathcal{C}_2}(t)$ and $\pi^H_\emptyset(t)$ the probability that this high patience actor is in state $\mathcal{C}_1$, $\mathcal{C}_2$, and $\emptyset$, respectively. By assumption, $\pi^H_{\mathcal{C}_1}(0) = \pi^H_{\mathcal{C}_2}(0) = 0$, and $\pi^H_\emptyset(0) = 1$.

Entering into any round $t \geq 1$, the actor has reputation $\mathcal{C}_1$ if and only if: in the previous round $t-1$, (i) the trust game was drawn, with probability q, (ii) she was trusted, with probability 1 if her reputation was $\mathcal{C}_1$ or $\mathcal{C}_2$, and probability θ if her reputation was $\emptyset$, and (iii) she was observed, with probability $\mathbf{p}_1$.

It follows that, for any $t \geq 1$:

$$\pi^H_{\mathcal{C}_1}(t) = q \times [1 \times \pi^H_{\mathcal{C}_1}(t-1) + 1 \times \pi^H_{\mathcal{C}_2}(t-1) + \theta \times \pi^H_\emptyset(t-1)] \times \mathbf{p}_1.$$

Entering into any round $t \geq 1$, the actor has reputation $\mathcal{C}_2$ if and only if: (i) the institution game was drawn, with probability $1-q$, and (ii) she was observed, with probability p_2. It follows that, for all $t \geq 1$:

$$\pi^H_{\mathcal{C}_2}(t) = (1-q) \times p_2.$$

These equations show that the actor's reputation follows a Markov process, as, for any $t \geq 1$, $\pi^H_{\mathcal{C}_1}(t)$, $\pi^H_{\mathcal{C}_2}(t)$ and $\pi^H_\emptyset(t) = 1 - \pi^H_{\mathcal{C}_1}(t) - \pi^H_{\mathcal{C}_2}(t)$ each only depend on the probabilities for round $t-1$.

Using both of the above equations, the steady state probabilities $\pi^H_{\mathcal{C}_1}, \pi^H_{\mathcal{C}_2}$, and $\pi^H_\emptyset$ must verify:

$$\begin{aligned}
\pi^H_{\mathcal{C}_1} &= q(\pi^H_{\mathcal{C}_1} + \pi^H_{\mathcal{C}_2} + \theta\pi^H_\emptyset)\mathbf{p}_1, \\
\pi^H_{\mathcal{C}_2} &= (1-q)p_2, \\
\pi^H_\emptyset &= 1 - \pi^H_{\mathcal{C}_1} - \pi^H_{\mathcal{C}_2}.
\end{aligned}$$

Replacing in the first equation, we deduce:

$$\pi^H_{\mathcal{C}_1} = q(\pi^H_{\mathcal{C}_1} + \pi^H_{\mathcal{C}_2} + \theta(1 - \pi^H_{\mathcal{C}_1} - \pi^H_{\mathcal{C}_2}))\mathbf{p}_1,$$

which, re-arranging, yields:

$$\pi^H_{\mathcal{C}_1}(1 - q(1-\theta)\mathbf{p}_1) = q(\pi^H_{\mathcal{C}_2}(1-\theta) + \theta)\mathbf{p}_1,$$

and, re-arranging once more:

$$\pi^H_{\mathcal{C}_1} = q\mathbf{p}_1 \frac{\pi^H_{\mathcal{C}_2}(1-\theta) + \theta}{1 - q(1-\theta)\mathbf{p}_1}.$$

Using $\pi^H_{\mathcal{C}_2} = (1-q)p_2$, we deduce all three proposed equations. □

Lemma 4.5: Long-run reputation for a medium patience actor

Assume that choosers play $\sigma^{inst,\theta}_{ch}$, and take $\sigma = (\sigma^{inst,\theta}_{ch}, \sigma_{ac})$ and μ^∞ such that (σ, μ^∞) is a PBE. Assume also that $(c_2/p_2) < (\mathbf{c_1}/\mathbf{p_1})$.

For any $\delta \in [\hat{\delta}_2(\theta), \hat{\delta}_1(\theta)[$, the reputation of an actor of discount factor δ follows a Markov process with three states: $\mathcal{D}_1$, $\mathcal{C}_2$ and $\emptyset$. The steady state $\pi^M \equiv (\pi^M_{\mathcal{D}_1}, \pi^M_{\mathcal{C}_2}, \pi^M_\emptyset)$ of the actor's reputation is given by:

$$\pi^M_{\mathcal{D}_1} \equiv q\mathbf{p}_1 \frac{(1-q)p_2(1-\theta) + \theta}{1 + q\theta\mathbf{p}_1}, \tag{4.13}$$

$$\pi^M_{\mathcal{C}_2} \equiv (1-q)p_2, \tag{4.14}$$

$$\pi^M_\emptyset \equiv 1 - q\mathbf{p}_1 \frac{(1-q)p_2(1-\theta) + \theta}{1 + q\theta\mathbf{p}_1} - (1-q)p_2. \tag{4.15}$$

Proof. Take our strategy profile $\sigma = (\sigma^{inst,\theta}_{ch}, \sigma_{ac}) \in \mathcal{S}$, and μ^∞ such that (σ, μ^∞) is a PBE. We assume $(c_2/p_2) < (\mathbf{c_1}/\mathbf{p_1})$, and consider any actor of discount factor $\delta \in [\hat{\delta}_2(\theta), \hat{\delta}_1(\theta)[$, assuming such a value is possible.

The actor plays D_1 and C_2 (or doesn't play) throughout the game. Her reputation can thus take three values during her life: $\mathcal{D}_1$, $\mathcal{C}_2$, and $\emptyset$. For any $t \geq 0$, we denote by $\pi^M_{\mathcal{D}_1}(t)$, $\pi^M_{\mathcal{C}_2}(t)$ and $\pi^M_\emptyset(t)$ the probability that this medium patience actor is in state $\mathcal{D}_1$, $\mathcal{C}_2$, and $\emptyset$, respectively. By assumption, $\pi^M_{\mathcal{D}_1}(0) = \pi^M_{\mathcal{C}_2}(0) = 0$, and $\pi^M_\emptyset(0) = 1$.

Entering into any round $t \geq 1$, the actor has reputation $\mathcal{D}_1$ if and only if: in the previous round $t-1$, (i) the trust game was drawn, with probability q, (ii) she was trusted, with probability 0 if her reputation was $\mathcal{D}_1$, probability 1 if her reputation was $\mathcal{C}_2$, and probability θ if her reputation was $\emptyset$, and (iii) she was observed, with probability $\mathbf{p}_1$.

It follows that, for any $t \geq 1$:

$$\pi^M_{\mathcal{D}_1}(t) = q \times [0 \times \pi^M_{\mathcal{D}_1}(t-1) + 1 \times \pi^M_{\mathcal{C}_2}(t-1) + \theta \times \pi^M_\emptyset(t-1)] \times \mathbf{p}_1.$$

Entering into any round $t \geq 1$, the actor has reputation $\mathcal{C}_2$ if and only if: (i) the institution game was drawn, with probability $1-q$, and (ii) she was observed, with probability p_2. It follows that, for all $t \geq 1$:

$$\pi^M_{\mathcal{C}_2}(t) = (1-q) \times p_2.$$

These equations show that the actor's reputation follows a Markov process, as, for any $t \geq 1$, $\pi^M_{\mathcal{C}_1}(t)$, $\pi^M_{\mathcal{C}_2}(t)$ and $\pi^M_\emptyset(t) = 1 - \pi^M_{\mathcal{D}_1}(t) - \pi^M_{\mathcal{C}_2}(t)$ each only depend on the probabilities for round $t-1$.

Using both of the above equations, the steady state probabilities $\pi^M_{\mathcal{D}_1}, \pi^M_{\mathcal{C}_2}$, and $\pi^M_\emptyset$ must verify:

$$\pi^M_{\mathcal{D}_1} = q(\pi^M_{\mathcal{C}_2} + \theta\pi^M_\emptyset)\mathbf{p}_1,$$
$$\pi^M_{\mathcal{C}_2} = (1-q)p_2,$$
$$\pi^M_\emptyset = 1 - \pi^M_{\mathcal{D}_1} - \pi^M_{\mathcal{C}_2}.$$

Replacing in the first equation, we deduce:

$$\pi^M_{\mathcal{D}_1} = q(\pi^M_{\mathcal{C}_2} + \theta(1 - \pi^M_{\mathcal{D}_1} - \pi^M_{\mathcal{C}_2}))\mathbf{p}_1,$$

which, re-arranging, yields:

$$\pi^M_{\mathcal{D}_1}(1 + q\theta\mathbf{p_1}) = q(\pi^M_{\mathcal{C}_2}(1-\theta) + \theta)\mathbf{p_1},$$

and, re-arranging once more:

$$\pi^M_{\mathcal{D}_1} = q\mathbf{p_1}\frac{\pi^M_{\mathcal{C}_2}(1-\theta) + \theta}{1 + q\theta\mathbf{p_1}}.$$

Using $\pi^M_{C_2} = (1-q)p_2$, we deduce all three proposed equations. □

Lemma 4.6: Long-run reputation for a low patience actor

Assume that choosers play $\sigma^{inst,\theta}_{ch}$, and take $\sigma = (\sigma^{inst,\theta}_{ch}, \sigma_{ac})$ and μ^∞ such that (σ, μ^∞) is a PBE. Assume also that $(c_2/p_2) < (\mathbf{c_1}/\mathbf{p_1})$.
For any $\delta < \hat{\delta}_2(\theta)$, the reputation of an actor of discount factor δ follows a Markov process with three states: $\mathcal{D}_1$, $\mathcal{D}_2$ and $\emptyset$. The steady state $\pi^L \equiv (\pi^L_{\mathcal{D}_1}, \pi^L_{\mathcal{D}_2}, \pi^L_\emptyset)$ of the actor's reputation is given by:

$$\pi^L_{\mathcal{D}_1} \equiv q\theta\mathbf{p_1}\frac{1-(1-q)p_2}{1+q\theta\mathbf{p_1}} \tag{4.16}$$

$$\pi^L_{\mathcal{D}_2} \equiv (1-q)p_2 \tag{4.17}$$

$$\pi^L_\emptyset \equiv 1 - q\theta\mathbf{p_1}\frac{1-(1-q)p_2}{1+q\theta\mathbf{p_1}} - (1-q)p_2 \tag{4.18}$$

Proof. Take our strategy profile $\sigma = (\sigma^{inst,\theta}_{ch}, \sigma_{ac}) \in \mathcal{S}$, and μ^∞ such that (σ, μ^∞) is a PBE. We assume $(c_2/p_2) < (\mathbf{c_1}/\mathbf{p_1})$, and consider any actor of discount factor $\delta < \hat{\delta}_2(\theta)$.

The actor plays D_1 and D_2 (or doesn't play) throughout the game. Her reputation can thus take three values during her life: $\mathcal{D}_1$, $\mathcal{D}_2$, and $\emptyset$. For any $t \geq 0$, we denote by $\pi^L_{\mathcal{D}_1}(t)$, $\pi^L_{\mathcal{D}_2}(t)$ and $\pi^L_\emptyset(t)$ the probability that this low patience actor is in state $\mathcal{D}_1$, $\mathcal{D}_2$, and $\emptyset$, respectively. By assumption, $\pi^L_{\mathcal{D}_1}(0) = \pi^L_{\mathcal{D}_2}(0) = 0$, and $\pi^L_\emptyset(0) = 1$.

Entering into any round $t \geq 1$, the actor has reputation $\mathcal{D}_1$ if and only if: in the previous round $t-1$, (i) the trust game was drawn, with probability q, (ii) she was trusted, with probability 0 if her reputation was $\mathcal{D}_1$ or $\mathcal{D}_2$, and probability θ if her reputation was $\emptyset$, and (iii) she was observed, with probability $\mathbf{p_1}$.

It follows that, for any $t \geq 1$:

$$\pi^L_{\mathcal{D}_1}(t) = q \times [0 \times \pi^L_{\mathcal{D}_1}(t-1) + 0 \times \pi^L_{\mathcal{D}_2}(t-1) + \theta \times \pi^L_\emptyset(t-1)] \times \mathbf{p_1}.$$

Entering into any round $t \geq 1$, the actor has reputation $\mathcal{D}_2$ if and only if: (i) the institution game was drawn, with probability $1-q$, and (ii) she was observed, with probability p_2. It follows that, for all $t \geq 1$:

$$\pi^L_{\mathcal{D}_2}(t) = (1-q) \times p_2.$$

These equations show that the actor's reputation follows a Markov process, as, for any $t \geq 1$, $\pi^L_{\mathcal{C}_1}(t)$, $\pi^L_{\mathcal{D}_2}(t)$ and $\pi^L_\emptyset(t) = 1 - \pi^L_{\mathcal{D}_1}(t) - \pi^L_{\mathcal{D}_2}(t)$ each only depend on the probabilities for round $t-1$.

Using both of the above equations, the steady state probabilities $\pi^L_{\mathcal{D}_1}, \pi^L_{\mathcal{D}_2}$, and $\pi^L_\emptyset$ must verify:

$$\begin{aligned}\pi^L_{\mathcal{D}_1} &= q\theta\pi^L_\emptyset\mathbf{p_1},\\ \pi^L_{\mathcal{D}_2} &= (1-q)p_2,\\ \pi^L_\emptyset &= 1 - \pi^L_{\mathcal{D}_1} - \pi^L_{\mathcal{D}_2}.\end{aligned}$$

Replacing in the first equation, we deduce:

$$\pi^L_{\mathcal{D}_1} = q\theta(1 - \pi^L_{\mathcal{D}_1} - \pi^L_{\mathcal{D}_2})\mathbf{p_1},$$

which, re-arranging, yields:

$$\pi^L_{\mathcal{D}_1}(1 + q\theta\mathbf{p_1}) = q\theta(1 - \pi^L_{\mathcal{D}_2})\mathbf{p_1},$$

and, re-arranging once more:

$$\pi^L_{\mathcal{D}_1} = q\theta\mathbf{p_1}\frac{1 - \pi^L_{\mathcal{D}_2}}{1 + q\theta\mathbf{p_1}}.$$

Using $\pi^L_{D_2} = (1-q)p_2$, we deduce all three proposed equations. □

4.7 Long-run level of cooperation

We deduce the long-run level of cooperation $\overline{LC}$, defined as the average fraction of actors who, when facing a trust game in the steady state, are first trusted by the chooser they are assigned, and then reciprocate that trust. The long-run level of cooperation is the main output we rely on in our main document and in our numerical solution, in section 7.

Lemma 4.7: Long-run level of cooperation

Assume that choosers play $\sigma_{ch}^{inst,\theta}$, and take $\sigma = (\sigma_{ch}^{inst,\theta}, \sigma_{ac})$ and μ^{∞} such that (σ, μ^{∞}) is a PBE. Assume also that $(c_2/p_2) < (\mathbf{c_1/p_1})$. Then:

$$\overline{LC} = \mathbb{P}(\delta \geq \hat{\delta}_1(\theta)) \times (\pi_{\mathcal{C}_1}^H + \pi_{\mathcal{C}_2}^H + \theta \times \pi_{\emptyset}^H). \tag{4.19}$$

Proof. Take our strategy profile $\sigma = (\sigma_{ch}^{inst,\theta}, \sigma_{ac}) \in \mathcal{S}$, and μ^{∞} such that (σ, μ^{∞}) is a PBE. We assume $(c_2/p_2) < (\mathbf{c_1/p_1})$. As we have shown in Proposition 4.1, in any given round, actors cooperate if and only if their discount rate is greater than $\hat{\delta}_1(\theta)$. This concerns a proportion $\mathbb{P}(\delta \geq \hat{\delta}_1(\theta))$ of the large actor population, this probability depending only on the distribution of time preferences μ_0 and θ.

The reputation of cooperative actors, whose δ is greater than $\hat{\delta}_1(\theta)$, then alternates between $\mathcal{C}_1$, $\mathcal{C}_2$ and $\emptyset$. In any round, when the trust game is drawn, they are then trusted with probability 1 in the first two cases, and probability θ in the case of an empty reputation.

The steady state of their reputation is given by Lemma 4.4, and the above formula immediately follows. □

5 Institution equilibrium: chooser strategy and domain of existence

In this section, we characterize the domain of existence of the institution equilibrium. Previously, in section 3, we showed that σ_{ch}^* is determined whenever certain actors contribute to the institution, and that the only remaining degree of liberty for choosers is the probability θ that they trust an actor of empty reputation. In section 4, we showed that actors' strategy is then fully determined, and can be described by two thresholds, $\hat{\delta}_1(\theta)$ and $\hat{\delta}_2(\theta)$.
Here, we show how to compute the equilibrium value of θ, and derive the conditions under which choosers do not benefit from deviation from σ_{ch}^*. We deduce the domain of existence of the institution equilibrium.

5.1 Objective

Throughout this section, we consider any value $\theta \in [0, 1]$, and assume that, whatever their reputation, actors cooperate if and only if their discount factor is greater or equal than $\hat{\delta}_1(\theta)$, and contribute if and only if their discount factor is greater or equal than $\hat{\delta}_2(\theta)$, where $\theta \in [0, 1]$. We denote such a strategy by $\sigma_{ac}^{inst,\theta}$. As we just saw, following Propositions 4.1 and 4.2, this is the form that the actor's strategy must take in the institution equilibrium in which the chooser trusts given empty reputation with probability θ, i.e. plays according to $\sigma_{ch}^{inst,\theta}$. In addition, in the previous section, we focused on the case where second-order cooperation is easier than first-order cooperation ($\hat{\delta}_2(\theta) < \hat{\delta}_1(\theta) \iff (c_2/p_2) < (\mathbf{c_1/p_1})$)—we continue to focus on this case here, which is the case of interest for the accompanying article.

In other words, we have shown that the institution equilibrium must be a strategy profile of the form $\sigma^{\theta} \equiv (\sigma_{ch}^{inst,\theta}, \sigma_{ac}^{inst,\theta}) \in \mathcal{S}$, where $\theta \in [0, 1]$. In this section, we characterize the domain of existence of the institution equilibrium in the case of interest, by showing how to compute the equilibrium value of θ (Proposition 5.1), thus specifying the strategy profile. We then derive the conditions under which this strategy profile—the institution equilibrium—is a PBE (Proposition 5.2).

5.2 Predictive value of reputation in the steady state

To compute the equilibrium value of θ, and derive the conditions under choosers do not benefit from deviation from σ_{ch}^*, we need to calculate the expected payoff of trusting an actor of any reputation R, which we denote by $u_T(R, \theta)$.

Using the notations introduced in section 3, this payoff is equal to:

$$u_T(R, \theta) = -k + \mathbb{P}_{ch}(C_1 \mid R) \times b.$$

To derive the domain of existence of the institution equilibrium, we thus only need to calculate the probability that an actor of any reputation R will cooperate if trusted, from the point of view of choosers. This probability depends on choosers' posterior beliefs given R, although as we show below, we do not need to precisely derive $\mu^\infty(. \mid R)$. In Lemma 5.1, we compute the probability that an actor of reputation R cooperates in the steady state $\mathbb{P}^\infty(C_1 \mid R)$, which is equal to $\mathbb{P}_{ch}(C_1 \mid R)$ as soon as the Bayesian inference criterion applies (see section 3.2). In Lemma 5.2, we show that this criterion applies as soon as $\hat{\delta}_1(\theta) < 1$, guaranteeing a positive fraction of cooperative actors.

Lemma 5.1: Probability that an actor of reputation R cooperates in the steady state

Assume that players play according to a strategy profile σ^θ, such that $\hat{\delta}_1(\theta) < 1$, and that $(c_2/p_2) < (\mathbf{c_1/p_1})$. In the steady state, the probability that an actor of non-empty reputation R cooperates is a function of these thresholds, as well as the distribution of actor discount factors μ_0. We have:

$$\begin{aligned}
\mathbb{P}^\infty(C_1 \mid \mathcal{C}_1) &= 1, \\
\mathbb{P}^\infty(C_1 \mid \mathcal{D}_1) &= 0, \\
\mathbb{P}^\infty(C_1 \mid \mathcal{C}_2) &= \mathbb{P}(\delta \geq \hat{\delta}_1(\theta) \mid \delta \geq \hat{\delta}_2(\theta)), \\
\mathbb{P}^\infty(C_1 \mid \mathcal{D}_2) &= 0.
\end{aligned}$$

In contrast, the probability that an actor of empty reputation $\emptyset$ cooperates also depends on the steady state probabilities of empty reputation for high, medium and low patience actors, as defined in Lemmas 4.4-4.6. We have:

$$\mathbb{P}^\infty(C_1 \mid \emptyset) = \frac{\mathbb{P}(\delta \geq \hat{\delta}_1(\theta))\pi_\emptyset^H}{\mathbb{P}(\delta \geq \hat{\delta}_1(\theta))\pi_\emptyset^H + \mathbb{P}(\hat{\delta}_1(\theta) > \delta \geq \hat{\delta}_2(\theta))\pi_\emptyset^M + \mathbb{P}(\hat{\delta}_2(\theta) > \delta)\pi_\emptyset^L}. \tag{5.1}$$

Proof. Take $\theta \in [0, 1]$, and consider the strategy profile $\sigma^\theta = (\sigma_{ch}^{inst,\theta}, \sigma_{ac}^{inst,\theta})$, assuming that $\hat{\delta}_1(\theta) < 1$ and $(c_2/p_2) < (\mathbf{c_1/p_1})$. Second-order cooperation is then easier than first-order cooperation ($\hat{\delta}_2(\theta) < \hat{\delta}_2(\theta)$).

Following Lemmas 4.4-4.6, there are three cases for actors: high patience actors, who cooperate and contribute; medium patience actors, who defect and contribute; and low patience actors, who defect and free-ride. Since μ_0 is of full support and $n \gg 1$, there is a positive fraction of actors for each case—for instance, the fraction of high patience actors is equal to:

$$\mathbb{P}(\delta \geq \hat{\delta}_1(\theta)) = \int_{\hat{\delta}_1(\theta)}^1 \mu_0(x)dx.$$

Since there is a positive fraction of high, medium and low patience actors, every reputation R is attained with positive probability in the steady state, and we can define every corresponding conditional probability $\mathbb{P}^\infty(C_1 \mid R)$. Using the notations from these lemmas, the probability that an actor has empty reputation in the steady state is equal to:

$$\mathbb{P}^\infty(\emptyset) = \mathbb{P}(\delta \geq \hat{\delta}_1(\theta)) \times \pi_\emptyset^H + \mathbb{P}(\hat{\delta}_1(\theta) > \delta \geq \hat{\delta}_2(\theta)) \times \pi_\emptyset^M + \mathbb{P}(\hat{\delta}_2(\theta) > \delta) \times \pi_\emptyset^H.$$

Only high patience actors cooperate. The probability that an actor is cooperative and has empty reputation in the steady state is positive, and equal to:

$$\mathbb{P}^\infty(\emptyset \cap \delta \geq \hat{\delta}_1(\theta)) = \mathbb{P}(\delta \geq \hat{\delta}_1(\theta)) \times \pi_\emptyset^H.$$

Dividing, we deduce $\mathbb{P}^\infty(C_1 \mid \emptyset)$, proving condition (5.1).

In contrast, the other four conditions are simpler to prove, and yield simpler expressions. Since actor strategy is stationary, the probability that an actor of reputation $\mathcal{C}_1$ will cooperate if trusted is immediately equal to 1—a positive fraction of high patience actors cooperate, and they are the only ones that reach reputation $\mathcal{C}_1$ with positive probability in the steady state. When an actor has reputation $\mathcal{C}_1$ in the steady state, she must be of high patience, and will therefore cooperate again if trusted:

$$\mathbb{P}^\infty(C_1 \mid \mathcal{C}_1) = 1.$$

Conversely, we obtain $\mathbb{P}^{\infty}(C_1 \mid \mathcal{D}_1) = 0$. Since $\hat{\delta}_2(\theta) < \hat{\delta}_2(\theta)$, we also obtain $\mathbb{P}^{\infty}(C_1 \mid \mathcal{D}_2) = 0$—there are no actors who cooperate and free-ride.

Finally, every medium and high patience actor is equally likely to attain reputation $\mathcal{C}_2$ in the steady state. This depends only on them drawing the institution game and being observed—thus, we have:

$$\pi^H_{\mathcal{C}_2} = \pi^M_{\mathcal{C}_2} = (1-q)p_2.$$

.

By using Lemmas 4.4-4.6 to compute $\mathbb{P}^{\infty}(C_1 \mid \mathcal{C}_2)$ as we computed $\mathbb{P}^{\infty}(C_1 \mid \emptyset)$, these steady state probabilities simplify, and we obtain:

$$\mathbb{P}^{\infty}(C_1 \mid \mathcal{C}_2) = \mathbb{P}(\delta \geq \hat{\delta}_1(\theta) \mid \delta \geq \hat{\delta}_2(\theta)).$$

□

Lemma 5.2: Chooser posterior beliefs and conditional probabilities

Assume that players play according to a strategy profile σ^θ, such that $\hat{\delta}_1(\theta) < 1$, and that $(c_2/p_2) < (\mathbf{c_1}/\mathbf{p_1})$. In a PBE $(\sigma^\theta, \mu^\infty)$, choosers' posterior beliefs are uniquely determined by Bayesian inference, as a function of μ_0, θ and the parameters. In addition, for any R, we have:

$$\mathbb{P}_{ch}(C_1 \mid R) = \mathbb{P}^{\infty}(C_1 \mid R). \tag{5.2}$$

Proof. This immediately follows from our assumptions, as notably explained in section 3.2. We assume that choosers form beliefs based on the steady state of actors' reputation. When actors play according to a strategy profile σ^θ with $\hat{\delta}_2(\theta) < \hat{\delta}_1(\theta) < 1$, as is the case here, every reputation is attained with positive probability in the steady state.

In a PBE, the Bayesian criterion then applies. Choosers' (long-run) posterior beliefs μ^∞ given any non-empty reputation are then uniquely determined as a function of μ_0 and the thresholds $\hat{\delta}_1(\theta)$ and $\hat{\delta}_2(\theta)$.

To begin, given any actor of reputation $\mathcal{C}_1$, her assigned chooser (who is the one that observes this reputation) infers that the actor's discount factor is greater than or equal to $\hat{\delta}_1(\theta)$, and nothing more—since every high patience actor is equally likely to attain cooperative reputation. In other words, for any δ, his posterior beliefs given $\mathcal{C}_1$ are given by:

$$\mu^\infty(\delta \mid C_1) = \frac{\mu_0(\delta) \times \mathbb{1}_{[\hat{\delta}_1(\theta),1]}(\delta)}{\int_{\hat{\delta}_1(\theta)}^1 \mu_0(x)dx},$$

where $\int_{\hat{\delta}_1(\theta)}^1 \mu_0(x)dx$ is the probability of high patience actors, which we previously noted $\mathbb{P}(\delta \geq \hat{\delta}_1(\theta))$ (but δ is a variable in the expression above). To continue, given any actor of reputation $\mathcal{C}_2$, her assigned chooser infers that the actor's discount factor is greater than or equal to $\hat{\delta}_2(\theta)$, and nothing more—since every high and medium actor is equally likely to attain cooperative reputation, as we saw in the proof above. For any δ, his posterior beliefs given $\mathcal{C}_2$ are given by:

$$\mu^\infty(\delta \mid C_2) = \frac{\mu_0(\delta) \times \mathbb{1}_{[\hat{\delta}_2(\theta),1]}(\delta)}{\int_{\hat{\delta}_2(\theta)}^1 \mu_0(x)dx}.$$

Only low patience actors can attain reputation $\mathcal{D}_2$, and do so with equal chance; we similarly deduce:

$$\mu^\infty(\delta \mid D_2) = \frac{\mu_0(\delta) \times \mathbb{1}_{[0,\hat{\delta}_2(\theta)[}(\delta)}{\int_0^{\hat{\delta}_2(\theta)} \mu_0(x)dx}.$$

Computing choosers' posteriors given $\mathcal{D}_1$ and $\emptyset$ is trickier. As we've seen, every type of actor can attain the empty reputation, and does so with positive probability. Choosers' posterior beliefs given $\emptyset$ will then depend on μ_0 and the thresholds, as well as the long-run probabilities of empty reputation $\pi^H_\emptyset$, $\pi^M_\emptyset$ and $\pi^L_\emptyset$. Similarly, both low and medium patience actors can attain reputation $\mathcal{D}_1$, but they do so with different probabilities $\pi^L_{\mathcal{D}_1}$ and $\pi^L_{\mathcal{D}_2}$—medium patience are more likely to be trusted, because they contribute, and therefore more likely to reach this reputation.

In any case, whatever the expression of these posteriors, we must have:

$$\mathbb{P}_{ch}(C_1 \mid R) = \mathbb{P}^{\infty}(C_1 \mid R). \tag{5.2}$$

In other words, we do not need to derive a precise expression for these posteriors; given our assumptions, we must have $\mathbb{P}_{ch}(C_1 \mid \mathcal{D}_1) = 0$ (low and medium patience actors defect), while $\mathbb{P}_{ch}(C_1 \mid \emptyset)$ can be deduced using condition (5.1). □

Note that we do not need to limit ourselves to the steady state for non-empty reputations. Actors play a stationary strategy, and, when $\hat{\delta}_2(\theta) < \hat{\delta}_1(\theta) < 1$, all behaviors are represented, and every reputation is attained with positive probability—for instance, medium patience actors attain reputation $\mathcal{D}_1$ any time they draw the institution game and are observed playing C_2 in the previous round, and subsequently draw a trust game and are observed playing D_1. In a PBE, the Bayesian inference criterion must then apply given any reputation R during the transient state—i.e., given any history—and any reasonable application of this criterion will lead to choosers' forming the same posterior beliefs as they do here given $\mathcal{C}_1$, $\mathcal{C}_2$ or $\mathcal{D}_2$, and to us deriving the same conditional probabilities $\mathbb{P}_{ch}(C_1 \mid R)$ for every non-empty reputation (even $R = \mathcal{D}_1$). To have a precise distribution of reputations to apply, we have nevertheless chosen to focus on the distribution obtained in the steady state for non-empty reputations as well as the empty reputation.

Note also that, when, in contrast to the assumptions of these two lemmas, $\hat{\delta}_1(\theta) \geq 1$, there are no would-be cooperators. The Bayesian inference criterion then does not apply for actors of reputation $\mathcal{C}_1$—technically, any posteriors beliefs are possible in a PBE. This does not change anything to the final result though, since whatever the posteriors μ, (σ^θ, μ) cannot be a PBE. Choosers always benefit from deviation to distrusting given $\mathcal{C}_1$, since every actor is a defector—whatever the posterior beliefs we assign them, we will have $\mathbb{P}_{ch}(C_1 \mid \mathcal{C}_1) = 0$ in this case.

5.3 Long-run chooser payoff

In our numerical results, in section 7, we rely on the normalized actor payoff $\overline{U}_\delta$, calculated in Lemma 4.3, as well as the long-run chooser payoff u^∞, defined as the payoff of any chooser in the steady state, who is paired with a randomly assigned actor in the trust game. We calculate u^∞ below.

Lemma 5.3: Long-run chooser payoff

Assume that players play according to a strategy profile σ^θ, such that $\hat{\delta}_1(\theta) < 1$, and that $(c_2/p_2) < (\mathbf{c_1/p_1})$. The long-run probability of each reputation is obtained using the formulas defined in Lemmas 4.4-4.6. We have:

$$\begin{aligned}
\mathbb{P}^\infty(\emptyset) &= \mathbb{P}(\delta \geq \hat{\delta}_1(\theta)) \times \pi^H_\emptyset + \mathbb{P}(\hat{\delta}_1(\theta) > \delta \geq \hat{\delta}_2(\theta)) \times \pi^M_\emptyset + \mathbb{P}(\hat{\delta}_2(\theta) > \delta) \times \pi^L_\emptyset, \\
\mathbb{P}^\infty(\mathcal{C}_1) &= \mathbb{P}(\delta \geq \hat{\delta}_1(\theta)) \times \pi^H_{\mathcal{C}_1}, \\
\mathbb{P}^\infty(\mathcal{D}_1) &= \mathbb{P}(\hat{\delta}_1(\theta) > \delta \geq \hat{\delta}_2(\theta)) \times \pi^M_{\mathcal{D}_1} + \mathbb{P}(\hat{\delta}_2(\theta) > \delta) \times \pi^L_{\mathcal{D}_1}, \\
\mathbb{P}^\infty(\mathcal{C}_2) &= \mathbb{P}(\delta \geq \hat{\delta}_1(\theta)) \times \pi^H_{\mathcal{C}_2} + \mathbb{P}(\hat{\delta}_1(\theta) > \delta \geq \hat{\delta}_2(\theta)) \times \pi^M_{\mathcal{C}_2} = \mathbb{P}(\delta \geq \hat{\delta}_2(\theta)) \times (1-q)p_2, \\
\mathbb{P}^\infty(\mathcal{D}_2) &= \mathbb{P}(\hat{\delta}_2(\theta) > \delta) \times \pi^L_{\mathcal{D}_2}.
\end{aligned}$$

In a PBE $(\sigma^\theta, \mu^\infty)$, the long-run chooser payoff is then equal to:

$$u^\infty \equiv \theta \times \mathbb{P}^\infty(\emptyset)(-k + \mathbb{P}^\infty(C_1 \mid \emptyset)b) + \mathbb{P}^\infty(\mathcal{C}_1)(-k+b) + \mathbb{P}^\infty(\mathcal{C}_2)(-k + \mathbb{P}(\delta \geq \hat{\delta}_1(\theta) \mid \delta \geq \hat{\delta}_2(\theta))b). \quad (5.3)$$

Proof. Take $\theta \in [0,1]$, and consider the strategy profile $\sigma^\theta = (\sigma^{inst,\theta}_{ch}, \sigma^{inst,\theta}_{ac})$. We assume that $\hat{\delta}_2(\theta) < \hat{\delta}_1(\theta) < 1$.

In the proof Lemma 5.1, we used the formulas from Lemmas 4.4-4.6 to calculate $\mathbb{P}^\infty(\emptyset)$. We can similarly calculate the probability of each reputation, taking into account which actors are able to achieve that reputation (e.g., only high patience actors can achieve reputation $\mathcal{C}_1$). This yields the five conditions above.

We deduce the long-run payoff of a chooser who faces a randomly selected actor in the trust game u^∞ by taking into account that the chooser trusts with probability θ given $\emptyset$, and with probability 1 given $\mathcal{C}_1$ and $\mathcal{C}_2$; and also by taking into account the probabilities that the actor reciprocates given each of these reputations in the steady state, all three of which we have already calculated. □

5.4 Equilibrium value of θ

We have calculated $\mathbb{P}_{ch}(C_1 \mid \emptyset)$ in the case of interest—the institution equilibrium, given that second-order cooperation remains easier than first-order cooperation. Since the institution equilibrium is perfect, choosers optimize based on $\mathbb{P}_{ch}(C_1 \mid \emptyset)$, as we recall in Lemma 5.4. We deduce an algorithm for determining the equilibrium value of θ, in Proposition 5.1.

Note that the algorithm can be under-specified: there could be two values $\theta \in (0,1)$ such that $u_T(\emptyset, \theta) = 0$. In the numerical solution below, in section 7, we consider specific parameter regions (namely, those for which $\mathbf{p}_1$ is

not too close to 1), in which payoff of a chooser who trusts an actor of empty reputation in a candidate institution equilibrium σ^θ is a strictly decreasing function of θ. In those cases, the algorithm below yields a unique value θ^*, and therefore a unique candidate profile for the institution equilibrium.

Lemma 5.4: Payoff of trusting an actor of empty reputation

Assume that players play according to a strategy profile σ^θ. In a PBE (σ, μ^∞),

$$\begin{aligned} \theta = 0 &\implies u_T(\emptyset, \theta) \le 0, \\ \theta = 1 &\implies u_T(\emptyset, \theta) \ge 0, \\ \theta \in (0,1) &\implies u_T(\emptyset, \theta) = 0. \end{aligned}$$

Proof. In a PBE, there cannot be any beneficial deviations for choosers who face an actor of empty reputation. If $\theta = 0$, these choosers distrust, obtaining payoff 0—deviating to trusting cannot be beneficial, from which we deduce that we must have $u_T(\emptyset, \theta) \le 0$. Similarly, if $\theta = 1$, deviation to distrusting cannot be beneficial ($u_T(\emptyset, \theta) \ge 0$), and otherwise, choosers must be indifferent between both options ($u_T(\emptyset, \theta) = 0$). □

Proposition 5.1: Equilibrium value of θ

An algorithm to determine a candidate value for θ, and therefore a candidate institution equilibrium when second-order cooperation is cheaper than first-order cooperation, is to take $\theta = \theta^*$, where θ^* is defined by:

$$\theta^* \equiv \begin{cases} 0 & \text{if } u_T(\emptyset, 0) \le 0, \\ 1 & \text{if } u_T(\emptyset, 1) \ge 0, \\ t & \text{such that } u_T(\emptyset, t) = 0. \end{cases} \tag{5.4}$$

Proof. Take $\theta \in [0,1]$, and consider the strategy profile $\sigma^\theta = (\sigma_{ch}^{inst,\theta}, \sigma_{ac}^{inst,\theta})$. We assume that $(c_2/p_2) < (\mathbf{c_1}/\mathbf{p_1})$, and that there exists μ^∞ such that $(\sigma^\theta, \mu^\infty)$ is a PBE. As we have seen, we must then have $\hat{\delta}_1(\theta) < 1$ (otherwise, choosers benefit from distrusting every actor, including those who have cooperative reputation $\mathcal{C}_1$). The expected payoff of trusting an actor of empty reputation is then equal to:

$$u_T(\emptyset, \theta) = -k + \mathbb{P}_{ch}(C_1 \mid \emptyset) \times b,$$

where, following Lemmas 5.1-5.2, the probability that such an actor cooperates is equal to:

$$\mathbb{P}_{ch}(C_1 \mid \emptyset) = \frac{\mathbb{P}(\delta \ge \hat{\delta}_1(\theta))\pi_\emptyset^H}{\mathbb{P}(\delta \ge \hat{\delta}_1(\theta))\pi_\emptyset^H + \mathbb{P}(\hat{\delta}_1(\theta) > \delta \ge \hat{\delta}_2(\theta))\pi_\emptyset^M + \mathbb{P}(\hat{\delta}_2(\theta) > \delta)\pi_\emptyset^L}.$$

This shows that $u_T(\emptyset, \theta)$ can be calculated for any θ (we use these formulas in our numerical solution).

Our algorithm has three steps. First: test if $u_T(\emptyset, 0) \le 0$, and if so, take $\theta^* = 0$. When the expected payoff of trusting an actor of empty reputation is negative or null given that actors play according to σ_{ac}^0, we consider the strategy profile σ^0 in which choosers distrust such actors.

Second, if $u_T(\emptyset, 0) > 0$, meaning that the equilibrium value of θ cannot be equal to 0, test whether $u_T(\emptyset, 1) \ge 0$; if so, take $\theta^* = 1$.

Third, if neither of those conditions are true, then the only possibility is that choosers mix given $\emptyset$, and therefore that they are indifferent between trusting and distrusting. In that case, the algorithm yields a value that cancels out the payoff of trusting an actor of empty reputation, without specifying which one (one such value must exist by continuity).

In other words, the algorithm always yields a candidate value $\theta^* \in [0,1]$, and a candidate strategy profile σ^{θ^*} in which choosers do not benefit from deviation given $\emptyset$—meaning that all that is left to do is to see if there are beneficial deviations given non-empty reputations. The algorithm does not specify which value to take when neither 0 nor 1 are possible, but in the numerical solution this concern is moot, as we show that $u_T(\emptyset, \theta)$ is a strictly decreasing function of θ (this also means that the order of our algorithm, which gives a slight edge to taking $\theta = 0$, does not have any impact on its final output). □

5.5 Domain of existence of the institution equilibrium

Proposition 5.2: Domain of existence of the institution equilibrium

If $(c_2/p_2) < (\mathbf{c_1/p_1})$, there exists a PBE $(\sigma^{\theta^*}, \mu^\infty)$, where θ^* is defined as in Proposition 5.1, and μ^∞ derived as in Lemma 5.2, if and only if:

$$\hat{\delta}_2(\theta^*) < 1, \tag{5.5}$$

$$\mathbb{P}(\delta \geq \hat{\delta}_1(\theta^*) \mid \delta \geq \hat{\delta}_2(\theta^*)) \geq \frac{k}{b}. \tag{5.6}$$

We obtain an institution equilibrium when second-order cooperation is a sufficiently good predictor of first-order cooperation.

Proof. Consider the strategy profile $\sigma^{\theta^*} = (\sigma_{ch}^{inst,\theta^*}, \sigma_{ac}^{inst,\theta^*})$, where θ^* is defined as in Proposition 5.1.

We begin by noting that a necessary condition is given by:

$$\hat{\delta}_1(\theta^*) < 1.$$

Indeed, as we have seen, if $\hat{\delta}_1(\theta^*) \geq 1$, every actor defects, and choosers benefit from distrusting all actors, and therefore from deviation to distrusting actors who have cooperative reputation $\mathcal{C}_1$. This means that condition (5.5) must be true, since second-order cooperation is assumed to be easier than first-order cooperation $(c_2/p_2 < (\mathbf{c_1/p_1}))$.

This also means that we can use the previous lemmas to calculate the payoff of trusting an actor. In particular, the payoff of trusting an actor that was previously observed contributing to the institution is equal to:

$$u_T(\mathcal{C}_2, \theta^*) = -k + \mathbb{P}_{ch}(C_1 \mid \mathcal{C}_2) \times b,$$

which, following Lemmas 5.1-5.2, is equal to:

$$u_T(\mathcal{C}_2, \theta^*) = -k + \mathbb{P}(\delta \geq \hat{\delta}_1(\theta^*) \mid \delta \geq \hat{\delta}_2(\theta^*)) \times b.$$

Since the chooser trusts in such a case, we must have $u_T(\mathcal{C}_2, \theta^*) \geq 0$, from which we deduce that condition (5.6) is necessary.

We prove that conditions (5.5-5.6) are together sufficient, given that we also have $(c_2/p_2) < (\mathbf{c_1/p_1})$, by checking that the chooser does not have any beneficial deviations when they hold. In such a case, we must have $\hat{\delta}_1(\theta^*) < 1$ — if second-order cooperation is a sufficiently good predictor of first-order cooperation, than there must be something to predict; first-order cooperation must be possible. We can once again use Lemmas 5.1-5.2 to derive choosers' posterior beliefs μ^∞, and the equilibrium conditional probabilities $\mathbb{P}_{ch}(C_1 \mid R)$ for any R.

Since $\mathbb{P}_{ch}(C_1 \mid \mathcal{C}_1) = 1$ and $\mathbb{P}_{ch}(C_1 \mid \mathcal{D}_1) = 0$, choosers do not benefit from deviation to distrusting actors who have a cooperative reputation, and do not benefit from deviation to trusting actors whose reputation indicates they previously defected. Reputation then incentivizes first-order cooperation.

Since $\mathbb{P}_{ch}(C_1 \mid \mathcal{C}_2) = \mathbb{P}(\delta \geq \hat{\delta}_1(\theta^*) \mid \delta \geq \hat{\delta}_2(\theta^*)) \geq k/b$ and $\mathbb{P}_{ch}(C_1 \mid \mathcal{D}_2) = 0$, choosers do not benefit from deviation to distrusting actors who have previously been observed contributing to the institution, and do not benefit from deviation to trusting actor whose reputation indicates that they previously free-rode. Reputation then incentives second-order cooperation.

By construction, choosers do not benefit from deviation from their strategy given an actor of empty reputation (Proposition 5.1), and, as we have seen, actors' best response is then given by $\sigma_{ac}^{\theta^*}$. This proves that $(\sigma^{\theta^*}, \mu^\infty)$ is a PBE. □

6 Baseline equilibrium

In this section, we establish a baseline for comparison with the results of the institution equilibrium. To do so, we take $p_2 = 0$, in contrast to before. We show that there then exists a unique PBE in which certain actors cooperate—the baseline equilibrium—which we characterize.

6.1 Objective

In this section, we establish a baseline for comparison with the results of the institution equilibrium. To do so, we 'turn off' information coming from the institution game, by assuming that:

$$p_2 = 0. \tag{6.1}$$

In such a case, every actor will free-ride when given the opportunity to do so, since second-order cooperation is costly and can no longer be observed. The institution is then moot, and will never produce incentives in a PBE. We restrict the possible reputations of the actor to $\mathcal{R}^b \equiv \{\mathcal{C}_1, \mathcal{D}_1, \emptyset\}$, omitting the reputations that concern the institution game, and correspondingly restrict the set of strategies.

We look for a cooperative equilibrium; that is, an equilibrium in which we can expect some actors to cooperate if trusted. In such a case, as we saw in section 3.3.5, reputation must incentivize cooperation. Choosers must trust actors whose reputation is $\mathcal{C}_1$ and distrust actors whose reputation is $\mathcal{D}_1$. In other words, there is once again only one degree of liberty left for choosers, the probability $\theta \in [0, 1]$ that they trust an actor of empty reputation.

We denote the corresponding chooser strategy by $\sigma_{ch}^{base,\theta}$. We denote actors' strategy by σ_{ac}, which is now restricted to a map from $\Delta \times \mathcal{R}^b$ to $\{C_1, D_1\}$ (we do not look at actor's behavior in the institution game, where we know they will free-ride).

When the strategy profile $\sigma^\theta \equiv (\sigma_{ch}^{base,\theta}, \sigma_{ac})$ is a PBE, we refer to it as the **baseline equilibrium**. Below, we characterize the baseline equilibrium, by following the same steps as in sections 4-5 for the institution equilibrium. We notably show that this equilibrium is unique, which is why refer to it as *the* baseline equilibrium, rather than a baseline equilibrium.

Note that we use the superscript b for the main characteristics of the baseline equilibrium—the threshold discount factor separating cooperators from defectors, and the equilibrium value of θ. We do not do this for other values that will be useful in our numerical calculations to not overcharge these notations (e.g., the reputational benefit, or players' payoffs).

6.2 Threshold discount factor for cooperation

We show that in the baseline equilibrium, sufficiently patient actors cooperate, and sufficiently impatient actors defect—the indifference point being captured by a single threshold value $\hat{\delta}^b(\theta)$.

Proposition 6.1: Threshold discount factor for cooperation in the baseline

Assume that choosers play $\sigma_{ch}^{base,\theta}$, and take $\sigma = (\sigma_{ch}^{base,\theta}, \sigma_{ac})$ and μ^∞ such that (σ, μ^∞) is a PBE. Sufficiently patient ($\delta \geq \hat{\delta}^b(\theta)$) actors then always reciprocate the trust of choosers, and sufficiently impatient ($\delta < \hat{\delta}^b(\theta)$) actors always cheat—the value of the threshold separating reciprocators from cheaters being given by:

$$\hat{\delta}^b(\theta) \equiv \frac{c_1}{p_1 q(r - \theta c_1)}. \tag{6.2}$$

Proof. The proof is analogous to Proposition 4.1. Using the fact that the actor's strategy is stationary, we similarly partition Δ into two intervals I_{D_1} and I_{C_1}, over which the actor respectively always cheats or always cooperates, whatever her reputation. We can then calculate the reputational benefit R_δ in this baseline equilibrium in the same manner than in Lemma 4.2, by plugging in $\beta = \gamma = \pi_1 = 0$ (since the actor never contributes) to our previous expression. We obtain:

$$R_\delta = \begin{cases} \frac{qr}{1+q\delta p_1 \theta} & \text{if } \delta \in I_{D_1}, \\ \frac{q(r-c_1)}{1-q\delta p_1(1-\theta)} & \text{if } \delta \in I_{C_1}. \end{cases} \tag{6.3}$$

Using the same reasoning as in Proposition 4.1, we deduce the proposed value for $\hat{\delta}_1^b(\theta)$. □

Similarly to before, we refer to $\hat{\delta}_1^b(\theta)$ as the **difficulty of cooperation in the baseline equilibrium for** θ. We also call $\hat{\delta}^b(0) = \max\{\hat{\delta}_1^b(\theta), \theta \in [0, 1]\}$ the **difficulty of cooperation**, and denote it by δ^b. The difficulty of cooperation $\delta^b = (c_1/p_1 qr)$ is a function of our parameters, and characterizes the repeated game as a whole; in contrast to the three other 'difficulties' we have defined, it does not depend on choosers' strategy. We will use the intrinsic difficulty of cooperation δ^b in our graphical representations, in section 7 (y-axis of each plot).

6.3 Actor payoffs, long-run reputation and level of cooperation

We calculate the normalized payoff of any actor in the baseline equilibrium.

Lemma 6.1: Normalized actor payoff

Assume that choosers play $\sigma_{ch}^{base,\theta}$, and take $\sigma = (\sigma_{ch}^{base,\theta}, \sigma_{ac})$ and μ^∞ such that (σ, μ^∞) is a PBE.
For any $\delta \in \Delta$, we have:

$$\overline{U}_\delta = \theta \times R_\delta. \tag{6.4}$$

Proof. Since $p_2 = 0$, we have: $U_\delta^B = \delta \times U_\delta^\emptyset$ (these quantities are defined analogously to before). When in bad standing, an actor always achieves null standing in the next round—she is either distrusted in the trust game, or faces the institution game and is not observed.

Using $U_\delta^\emptyset - U_\delta^B = \theta R_\delta$ (analogous to condition 4.3), we deduce:

$$U_\delta^\theta - \theta R_\delta^b = \delta \times U_\delta^\theta.$$

Re-arranging, this is equivalent to:

$$(1-\delta) \times U_\delta^\theta = \theta R_\delta.$$

We recognize the normalized payoff of a generic actor of discount factor δ on the left of this condition. □

In the baseline equilibrium, every actor's reputation again follows a Markov process—with two cases of interest (high and low patience) this time instead of three.

Lemma 6.2: Long-run reputation for a high patience actor

Assume that choosers play $\sigma_{ch}^{base,\theta}$, and take $\sigma = (\sigma_{ch}^{base,\theta}, \sigma_{ac})$ and μ^∞ such that (σ, μ^∞) is a PBE.
For any $\delta \geq \hat{\delta}_1^b(\theta)$, the reputation of an actor of discount factor δ follows a Markov process with two states: $\mathcal{C}_1$ and $\emptyset$. The steady state $\pi^H \equiv (\pi_{\mathcal{C}_1}^H, \pi_\emptyset^H)$ of the actor's reputation is given by:

$$\pi_{\mathcal{C}_1}^H \equiv \frac{q\theta p_1}{1 - q(1-\theta)p_1}, \tag{6.5}$$

$$\pi_\emptyset^H \equiv 1 - \frac{q\theta p_1}{1 - q(1-\theta)p_1}. \tag{6.6}$$

Proof. Take our strategy profile $\sigma = (\sigma_{ch}^{base,\theta}, \sigma_{ac})$, and μ^∞ such that (σ, μ^∞) is a PBE. We consider any actor of discount factor $\delta \geq \hat{\delta}_1^b(\theta)$, assuming such a value exists.

The actor plays C_1 and D_2 (or doesn't play) throughout the game. Since $p_2 = 0$, her reputation can take only two values during her life: $\mathcal{C}_1$ and $\emptyset$. For any $t \geq 0$, we denote by $\pi_{\mathcal{C}_1}^H(t)$ and $\pi_\emptyset^H(t)$ the probability that this high patience actor is in state $\mathcal{C}_1$ and $\emptyset$, respectively. By assumption, $\pi_{\mathcal{C}_1}^H(0) = 0$, and $\pi_\emptyset^H(0) = 1$.

Entering into any round $t \geq 1$, the actor has reputation $\mathcal{C}_1$ if and only if: in the previous round $t-1$, (i) the trust game was drawn, with probability q, (ii) she was trusted, with probability 1 if her reputation was $\mathcal{C}_1$, and probability θ if her reputation was $\emptyset$, and (iii) she was observed, with probability p_1.

It follows that, for any $t \geq 1$:

$$\pi_{\mathcal{C}_1}^H(t) = q \times [1 \times \pi_{\mathcal{C}_1}^H(t-1) + \theta \times \pi_\emptyset^H(t-1)] \times p_1.$$

Using this equation, the steady state probabilities $\pi_{\mathcal{C}_1}^H$ and $\pi_\emptyset^H$ must verify:

$$\begin{aligned} \pi_{\mathcal{C}_1}^H &= q(\pi_{\mathcal{C}_1}^H + \theta\pi_\emptyset^H)p_1, \\ \pi_\emptyset^H &= 1 - \pi_{\mathcal{C}_1}^H. \end{aligned}$$

Replacing in the first equation, we deduce:

$$\begin{aligned} & \pi_{\mathcal{C}_1}^H = q(\pi_{\mathcal{C}_1}^H + \theta(1 - \pi_{\mathcal{C}_1}^H))p_1, \\ \iff \quad & \pi_{\mathcal{C}_1}^H(1 - q(1-\theta)p_1) = q\theta p_1, \\ \iff \quad & \pi_{\mathcal{C}_1}^H = \frac{q\theta p_1}{1 - q(1-\theta)p_1}. \end{aligned}$$

□

Lemma 6.3: Long-run reputation for a low patience actor

Assume that choosers play $\sigma_{ch}^{base,\theta}$, and take $\sigma = (\sigma_{ch}^{base,\theta}, \sigma_{ac})$ and μ^∞ such that (σ, μ^∞) is a PBE.
For any $\delta < \hat{\delta}_1^b(\theta)$, the reputation of an actor of discount factor δ follows a Markov process with two states: $\mathcal{D}_1$ and $\emptyset$. The steady state $\pi^L \equiv (\pi^L_{\mathcal{D}_1}, \pi^L_{\emptyset})$ of the actor's reputation is given by:

$$\pi^L_{\mathcal{D}_1} \equiv \frac{q\theta p_1}{1 + q\theta p_1}, \tag{6.7}$$

$$\pi^L_{\emptyset} \equiv 1 - \frac{q\theta p_1}{1 + q\theta p_1}. \tag{6.8}$$

Proof. Take our strategy profile $\sigma = (\sigma_{ch}^{base,\theta}, \sigma_{ac})$, and μ^∞ such that (σ, μ^∞) is a PBE. We consider any actor of discount factor $\delta < \hat{\delta}_1^b(\theta)$.

The actor plays D_1 and D_2 (or doesn't play) throughout the game. Since $p_2 = 0$, her reputation can take only two values during her life: $\mathcal{D}_1$ and $\emptyset$. For any $t \geq 0$, we denote by $\pi^L_{\mathcal{D}_1}(t)$ and $\pi^L_{\emptyset}(t)$ the probability that this low patience actor is in state $\mathcal{D}_1$ and $\emptyset$, respectively. By assumption, $\pi^L_{\mathcal{D}_1}(0) = 0$, and $\pi^L_{\emptyset}(0) = 1$.

Entering into any round $t \geq 1$, the actor has reputation $\mathcal{D}_1$ if and only if: in the previous round $t-1$, (i) the trust game was drawn, with probability q, (ii) she was trusted, with probability 0 if her reputation was $\mathcal{D}_1$, and probability θ if her reputation was $\emptyset$, and (iii) she was observed, with probability p_1.

It follows that, for any $t \geq 1$:

$$\pi^L_{\mathcal{D}_1}(t) = q \times [0 \times \pi^L_{\mathcal{D}_1}(t-1) + \theta \times \pi^L_{\emptyset}(t-1)] \times p_1.$$

Using this equation, the steady state probabilities $\pi^L_{\mathcal{D}_1}$ and $\pi^L_{\emptyset}$ must verify:

$$\pi^L_{\mathcal{D}_1} = q\theta\pi^L_{\emptyset} p_1,$$
$$\pi^L_{\emptyset} = 1 - \pi^L_{\mathcal{D}_1}.$$

Replacing in the first equation, we deduce:

$$\pi^L_{\mathcal{D}_1} = q\theta(1 - \pi^L_{\mathcal{D}_1})p_1,$$
$$\iff \qquad \pi^L_{\mathcal{D}_1} = \frac{q\theta p_1}{1 + q\theta p_1}.$$

□

Lemma 6.4: Long-run level of cooperation

Assume that choosers play $\sigma_{ch}^{base,\theta}$, and take $\sigma = (\sigma_{ch}^{base,\theta}, \sigma_{ac})$ and μ^∞ such that (σ, μ^∞) is a PBE. The long-run level of cooperation is then given by:

$$\overline{LC} = \mathbb{P}(\delta \geq \hat{\delta}_1^b(\theta)) \times (\pi^H_{\mathcal{C}_1} + \theta\pi^H_{\emptyset}). \tag{6.9}$$

Proof. Immediate, and analogous to lemma 4.7. □

6.4 Chooser inferences and long-run payoff

Similarly to before, we denote the resulting actor strategy by $\sigma_{ac}^{base,\theta}$, and the resulting strategy profile by $\sigma^{b,\theta} \equiv (\sigma_{ch}^{base,\theta}, \sigma_{ac}^{base,\theta})$. We proceed similarly to section 5, with one small caveat: when $\theta = 0$, every actor has empty reputation in the steady state. This prevents us from defining the conditional probability of cooperation in the steady state when $\theta = 0$, but not from defining choosers' beliefs and subjective conditional probabilities.

Lemma 6.5: Probability that an actor of reputation R cooperates in the steady state

Assume that players play according to a strategy profile $\sigma^{b,\theta}$, such that $\hat{\delta}_1^b(\theta) < 1$.
In the steady state, the probability that an actor of non-empty reputation $\mathcal{C}_1$ or $\mathcal{D}_1$ cooperates can be defined if $\theta > 0$, and is then a function of $\hat{\delta}_1^b(\theta)$ and μ_0. If $\theta > 0$, we have:

$$\mathbb{P}(C_1 \mid \mathcal{C}_1) = 1,$$
$$\mathbb{P}(C_1 \mid \mathcal{D}_1) = 0.$$

In contrast, the probability that an actor of empty reputation $\emptyset$ cooperates can also be defined when $\theta = 0$, and also depends on the steady state probability of empty reputation for high and low patience actors, as defined in Lemmas 6.2-6.3. We have:

$$\mathbb{P}^\infty(C_1 \mid \emptyset) = \frac{\mathbb{P}(\delta \geq \hat{\delta}_1^b(\theta))\pi_\emptyset^H}{\mathbb{P}(\delta \geq \hat{\delta}_1^b(\theta))\pi_\emptyset^H + \mathbb{P}(\hat{\delta}_1^b(\theta) > \delta)\pi_\emptyset^L}. \tag{6.10}$$

Proof. The proof is analogous to that of Lemma 5.1, and uses the formulas derived in Lemmas 6.2-6.3. The only difference is that if $\theta = 0$, both non-empty reputations $\mathcal{C}_1$ and $\mathcal{D}_1$ occur with probability 0 in the steady state, even though there is a positive fraction of would-be cooperators ($\hat{\delta}_1^b(\theta) < 1$). Actors then never exit their initial empty reputation along the outcome path—whereas in the institution equilibrium, actors can always obtain reputation $\mathcal{C}_2$ or $\mathcal{D}_2$ via the institution ($p_2 > 0$), meaning that medium and high patience actors are sometimes trusted by choosers along the outcome path even when $\theta = 0$, and can then still attain reputations $\mathcal{D}_1$ and $\mathcal{C}_1$. □

Lemma 6.6: Chooser posterior beliefs and conditional probabilities

Assume that players play according to a strategy profile $\sigma^{b,\theta}$, such that $\hat{\delta}_1^b(\theta) < 1$.
In a PBE $(\sigma^\theta, \mu^\infty)$, choosers' posterior beliefs are uniquely determined by Bayesian inference, as a function of μ_0, θ and the parameters. In addition, we have:

$$\mathbb{P}_{ch}(C_1 \mid \emptyset) = \mathbb{P}^\infty(C_1 \mid \emptyset), \tag{6.11}$$
$$\mathbb{P}_{ch}(C_1 \mid \mathcal{C}_1) = 1, \tag{6.12}$$
$$\mathbb{P}_{ch}(C_1 \mid \mathcal{D}_1) = 0. \tag{6.13}$$

Proof. The proof is analogous to that of Lemma 6.6, and follows from our assumptions about chooser posteriors, explained in section 3.2. The only difference is that we can only define $\mathbb{P}^\infty(C_1 \mid \mathcal{C}_1)$ and $\mathbb{P}^\infty(C_1 \mid \mathcal{D}_1)$ when $\theta > 0$, meaning that, using the previous lemma, we always obtain (6.11), but only prove (6.12) and (6.13) when $\theta > 0$.

If $\theta = 0$, $\mathbb{P}^\infty(\mathcal{C}_1) = \mathbb{P}^\infty(\mathcal{D}_1) = 0$, and we cannot define the conditional probability of cooperation given a non-empty reputation in the steady state, or use the previous lemma. Nevertheless, the Bayesian inference criterion applies, because there are would-be cooperators and would-be cheaters, who can attain either non-empty reputation if they are trusted once, even though this does not occur along the outcome path (which we have used to define the steady state of reputations). Given the steady state distribution of reputations, whereby every individual has empty reputation, there are histories at which any actor can acquire a non-empty reputation: the previous chooser could mistakenly trust the actor when her reputation is $\emptyset$, and the actor could then be observed playing her prescribed action. Since $0 < \hat{\delta}_1^b < 1$, the cooperative reputation $\mathcal{C}_1$ (resp. reputation $\mathcal{D}_1$) is obtained with positive probability once a chooser has made this mistake, for any actor whose discount factor is above or equal to the threshold (resp. below the threshold). Bayes' rule then yields posteriors as in the proof of Lemma 5.2: choosers can infer that such an actor must have a discount factor above or equal to the threshold (resp. below), and nothing more.

Since actor strategy is stationary, both non-empty reputations perfectly predict whether an actor will cooperate again if trusted; in other words, even when $\theta = 0$, we obtain:

$$\mathbb{P}_{ch}(C_1 \mid \mathcal{C}_1) = 1,$$
$$\mathbb{P}_{ch}(C_1 \mid \mathcal{D}_1) = 0.$$

□

Lemma 6.7: Long-run chooser payoff

Assume that players play according to a strategy profile $\sigma^{b,\theta} = (\sigma_{ch}^{base,\theta}, \sigma_{ac}^{base,\theta})$, such that $\hat{\delta}_1^b(\theta) < 1$. The long-run probability of each reputation is obtained using the formulas defined in Lemmas 6.2-6.3. We have:

$$\begin{aligned}\mathbb{P}^\infty(\emptyset) &= \mathbb{P}(\delta \geq \hat{\delta}_1^b(\theta)) \times \pi_\emptyset^H + \mathbb{P}(\hat{\delta}_1^b(\theta) > \delta) \times \pi_\emptyset^L,\\ \mathbb{P}^\infty(\mathcal{C}_1) &= \mathbb{P}(\delta \geq \hat{\delta}_1^b(\theta)) \times \pi_{\mathcal{C}_1}^H,\\ \mathbb{P}^\infty(\mathcal{D}_1) &= \mathbb{P}(\hat{\delta}_1^b(\theta) > \delta) \times \pi_{\mathcal{D}_1}^L.\end{aligned}$$

In a PBE $(\sigma^{b,\theta}, \mu^\infty)$, the long-run chooser payoff is then equal to:

$$u^\infty \equiv \theta \times \mathbb{P}^\infty(\emptyset)(-k + \mathbb{P}^\infty(C_1 \mid \emptyset)b) + \mathbb{P}^\infty(\mathcal{C}_1)(-k + b). \tag{6.14}$$

Proof. The long-run probabilities of each reputational state are calculated similarly to before, by distinguishing between high and low patience actors, and using the formulas defined in Lemmas 6.2-6.3. We immediately deduce the long-run payoff of the chooser. □

6.5 Equilibrium value of θ

We have thus calculated $\mathbb{P}_{ch}(C_1 \mid \emptyset)$ when players play according to $\sigma^{b,\theta}$, allowing us to derive the expected payoff of trusting an actor of empty reputation $u_T(\emptyset, \theta)$ in a candidate for the baseline equilibrium. Using the same algorithm as before, we deduce the equilibrium value of θ in a PBE. Note that, in contrast to before, this algorithm always yields a unique value. This is because $u_T(\emptyset, \theta)$ is this time always a strictly decreasing function of θ.

Proposition 6.2: Equilibrium value of θ in the baseline

An algorithm to determine a unique candidate value for θ, and therefore the unique candidate baseline equilibrium is to take $\theta = \theta^{*,b}$, where $\theta^{*,b}$ is defined by:

$$\theta^{*,b} \equiv \begin{cases} 0 & \text{if } u_T(\emptyset, 0) \leq 0,\\ 1 & \text{if } u_T(\emptyset, 1) \geq 0,\\ t & \text{such that } u_T(\emptyset, t) = 0.\end{cases} \tag{6.15}$$

Proof. Using the same steps as before, we can calculate $u_T(\emptyset, \theta)$ for any θ. We deduce that the algorithm above, which is the same as the one used in the institution equilibrium, will yield at least one candidate value for θ.

In addition, we note that $u_T(\emptyset, \theta) = -k + \mathbb{P}^\infty(C_1 \mid \emptyset) \times b$ varies like $\mathbb{P}^\infty(C_1 \mid \emptyset)$, and prove that $\mathbb{P}^\infty(C_1 \mid \emptyset)$ is a strictly decreasing function of θ. Indeed, in a PBE, $\hat{\delta}_1^b(\theta)$ must be smaller than 1, and this function is then equal to:

$$\mathbb{P}^\infty(C_1 \mid \emptyset) = \frac{1}{1 + \frac{\mathbb{P}(\hat{\delta}_1^b(\theta) > \delta)\pi_\emptyset^L}{\mathbb{P}(\delta \geq \hat{\delta}_1^b(\theta))\pi_\emptyset^H}}.$$

As a function of θ, $\mathbb{P}^\infty(C_1 \mid \emptyset)$ varies like:

$$\left(\frac{\mathbb{P}(\hat{\delta}_1^b(\theta) > \delta)\pi_\emptyset^L}{\mathbb{P}(\delta \geq \hat{\delta}_1^b(\theta))\pi_\emptyset^H}\right)^{-1} = \frac{\mathbb{P}(\delta \geq \hat{\delta}_1^b(\theta))\pi_\emptyset^H}{\mathbb{P}(\hat{\delta}_1^b(\theta) > \delta)\pi_\emptyset^L}.$$

We conclude by noting first that the proportion of cooperative actors $\mathbb{P}(\delta \geq \hat{\delta}_1^b(\theta))$ is a strictly decreasing function of θ—the higher the baseline chance of being accepted, the lower the incentive to cooperate—and that the proportion of defectors $\mathbb{P}(\hat{\delta}_1^b(\theta) > \delta)$ therefore strictly increases with θ.

We note second that:

$$\begin{aligned}
\frac{\pi_\emptyset^H}{\pi_\emptyset^L}, &= \frac{1+q\theta p_1}{1-q(1-\theta)p_1}, \\
&= \frac{1+q\theta p_1}{1+q\theta p_1 - qp_1}, \\
&= \frac{1}{1-\frac{qp_1}{1+q\theta p_1}}.
\end{aligned}$$

This fraction varies like $\frac{qp_1}{1+q\theta p_1}$ (since this fraction is on the denominator, but preceded by a minus sign): it is a strictly decreasing function of θ.

$\mathbb{P}^\infty(C_1 \mid \theta)$ is therefore the product of two strictly decreasing functions of θ. Following the intermediate value theorem, the algorithm defined by condition (6.15) always yields a unique value. Indeed, if $u_T(\emptyset, 0) \leq 0$, then $u_T(\emptyset, \theta)$ will be strictly negative for every $\theta > 0$, meaning that the only possible PBE is $\sigma^{b,0}$. If in contrast $u_T(\emptyset, 0) > 0$ but $u_T(\emptyset, 1) \geq 0$, then $u_T(\emptyset, \theta)$ will be strictly positive for every $\theta < 1$, meaning that the only possibility is that choosers trust given $\emptyset$. Finally, if neither of these conditions are true, that is, if $u_T(\emptyset, 0) > 0$ and $u_T(\emptyset, 1) < 0$, then following the intermediate value theorem, we obtain a root for our function, which must be unique because it is strictly decreasing. □

6.6 Domain of existence

Proposition 6.3: Domain of existence of the baseline equilibrium

There exists a PBE $(\sigma^{b,\theta^{*,b}}, \mu^\infty)$, where $\theta^{*,b}$ is defined as in Proposition 6.2, and μ^∞ derived as in Lemma 6.6, if and only if:

$$\hat{\delta}_1(\theta^{*,b}) < 1. \quad (6.16)$$

We obtain the baseline equilibrium as long as there exists a positive fraction of would-be cooperators.

Proof. The proof is analogous, and simpler, to the proof of Proposition 5.2. We begin by noting that this condition is necessary: otherwise, every actor defects, and it is always beneficial to distrust—choosers then benefit from deviation to playing $\neg T$ given an actor of cooperative reputation $\mathcal{C}_1$.

Conversely, we show that this condition is sufficient. By construction of $\theta^{*,b}$ (Proposition 6.2), there is no beneficial deviation for choosers given an actor of empty reputation. In addition, following Lemma 6.6, there are no beneficial deviations for choosers given an actor of non-empty reputation, since $\hat{\delta}_1(\theta^{*,b}) < 1$. Finally, actors play their best response (Proposition 6.1). □

7 Implementation into Mathematica

The results obtained in the previous sections are under-specified. They notably depend on the specific distribution of time preferences μ_0, on the specific allocation of incentives performed by the institution (as captured by the relative weights β, γ and π_1), and on its effectiveness (as captured by ρ).
Here, we outline the algorithm that we use to compute our numerical results using the software Mathematica, and compare our baseline results with four different types of institution.

7.1 Motivation and general algorithm

The results obtained in the previous sections are under-specified. We have characterized two equilibria: the institution equilibrium, in which both first- and second-order cooperation occur with positive probability, and the baseline equilibrium, in which only first-order cooperation occurs, as second-order cooperation is assumed not to be observed ($p_2 = 0$). We have left one degree of liberty: these equilibria depend on the probability that choosers trust actors of empty reputation, denoted θ in both cases.

Propositions 5.1 and 6.2 provide an algorithm to determine the equilibrium value of this probability θ, in the institution and baseline equilibrium respectively. The value of θ depends on the fraction of cooperative actors—the more actors are cooperative, the easier it is to trust them blindly—which itself is a function of θ, as the probability of being trusted by default (with empty reputation) affects one's incentive to cooperate.

In this section, we use Mathematica to resolve this recursive dependency, solve the model numerically, and generate figures. For both strategy profiles, we calculate the threshold(s) defining actor strategy ($\hat{\delta}^b(\theta)$ or $\hat{\delta}_1(\theta)$ and $\hat{\delta}_2(\theta)$), as well as the normalized payoff of any actor, choosers' long-run payoff, the steady state probabilities, and the level of cooperation in the steady state. The formulas defining each of these values are given in sections 4-6. All computations leading to the plots displayed in this section, along with explanations, can be found in the Mathematica notebook titled `Social Leverage Effect.nb`, which can be downloaded here.

We consider a specific type of distribution of time preferences—a truncated normal distribution of mode μ, and standard deviation σ. We call μ the **patience of the population**. Below, in Figures S3-S9, we fix $\sigma = 0.25$ and vary μ between 0 and 1, to represent the variation of our outputs with the patience of the population—μ varies on the x-axis of each graph below.

We analyze our model in five cases: the baseline equilibrium, and four cases of the institution equilibrium, corresponding to four different ways of allocating institutional incentives—a rewarding institution, where contributions are exclusively affected to increasing the payoff of cooperators by β (obtained by setting $\gamma = \pi_1 = 0$); a punishing institution, where contributions are exclusively affected to decreasing the payoff of defectors by γ (obtained by setting $\beta = \pi_1 = 0$); a monitoring institution, where contributions are exclusively affected to increasing the probability of observation in the trust game by π_1 (obtained by setting $\beta = \gamma = 0$); and a monitoring-punishing institution, which allocates incentives equally between monitoring and punishing (obtained for $\beta = 0$, $\gamma = \frac{1}{2}(\rho f_2 c_2)\frac{q}{1-q}$, $\pi_1 = \frac{1}{2}(\rho f_2 \frac{c_2}{c_1})\frac{q}{1-q}$, where f_2 is the fraction of actors that contribute to the institution when given the chance). Each time, we compute outputs for an inefficient ($\rho = 1/3$) and efficient institution ($\rho = 3$).

We fix $q = 0.5$ and $r = 2$, normalizing the maximum payoff $q \times r$ of one actor interaction, and set $p_1 = 0.25$. The difficulty of cooperation is then $\delta^b = c_1/(p_1 q r) = 4 \times c_1$.

In each graph below, we vary the cost of cooperation c_1 between 0 and 1 in order to vary the difficulty of cooperation δ^b between 0 and 4—δ^b varies on the y-axis of each of figures. This leaves plenty of room for improvement with the institution, since, following Proposing 6.3, the baseline equilibrium is impossible when $\delta^b \geq 1$.

We set $b = 1$, thus also normalizing chooser payoffs, and assume that the cost of trust k is equal to the cost of cooperation c_1 at any point in the graph. By varying the cost of cooperation between 0 and $1 = q \times r$, we also vary the cost of trust between 0 and $1 = b$.

We take $p_2 = 3 \times p_1 = 0.75$ and $c_2 = c_1/3$. By default, second-order cooperation is three times as observable, and three times less costly, than first-order cooperation. Because we are interested in institutions for extending the scope of cooperation, we assume that, even after accounting for the incentives produced by the institution, second-order cooperation remains less costly and more observable than first-order cooperation. Specifically, we assume that:

$$c_2 \leq \mathbf{c_1} = c_1 - (\beta + \gamma), \quad (7.1)$$

$$p_2 \geq \mathbf{p_1} = p_1 + \pi_1. \quad (7.2)$$

This guarantees $(c_2/p_2) \leq (\mathbf{c_1}/\mathbf{p_1})$, allowing us to define $\hat{\delta}_2(\theta)$ using only the top line of condition (4.7).

7.2 Algorithm for the baseline equilibrium

As we saw in Proposition 6.2, the equilibrium value of θ is uniquely defined for any set of parameters in the baseline equilibrium. The algorithm for doing so is provided by condition (6.15): we calculate the payoff of a chooser that trusts given empty reputation in the steady state as a function of θ, using our general formula introduced above. This is a strictly decreasing function of θ. If this payoff is negative even for $\theta = 0$, then we deduce that $\theta^{*,b} = 0$. If this payoff is positive even for $\theta = 1$, then we deduce that $\theta^{*,b} = 1$. Otherwise, we find a unique $\theta^{*,b} \in (0, 1)$ by using a bisection algorithm.

Once we have obtained the value of $\theta^{*,b}$ as a function of a set of parameter values, we can determine whether the baseline equilibrium exists for that set of parameter values (Proposition 6.3), and, if so, the value for the level of cooperation (Lemma 6.4), as well as the normalized payoff of any actor (Lemma 6.1) and the long-run chooser payoff (Lemma 6.7).

7.3 Algorithm for the institution equilibrium

The institution equilibrium characterized in section 4 depends on the specific allocation of incentives performed by the institution, as well as the distribution of discount factors.

We consider the four types of institution defined above: rewarding, punishing, monitoring, and monitoring-punishing. This last example is the one used to make the Figures presented in the main document.

In each case, we calculate the fraction of potential contributors f_2 as a function of θ, and deduce the value of all relevant variables as a function of θ. This includes for instance, in the case of the monitoring-punishing institution, determining the payoff of a defector, $r - \gamma$, and the value of the likelihood of observation, $p_1 + \pi_1$, as a function of θ.

We check that the payoff of a chooser that trusts given empty reputation in the steady state is a decreasing function of θ in our entire parameter region, and then apply the same algorithm as above to deduce the value of θ^* (see Proposition 5.1).

Once we have obtained the value of θ^* as a function of a set of parameter values, we can similarly determine whether the institution equilibrium exists for that set of parameter values (Proposition 5.2), and, if so, the value for the level of cooperation (Lemma 4.7), as well as the normalized payoff of any actor (Lemma 4.3) and the long-run chooser payoff (Lemma 5.3).

7.4 Level of cooperation

7.4.1 Baseline equilibrium

In Figure S3, we plot the level of cooperation obtained in the baseline equilibrium.

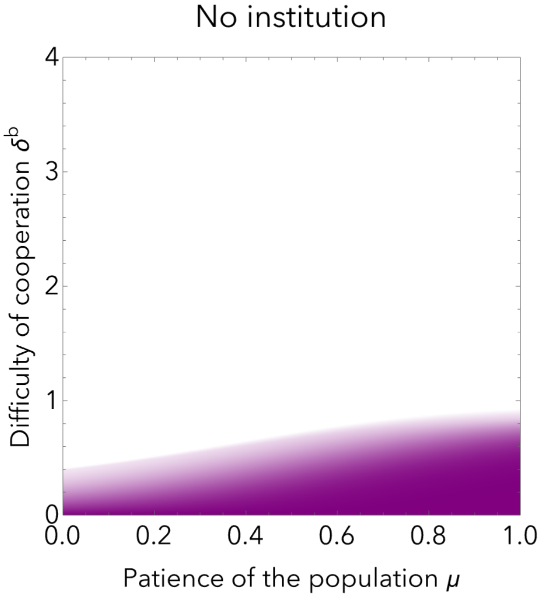

Figure S3: Level of cooperation in the baseline equilibrium, where choosers do not observe second-order cooperation by definition ($p_2 = 0$), as a function of the patience of the population μ and the difficulty of cooperation δ^b. We fix $q = 0.5$, $r = 2$, $p_1 = 0.25$, $b = 1$, $\sigma = 0.25$. We vary the difficulty of cooperation δ^b between 0 and 4 by varying $c_1 = (p_1 q r)\delta^b = \delta^b/4$ between 0 and 1. We take $k = c_1$—in any point in the graph, the cost of trust is equal to the cost of cooperation.

In this graph—as in all graphs indicating the level of cooperation—the shade of purple indicates the level of cooperation at a given point: purple indicates a level of cooperation of 1, and white indicates a level of cooperation of 0. We obtain this graph, as well as all the other graphs presented in this supplementary document using using Mathematica's DensityPlot function.

We use the parameters defined above, and vary the patience of the population μ between 0 and 1 on the x-axis, and the difficulty of cooperation δ^b between 0 and 4 on the y-axis.

7.4.2 Institution equilibrium

In Figure S4, we plot the level of cooperation obtained in the institution equilibrium for the first three cases, i.e. the rewarding (top), punishing (middle), and monitoring (bottom) institutions. In each case, we consider the inefficient and efficient variant of the institution, by fixing $\rho = 1/3$ (left column) and $\rho = 3$ (right column). To generate these six graphs (two per case; one case equals one row), we fix all parameters as above, and again vary μ between 0 and 1, and δ^b between 0 and 4.

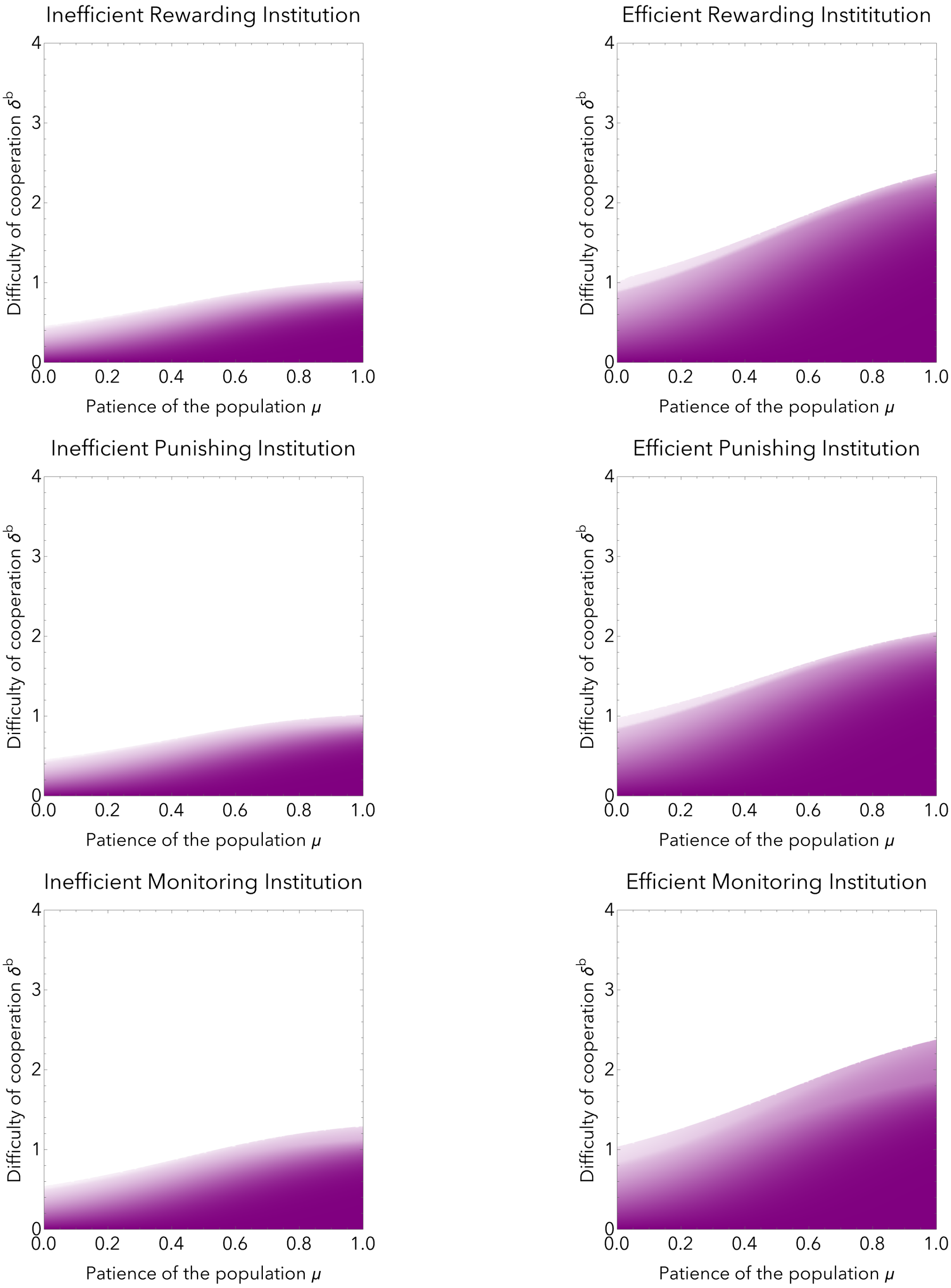

Figure S4: Level of cooperation in the institution equilibrium, for a rewarding institution (top row, obtained for $\gamma = \pi_1 = 0$), a punishing institution (middle row, obtained for $\beta = \pi_1 = 0$), and a monitoring institution (bottom row, obtained for $\beta = \gamma = 0$). In each case, results are computed as a function of the patience of the population μ and the difficulty of cooperation δ^b, for an inefficient institution (left column, $\rho = 1/3$), and an efficient institution (right column, $\rho = 3$). We take the same parameters as in Figure S3, and similarly vary δ^b between 0 and 4 by varying $c_1 = k = \delta^b/4$ between 0 and 1. We also take $c_2 = c_1/3$ and $p_2 = 3p_1$.

Finally, we plot the level of cooperation obtained for a monitoring-punishing institution in Figure S5, again for $\rho = 1/3$ and $\rho = 3$.

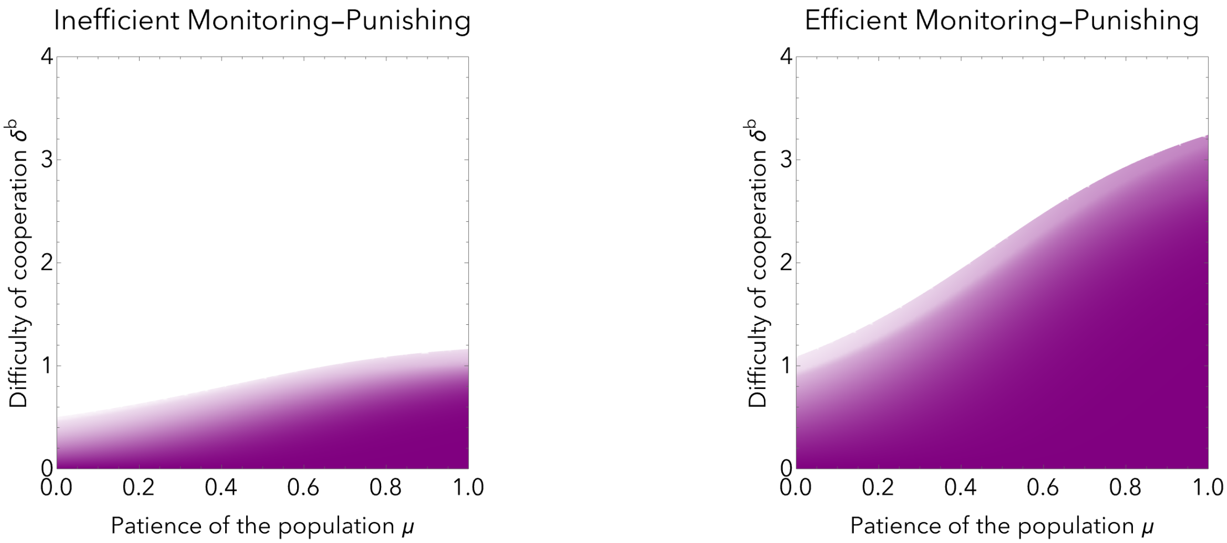

Figure S5: Level of cooperation in the institution equilibrium obtained for a monitoring-punishing institution ($\beta = 0$, $\gamma = 1/2(\rho f_2 c_2)(1-q)/q$, $\pi_1 = 1/2(\rho f_2 c_2/c_1)(1-q)/q$), as a function of the patience of the population μ and the difficulty of cooperation δ^b. Left: inefficient institution ($\rho = 1/3$); right: efficient institution ($\rho = 3$). We take the same parameters as in Figure S4.

7.5 Comparison between the monitoring-punishing institution and no institution

7.5.1 Increase in the level of cooperation

In each case, the level of cooperation is higher in the institution equilibrium than it is in the baseline equilibrium—and the difference is starker when the institution is more efficient (high ρ). To illustrate the effect of an institution on cooperation, we subtract the level of cooperation in the baseline equilibrium to the level of cooperation in the institution equilibrium in the case of a monitoring-punishing institution. We plot the resulting increase in the level of cooperation in Figure S6, for $\rho = 1/3$ (left panel) and $\rho = 3$ (right panel).

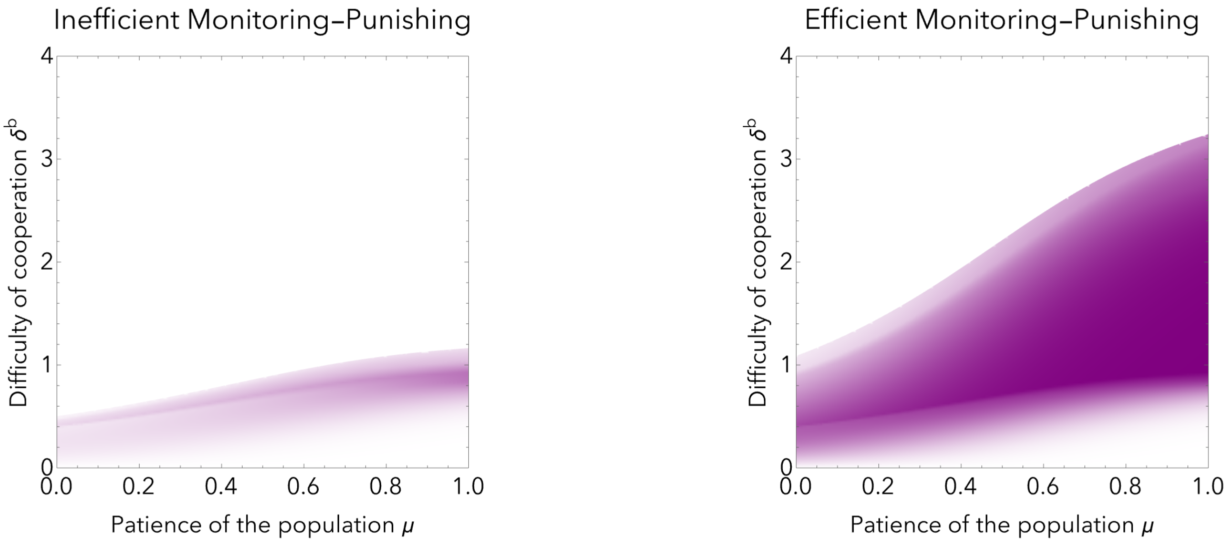

Figure S6: Increase in the level of cooperation due to the monitoring-punishing institution, as a function of the patience of the population μ and the difficulty of cooperation δ^b. To compute this value, we subtract results plotted in Figure S3 to those plotted in Figure S5, for an inefficient monitoring-punishing institution (left, $\rho = 1/3$) and an efficient one (right, $\rho = 3$).

This time, the shade of purple indicates the increase in the level of cooperation at a given point: purple indicates an increase of 1, and white indicates an increase of 0.

7.5.2 Change in chooser and actor payoffs

Even an efficient institution (right of Figure S6) leads to only a marginal increase in the level of cooperation when δ^b is low and μ is high—in such a case, the level of cooperation is already high without an institution. The institution may in fact lead to a decrease in actors' payoffs in a similar parameter space, as actors then pay the cost of second-order cooperation c_2 in a context where costly enforcement of first-order cooperation is largely unnecessary. In contrast, the institution will always increase choosers' payoffs, since they do not bear the costs of second-order cooperation.

To illustrate, we use the normalized actor payoff and the long-run chooser payoff defined previously. We first compute the expected actor payoff—defined as the expected value of the normalized payoff of any actor, previously calculated as a function of her discount factor δ (Lemmas 4.3 and 6.1), when discount factors are drawn according to distribution of actor time preferences. We normalize this value by dividing by $q \times r$, the maximum payoff of one interaction. We then subtract the expected actor payoff in the baseline equilibrium from the expected actor payoff in the institution equilibrium, for the efficient and inefficient monitoring-punishing institutions.

We proceed similarly for the long-run chooser payoff. We normalize by dividing by b, and subtract the long-run chooser payoff in the baseline equilibrium to the long-run chooser payoff in the institution equilibrium, in the same cases. We plot our results in Figure S7.

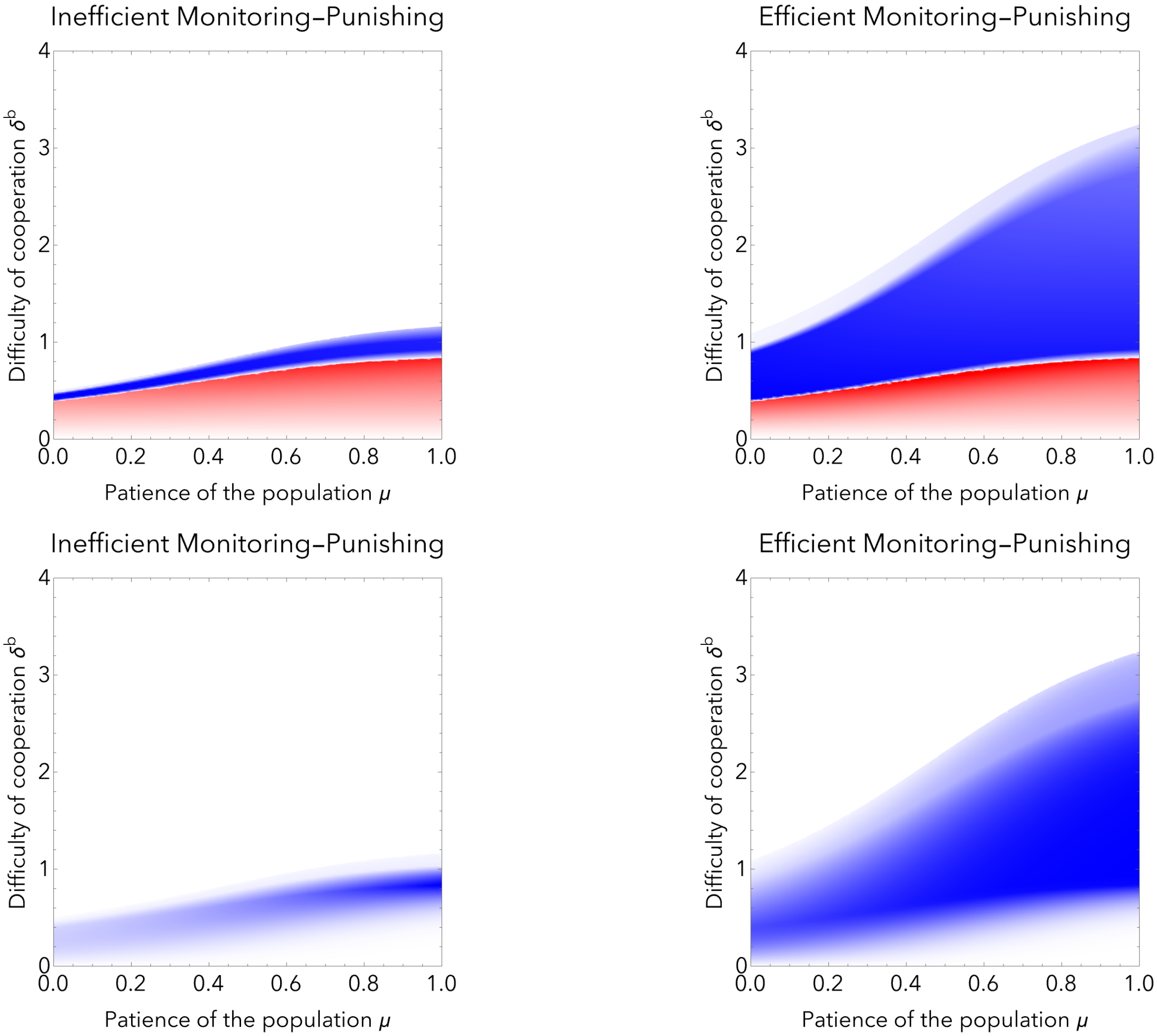

Figure S7: Change in actor (top row) and chooser (bottom row) payoff due to the monitoring-punishing institution, as a function of μ and δ^b. We use the same parameters as in Figure S4, and compare payoffs in the institution equilibrium to those obtained in the baseline equilibrium. Shades of blue indicate an increase in actor or chooser payoff, on a scale of 0 to 1. Shades of red indicate a decrease in actor payoff, on a scale of 0 to 0.05. These small decreases are explained by noting that, actors who contribute to the institution pay on average $(1-q)c_2 = c_1/6$ throughout their life. In regions where the institution is unnecessary, c_1 is small, and $c_1/6$ is very small.

Shades of blue indicate an increase in payoffs: dark blue indicates an increase of 1, and white an increase of 0. Shades of red indicate a decrease in expected actor payoff: dark red indicates an increase of 0.05 or more.

As visible on the top row of Figure S7, actor payoffs decrease where c_1 is small. The institution leads to them paying costs, which they do not recoup in those cases. Their payoff decrease is small, explaining our choice of scale, as actors who contribute to the institution pay on average $(1-q)c_2 = c_1/6$ throughout their life. However, When c_1 is large, actor payoffs increase, as the institution allows them to establish cooperative relationships with choosers (as visible by comparing with the results for the baseline equilibrium, represented in Figure S3).

As visible on the bottom row of Figure S7, chooser payoffs increase everywhere, as actors pay to increase the cooperativeness of other actors.

7.5.3 Change in total payoff

We carry out the same computation for the expected payoff of any individual—defined by averaging between the actor and chooser payoffs used above. Note that we weigh the expected actor payoff by $1/(1+q)$ and the long-run chooser payoff by $q/(1+q)$ to capture the fact that actors play in both games and choosers only in one game—weighing them equally would lead to a lower estimation of the red zone in which total payoffs decrease, since only actors pay the cost of second-order cooperation.

We plot the result in Figure S8. In regions in which the actor expected payoff decreases, this decrease is partially compensated by an increase in chooser long-run payoff. As a result, the maximum net decrease for the expected payoff is just above 0.006, or 0.6% of the maximum value. To visualize these decreases, we further shrink the red scale—dark red now indicates a decrease of 0.01 or more. As before, dark blue indicates an increase of 1.

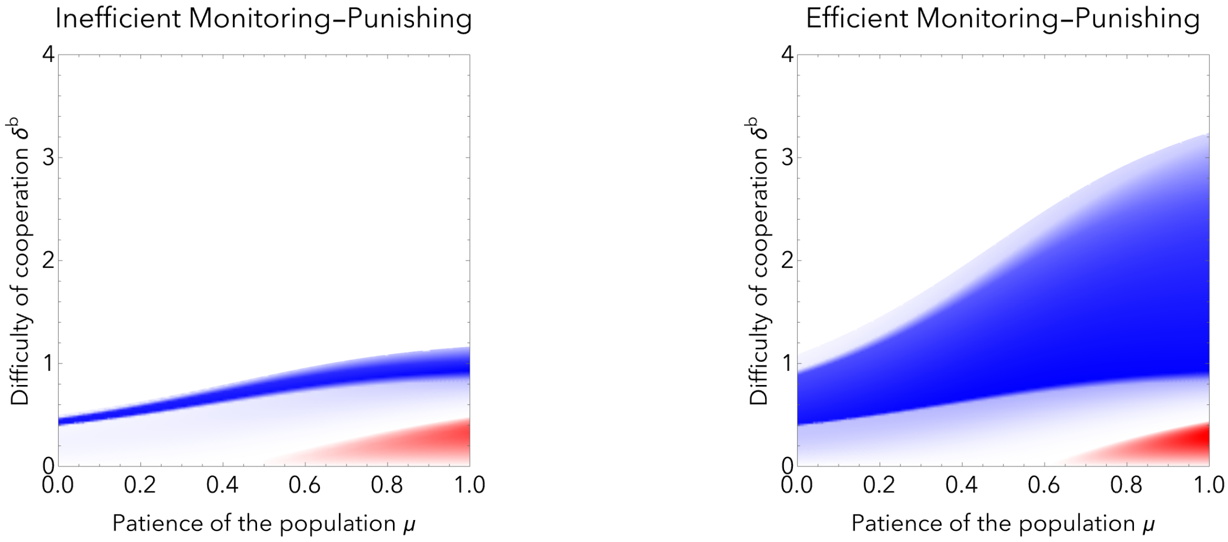

Figure S8: Change in expected payoff due to the monitoring-punishing institution, as a function of the patience of the population μ and the difficulty of cooperation δ^b, for an inefficient institution (left, $\rho = 1/3$) and an efficient institution (right, $\rho = 3$). We use the same parameters as in Figure S4, and compare payoffs in the institution equilibrium to those obtained in the baseline equilibrium. Shades of blue indicate an increase, on a scale of 0 to 1, i.e., 100% of the maximum value. Shades of red indicate a decrease, on a scale of 0 to 0.01, i.e., 1% of the maximum value.

7.6 Difference in payoffs due to the rewarding, punishing and monitoring institutions

The monitoring-punishing institution can be wasteful. Regardless of its efficiency, this institution leads to a payoff decrease where cooperation is easy (low δ^b) and the population is patient (high μ), as shown in the bottom right of both panels in Figure S8. In this parameter region, high levels of cooperation can already be achieved without the institution; paying to monitor and punish then leads to a payoff decrease.

To examine this in more detail, we look at the change in expected payoffs due to the rewarding, punishing, and monitoring institutions. Results are plotted in Figure S9, in the efficient and inefficient cases. Only an efficient rewarding institution (top right panel) increases payoffs throughout our domain, as small contributions are converted into bigger rewards. Any other institutional arrangement, including an inefficient rewarding institution (top left panel), leads to a payoff decrease where cooperation is easy and the population is patient.

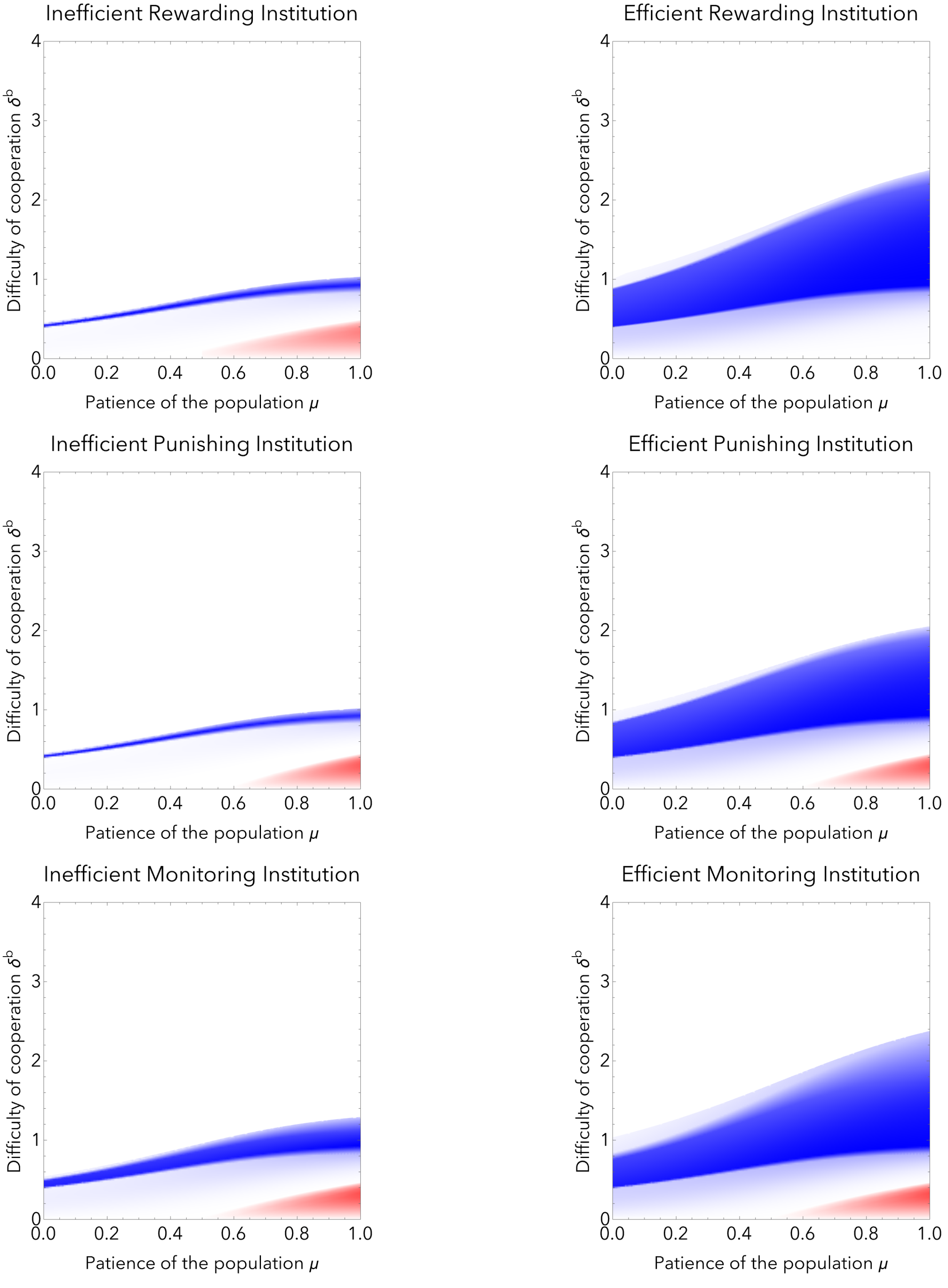

Figure S9: Change in expected payoff due to a rewarding institution (top row, obtained for $\gamma = \pi_1 = 0$), a punishing institution (middle row, obtained for $\beta = \pi_1 = 0$), and a monitoring institution (bottom row, obtained for $\beta = \gamma = 0$). In each case, results are computed as a function of the patience of the population μ and the difficulty of cooperation δ^b, for an inefficient institution (left column, $\rho = 1/3$), and an efficient institution (right column, $\rho = 3$). We use the same parameters as in Figure S4, and compare payoffs in the institution equilibrium to those obtained in the baseline equilibrium. Shades of blue indicate an increase, on a scale of 0 to 1, i.e., 100% of the maximum value. Shades of red indicate a decrease, on a scale of 0 to 0.01, i.e., 1% of the maximum value.

References

(1) George Joseph Mailath and Larry Samuelson. Repeated Games and Reputations: Long-Run Relationships. Oxford ; New York: Oxford University Press, 2006. 645 pp. ISBN: 978-0-19-530079-6.

(2) Drew Fudenberg and Jean Tirole. Game Theory. MIT Press, Aug. 29, 1991. 616 pp. ISBN: 978-0-262-30376-7.

(3) Reinhard Selten. "Evolutionary Stability in Extensive Two-Person Games". In: Mathematical Social Sciences 5.3 (Sept. 1983), pp. 269–363. ISSN: 01654896. DOI: 10.1016/0165-4896(83)90012-4. URL: https://linkinghub.elsevier.com/retrieve/pii/0165489683900124 (visited on 11/02/2020).
